# Computational study of HIV gp120 as a target for polyanionic entry inhibitors: Exploiting the V3 loop region

**DOI:** 10.1371/journal.pone.0190658

**Published:** 2018-01-18

**Authors:** Louis R. Hollingsworth, Anne M. Brown, Richard D. Gandour, David R. Bevan

**Affiliations:** 1 Department of Chemical Engineering, Virginia Tech, Blacksburg, Virginia, United States of America; 2 Department of Biochemistry, Virginia Tech, Blacksburg, Virginia, United States of America; 3 Department of Chemistry, Virginia Tech, Blacksburg, Virginia, United States of America; 4 Research and Informatics, University Libraries, Virginia Tech, Blacksburg, Virginia, United States of America; 5 Virginia Tech Center for Drug Discovery, Virginia Tech, Blacksburg, Virginia, United States of America; Università degli Studi di Milano, ITALY

## Abstract

Multiple approaches are being utilized to develop therapeutics to treat HIV infection. One approach is designed to inhibit entry of HIV into host cells, with a target being the viral envelope glycoprotein, gp120. Polyanionic compounds have been shown to be effective in inhibiting HIV entry, with a mechanism involving electrostatic interactions with the V3 loop of gp120 being proposed. In this study, we applied computational methods to elucidate molecular interactions between the repeat unit of the precisely alternating polyanion, Poly(4,4′-stilbenedicarboxylate-*alt*–maleic acid) (DCSti-*alt*-MA) and the V3 loop of gp120 from strains of HIV against which these polyanions were previously tested (IIIb, BaL, 92UG037, JR-CSF) as well as two strains for which gp120 crystal structures are available (YU2, 2B4C). Homology modeling was used to create models of the gp120 proteins. Using monomers of the gp120 protein, we applied extensive molecular dynamics simulations to obtain dominant morphologies that represent a variety of open-closed states of the V3 loop to examine the interaction of 112 ligands of the repeating units of DCSti-*alt*-MA docked to the V3 loop and surrounding residues. Using the distance between the V1/V2 and V3 loops of gp120 as a metric, we revealed through MD simulations that gp120 from the lab-adapted strains (BaL and IIIb), which are more susceptible to inhibition by DCSti-*alt*-MA, clearly transitioned to the closed state in one replicate of each simulation set, whereas none of the replicates from the Tier II strains (92UG037 and JR-CSF) did so. Docking repeat unit microspecies to the gp120 protein before and after MD simulation enabled identification of residues that were key for binding. Notably, only a few residues were found to be important for docking both before and after MD simulation as a result of the conformational heterogeneity provided by the simulations. Consideration of the residues that were consistently involved in interactions with the ligand revealed the importance of both hydrophilic and hydrophobic moieties of the ligand for effective binding. The results also suggest that polymers of DCSti-*alt*-MA with repeating units of different configurations may have advantages for therapeutic efficacy.

## Introduction

Currently, over 37 million people worldwide are living with HIV, with an additional 7000 new infections daily [1]. In the absence of a vaccine and with the continuing spread of the virus, a pressing need remains for new preventative strategies, including microbicides [2]. Because they inhibit viral entry and are biocompatible, polyanions have been tested as gel-formulated microbicides [3, 4]. Polyanions, such as commercially available PRO2000 and dextran sulfate, have shown excellent results in preclinical, Phase I, and Phase II clinical trials; however, Phase III clinical trials have been largely unsuccessful [3, 5–7]. These polyanions have been experimentally shown to inhibit viral entry by binding to the highly positive V3 loop region of the envelope glycoprotein (Env) on the surface of HIV [3, 8–12]. As current microbicides under development are primarily reverse transcriptase and integrase inhibitors [7] and do not include polyanionic entry inhibitors, one might expect that a well-designed polyanion could overcome the pitfalls of previous polymers to provide a low-cost agent to add to the microbicidal cocktail.

Poly(4,4′-stilbenedicarboxylate-*alt*–maleic acid) (DCSti-*alt*-MA), a precisely alternating polyanion, shows excellent but varying *in vitro* anti-HIV activity (IC_50_’s 10–100 nM) against four HIV-1 strains (92UG037, IIIb, JR-CSF, and BaL) [13]. As a semi-rigid polyanion with a 6-nm statistical segment length, [13, 14] DCSti-*alt*-MA is predicted to bind to the V3 loop region of Env; however, the inhibitory mechanism remains unresolved. This polymer provides an excellent scaffold from which to create more potent polyanionic entry inhibitors. *In silico* methods such as molecular docking and molecular dynamics (MD) simulations provide atomistic details of binding of the polyanion to Env and will contribute to the design of these inhibitors.

Env is a highly glycosylated trimer of heterodimers, comprised of the surface protein gp120 and transmembrane protein gp41 [15–17]. Sequence differences in variable loop regions of gp120 are observed in various HIV-1 strains [17]. Variable loop regions contribute significantly to the structural flexibility of gp120, revealed by high B-factors in crystal structures, often necessitating the complete removal of loop regions to achieve high resolution structural refinement [18–20]. Env mediates viral entry by binding to the cellular CD4 receptor and undergoing a conformational change from a “closed” to “open” state, characterized by global rearrangement of the V1/V2 loops from the inside to the periphery of the trimer to expose the V3 loop to cellular receptors [21–25]. Open state Env then binds a chemokine co-receptor, either CCR5 or CXCR4, triggering a cascade of conformational changes that results in the formation of a post-fusion six-helix bundle and, ultimately, cellular entry [26–28]. Through electrostatic interactions, the highly variant and approximately 35-residue V3 loop on gp120 plays a role in mediating co-receptor specificity and tropism [29, 30]. These interactions have led to the proposed “11/25 rule”, where charges in the 11^th^ or 25^th^ V3 loop residues determine co-receptor specificity [31].

Previous MD simulations of gp120 monomers and trimers show that the V3 loop of HIV may sample multiple conformations dictated in part by net charge [32–36]. More recently, simulations of fully glycosylated, pre-fusion (closed state) Env trimers reveal details concerning the binding of a novel anti-fusion peptide antibody [37] and glycosylation in multiple viral clades. [16] Experimental studies of Env show that three prefusion, closed state conformations exist [38] and transient sampling of open state may occur [39–41]. Despite burgeoning structural and dynamical data on Env, full-length HIV strain-specific dynamics remain unexplored, even though the motions of variable loop regions and antibody epitopes [42] are relevant to *in silico* drug design. As HIV strains evolve to escape neutralization by current drugs, computational simulation is critical to resolve the conformational states of the V3 loop, particularly due to the complexities of resolving the entire conformational space of Env [43]. In other words, what conformation(s) is (are) best for screening drug candidates?

Herein, we generated models of gp120 from four strains (gp120_BaL_, gp120_IIIb_, gp120_JR-CSF_, gp120_92UG037_) of HIV using comparative structure (homology) modeling, and we built in the loop regions of gp120_YU2_ from its abridged crystal structure, 2QAD [18]. Due to conformational sampling of the flexible V3 loop region, we conducted MD simulations on these five models, in addition to the crystal structure template, gp120_2B4C_, to obtain conformational ensembles. We also conducted molecular docking studies of repeat units (ligands) of DCSti-*alt*-MA to the six models of gp120, both before and after MD simulations. Further, we sought to elucidate how conformational plasticity may affect ligand binding, particularly the strain variation in IC_50_ [13]. We specifically studied how ligand binding to an apo gp120 structure both before and after dynamics simulation could provide insight into strain specificity. Such an atomistic description of Env dynamics informs the design of entry inhibitors targeting a broad range of HIV strains.

## Methods

### Homology modeling

Sequences of gp120s from HIV-1 strains were obtained from Uniprot (Figure A in S1 File) [44]. The coordinates for the gp120 monomer were extracted from the 2B4C (gp120_2B4C_) [18] and 2QAD [19] (gp120_YU2_) crystal structures. As alternate positions were present in the 2B4C crystal structure for residues 312–315, a single conformer of these residues was selected. These crystal structures contain the V3 loop in the open state but are missing the V1/V2 loop regions (Table A in S1 File). Homology models of gp120_92UG037_, gp120_BaL_, gp120_IIb_, and gp120_JR-CSF_ were generated in Molecular Operating Environment (MOE) [45] using crystal structure 2B4C as template. The missing V1/V2 loops were modeled into these homology models and into the crystal structure of gp120_YU2_ (2QAD) using MOE to obtain full length gp120s (Table A in S1 File). In subsequent MD simulations, the 2B4C crystal structure (gp120_2B4C_), with a missing V1/V2 loop region, was used for comparison with the full-length models, particularly to assess local and V3 loop fluctuation from an unmodified crystal structure. All homology models, including the modified (gp120_YU2_) crystal structure, were energy minimized in MOE [45] with Amber12EHT [46] parameters and validated with Ramachandran plots [47], ANOLEA [48], and QMEAN [49] (Figures C-H in S1 File) by using RAMPAGE [47] and the SwissModel [50] suite of tools.

### Molecular dynamics simulations

The GROMACS v5.0.5 software suite [51, 52] was used for all MD simulations. Systems were built and independently simulated with both the June 2015 release of CHARMM36 [53] (Charmm) and Amber99SB-ILDN [54] (Amber) force fields for all six proteins. Systems included the TIP3P [55] explicit water model in cubic boxes with a minimum solute-box distance of 1.0 nm. Each system was neutralized with Na^+^ counter ions and contained 150 mM NaCl (Table B in S1 File).

Energy minimization was performed with the steepest descent algorithm with a maximum force constraint of 1000 kJ/mol*nm, and position restraints were imposed on all heavy atoms, prior to MD simulations. Each system was equilibrated at constant volume and temperature (NVT) of 300 K (Charmm) or 310 K (Amber) for 100 ps with the Berendsen weak coupling method [56]. These temperatures were chosen to replicate prior trimer simulations (Charmm) [16, 37] or physiological temperature (Amber). Next, isothermal-isobaric conditions (NPT) were imposed by using a modified Berendsen thermostat [56] and 1 bar with a Parrinello-Rahman [57] barostat for an additional 100 ps (Charmm) or 500 ps (Amber).

All production MD simulations used periodic boundary conditions with restraints removed. A short-range cutoff of 1.2 nm (Charmm) or 0.8 nm (Amber) was applied to all nonbonded interactions, as recommended for these force fields [53, 54]. Long-range electrostatic interactions were calculated with the smooth particle mesh Ewald (PME) method [58, 59] by using cubic interpolation and a Fourier grid spacing of 0.16 nm (Charmm) or 0.12 nm (Amber). An integration time step of 2 fs was used, using the fourth order P-LINCS algorithm [60] to constrain all bonds. MD simulations were run in triplicate for 600 ns for each protein with two force fields, resulting in 36 total simulations (21.6 μs), providing 100 ns of sampling after convergence (500 ns). The last 100 ns of each simulation was used for subsequent analysis. Trajectories were analyzed with block averaging, root-mean-square deviation (RMSD), root-mean-square fluctuation (RMSF), and principal component analysis (PCA), by using both GROMACS [53] and in-house scripts. A representative structure from each of the 36 simulations was obtained by clustering over the final 100 ns by using the GROMOS algorithm and a 0.2 nm RMSD cutoff [61]. Visual analysis was conducted with PyMOL [62] and Chimera [63]. V1/V2 to V3 loop distances were measured from their respective centers of mass. Quaternary structure fitting was conducted by first aligning structures to a fitted trimer in PyMOL (PDB ID: 5FUU [15]) followed by further fitting of monomeric units in the corresponding electrostatic density map with Chimera (EMDB: 3308 [15]).

### Molecular docking

Ligands, repeat units of DCSti-*alt*-MA, consist of 16 possible stereoisomers, based on the number of stereocenters (Figure I in S1 File). Each stereoisomer could exist in 7 ionization states (microspecies) (Figure I in S1 File), though the two trianions would likely dominate under physiological conditions of pH 7.4 (Figure I in S1 File). For completeness, all 112 stereoisomers/microspecies were docked to the six gp120 models. Ligands were generated by drawing 16 stereoisomeric tetraanions in a WebMO [64] interface for Gaussian 09 [65] followed by initial geometry optimization by AM1 [66] with the PCM solvent model for water [67]. The dianionic and trianionic ligands were created by adding protons to the tetraanionic ligands for each configuration. As with the tetraanions, the geometry of each microspecies was optimized by AM1 (PCM = water) and conformationally analyzed to identify global minima. These structures were saved as pdb files for input as ligands to the docking program.

Histidine protonation states for protein structures were determined in Chimera [63] (considering local hydrogen bonding) prior to further standard protein and ligand file preparation in Autodock Tools (ADT) [68] for molecular docking. Ligands were docked into the crystal structure of gp120_2B4C_ with Autodock4 [68], allowing for ligand conformational flexibility. The lowest energy ligand conformations of 100 were selected for additional geometry optimization with AM1 (PCM = water) to ensure that all low energy conformers were used in subsequent docking studies.

Autodock Vina [69] was employed to generate 20 poses and to calculate binding energies for each of the 112 ligands (16 stereoisomers each with 7 microspecies). A grid spacing of 1.000 Å was used, with the center of the grid box placed at the approximate center of either the entire protein (denoted as “Largebox” in Table C and E in S1 File) or the V3 loop region in ADT. Details on box size and position are found in Tables C-E in S1 File. Initially, a box was generated around the entire protein for proof-of-concept of specific binding to the V3 loop region (Table E in S1 File) followed by a smaller search space constrained to solely the V3 loop (Table D in S1 File). Ligands were docked into the six models both before and after MD simulation with analysis focusing on data from the docking to the V3 loop region. Representative cluster structures for each of the three MD replicates of each viral strain were used for docking. Ligand–protein fingerprint analysis [70, 71] was conducted on the lowest energy pose of every ligand (112 per protein) with Schrödinger v2017-1 [72]. Interaction cutoffs were set as 4.0 Å among heavy atoms and 2.5 Å with hydrogen atoms.

## Results

### Evaluation of gp120 structures

Structures of gp120 from crystal structures (gp120_2B4C_ and gp120_YU2_) and homology models (gp120_BaL_, gp120_IIb_, gp120_JR-CSF_, gp120_92UG037_) were evaluated. The four homology models were from strains of HIV against which DCSti-*alt*-MA was studied experimentally [13]. Evaluation of these six structures revealed the expected dihedral angles and energy scores as based on Ramachandran plots and ANOLEA scores. ANOLEA scores in the V3 loop region of all structures were unfavorable, and in some cases, dihedral angles in the loop were in unfavorable regions (Figures C-H in S1 File), though outliers in loop regions are not unusual. The nature of homology modeling is such that the models are biased towards the structure that is used as the template. Thus, even with variation in the sequence of the V3 loop (Figure A in S1 File), homology models showed highly similar backbone positions in the V3 loop (Figure B in S1 File), consistent with gp120 homology models in the literature [36].

**Fig 1:**
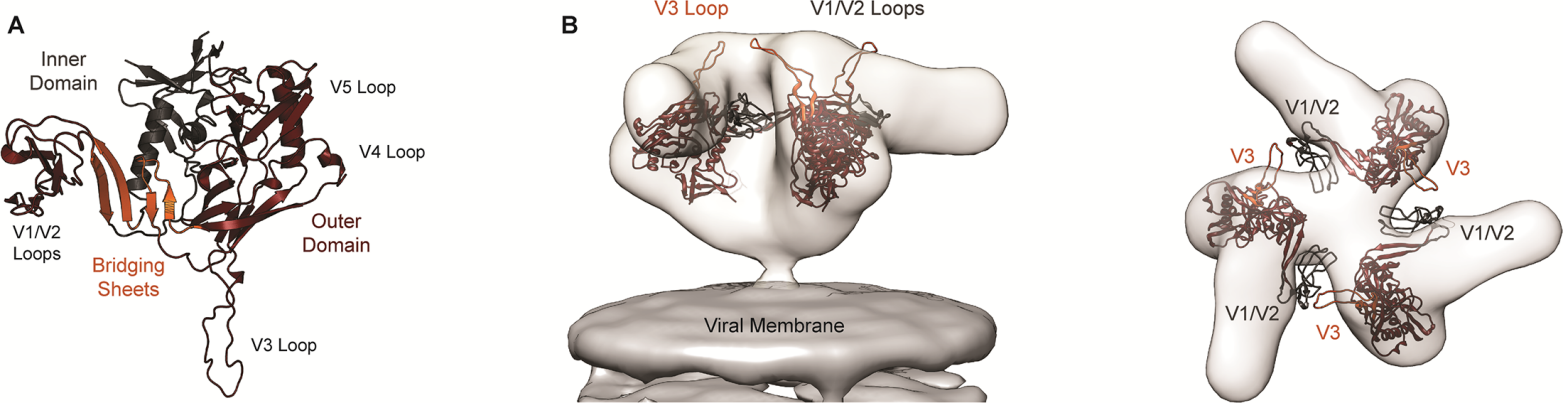
**(A)** gp120_YU2_ homology model with labeled variable loop regions and colored by domain: inner (charcoal), outer (maroon) and bridging sheet (orange). (**B)** gp120_YU2_ homology model fitted to electron microscopy density data (EMDB 5018 [76], 5553 [77]) from a side view (left) and top-down (right) perspective, demonstrating the trimeric arrangement of gp120 protomers and illustrating the orientation of the V1/V2 (charcoal) and V3 (orange) loop regions relative to each other and the viral membrane.

We used monomeric models to examine differences among strains tested, but quaternary structure can also influence and impose restraints upon CD4 binding [73] and conformational stability [74]. Nevertheless, the V1/V2 and V3 loops play a role in viral entry, and their relative orientations shift during entry [75]. Thus, to better understand and validate our monomeric models relative to quaternary structure, we fit three gp120_YU2_ monomers into the electron density map of Env [15, 76, 77] (Fig 1). These models revealed the accessibility of the loops on the surface of the structure as well as the proximity of the V1/V2 loop in one monomer to the V3 in the adjacent monomer.

### Ligands bind to residues on the V3 loop

Knowing that chiral, charged polymers present a complex computational challenge, we modeled the repeat unit of DCSti-*alt*-MA (ligands) by terminating each end with a methyl group (Figure I in S1 File). This approach produced sixteen stereoisomeric ligands; each stereoisomer comprised seven charged microspecies (dianions, trianions, and tetraanion), which are likely present at pH = 7.4 (Figure I in S1 File). In total, 112 ligands were docked to the six gp120 proteins in Autodock Vina. This extensive modeling approach ensured a complete survey of possible ligand–protein interactions.

**Fig 2:**
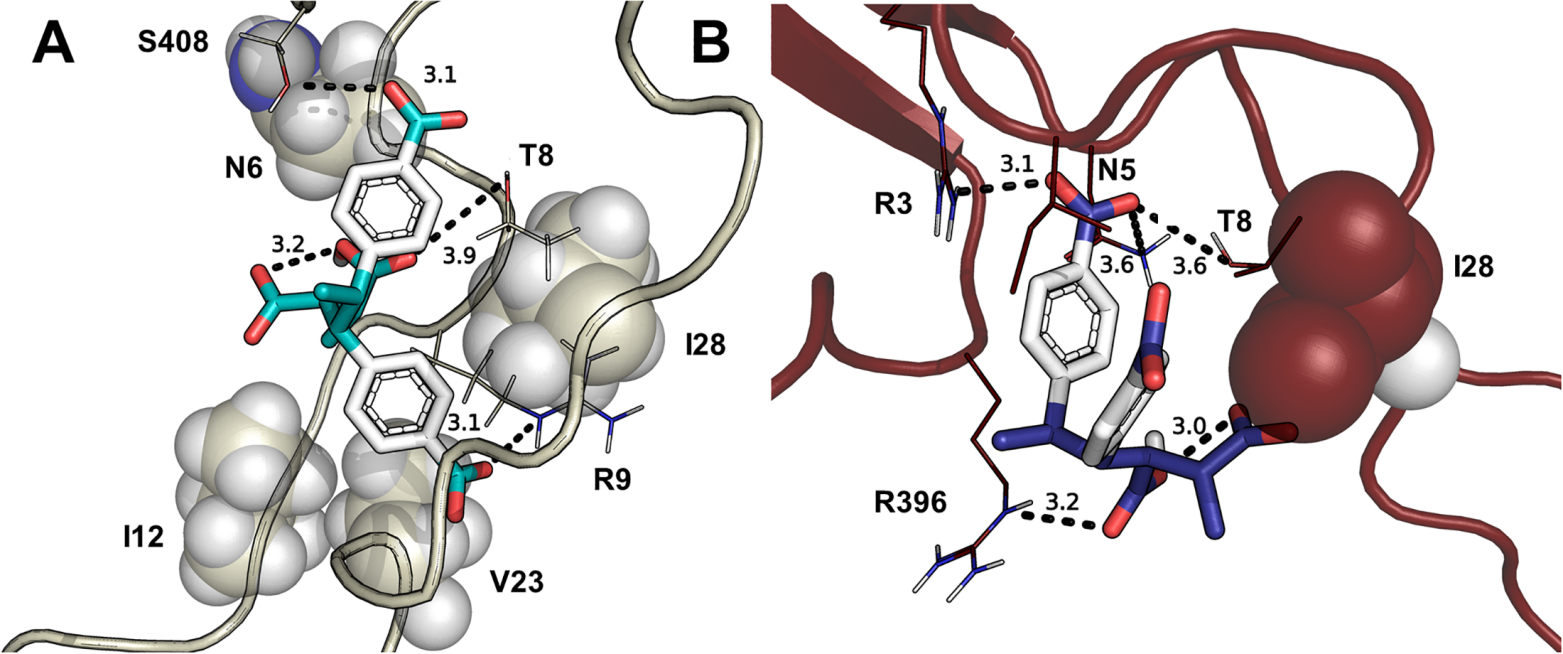
Binding of ligands to various strains of gp120. **(A)** gp120_IIIb_ with RSSS3NO. The ligand contains a 3.1 Å internal hydrogen bond, and interacts with R9 (3.1 Å), T8 (3.9 Å), and S408. **(B)** gp120_YU2_ with RSSR3NI. The ligand has a 3.0 Å internal hydrogen bond and interacts with N7 (3.6 Å), T8 (3.6 Å), R3 (3.0 Å) and R396 (3.2 Å).

Initially, we conducted a search of ligand docking to the entire protein for the six gp120 structures and observed binding to the V3 loop region for all ligand configurations. As the V3 loop is considered the target for polyanions (references 3 and 8–12), analysis focused on docking to the V3 loop of the six proteins—gp120_2B4C_, gp120_BaL_, gp120_IIIb_, gp120_JR-CSF_, gp120_92UG037_, and gp120_YU2_. Representative poses were viewed in PyMOL (Figure J in S1 File) and initially analyzed with Vina energy scores (Figure K in S1 File), which encompassed a narrow range variation (< 2.4 kcal/mol across all ligands, < 1.2 kcal/mol among the best poses of each strain). The binding affinity for the 6 proteins ranked as follows: gp120_BaL_ > gp120_92UG037_ > gp120_IIIb_ > gp120_2B4C_ > gp120_JR-CSF_ > gp120_YU2_ (Figure K in S1 File). Binding scores revealed enantioselectivity and stereoselectivity among the ligands. The majority of high-scoring docking poses involved ligands as trianions and dianions (Fig 2 and Figure J in S1 File), which dominate at pH 7.4 because ionization to the tetraanion has a p*K*_a_ of 10 in the polymer [14]. In most poses, the strong internal hydrogen bond between the carboxyls on the maleic acid moiety of the dianions and trianions was preserved after docking.

To address ligand–protein interactions with each gp120, fingerprint analyses (Figures L-Q in S1 File) elucidated residues that were key in binding (Table 1). The ligands mainly interacted with conserved residues in the V3 loop region (Table 1). In all models, ligands interacted hydrophilically or with hydrogen bonding to T8 and hydrophobically with I/M32. Other key residues, for which interactions were observed in at least four of the gp120 structures, were R3, N/S5, N/Q6, R9, G26, and I28. Key residues not in the V3 loops that interacted with the ligands are located in and around the bridging sheets (Fig 1). In most cases, these residues represented a small number of interactions relative to those with V3 loop residues, though for gp120_YU2_, a notable number of interactions in the bridging sheet region was observed (P393, P394, I395, R396).

**Table 1:** Key residues, numbered based on aligned V3 loop sequences (Figure A in S1 File) that interact with ligands based on fingerprinting analysis (Figures L-Q in S1 File). Residues identified interact with > 50% of ligands and >40% if denoted with an asterisk.

gp120	IC_50_ (ng/mL)[13]	H-Bond	Hydrophilic	Hydrophobic	Backbone
2B4C	NA	R3, R9*	Q6, T8	P4, I32, P438	N5, R440
92UG037	750		R3, T8, R9, Q34, Q401	P4, I28, I32, P399	N5, N6, D31, I400
BaL	230		R3, N6, T8, R9, Q34, R405	P4, I28, I32, P403	N5, N7*, G26*, I404 G406
IIIb	95	T8, R9	N6, S408	I12, V23, I29, M32	N5, T24, I25, G26
JR-CSF	760		N6, T8, R9, E27, K401	I28, I32	T24, G26
YU2	NA	R396	R3, N5, T8	I28, I32, P393*	G26, P394, I395

NA = not available.

These initial docking studies were done using a single conformation of the protein, as crystal structures and homology models represent a static snapshot. Some studies have reported the advantage of docking to an ensemble of structures as a way of accounting for the inherent flexibility in protein structures [78, 79]. Given the importance of the fluctuation of the V1/V2 and V3 loops in gp120 function, we did extensive MD simulations to generate other conformations of the protein that might be relevant for binding of inhibitors, after which docking (Table E in S1 File) and fingerprinting analysis (Figures R-II in S1 File) were again conducted with the representative clusters obtained from MD simulations.

### MD simulations of gp120 structures with two force fields

Several previous MD simulations of monomeric or trimeric gp120 or of the V3 loop have been conducted using Amber force fields, primarily ff99SB (*e*.*g*., [32, 35, 36, 80]). With continued development of force fields, we also wanted to apply a more recently refined force field, specifically CHARMM36 as well as a more recent Amber force field (99SB-ILDN). As a result, we conducted MD simulations with both of these force fields on the six gp120 structures (gp120_2B4C_, gp120_92UG037_, gp120_BaL_, gp120_IIIb_, gp120_JR-CSF_, gp120_YU2_).

Convergence in the simulations was evaluated by using RMSD plots (Figures JJ and KK in S1 File) and RMSD block averaging (Tables H and I in S1 File). These criteria indicated convergence across at least two replicates of every system, particularly as reflected in the small standard deviations in the block average values. The highly flexible loop regions in gp120 likely contributed to the fluctuation of RMSD, even at 600 ns, for some of the replicates. The contribution of the V1/V2 and V3 loop regions to the dynamics of the gp120 models also was evident from the RMSF measurements, which revealed that the highest fluctuations were in these regions of the proteins (Figures LL and MM in S1 File). Notably, no systematic differences were observed between results obtained with either the CHARMM36 or the Amber 99SB-ILDN force fields when comparing values for RMSD and RMSF. Even in cases for which some fluctuation was apparent in the RMSD graphs, the block averaging results were indicative of convergence.

### Structural rearrangements with V1/V2 and V3 loop regions

**Fig 3:**
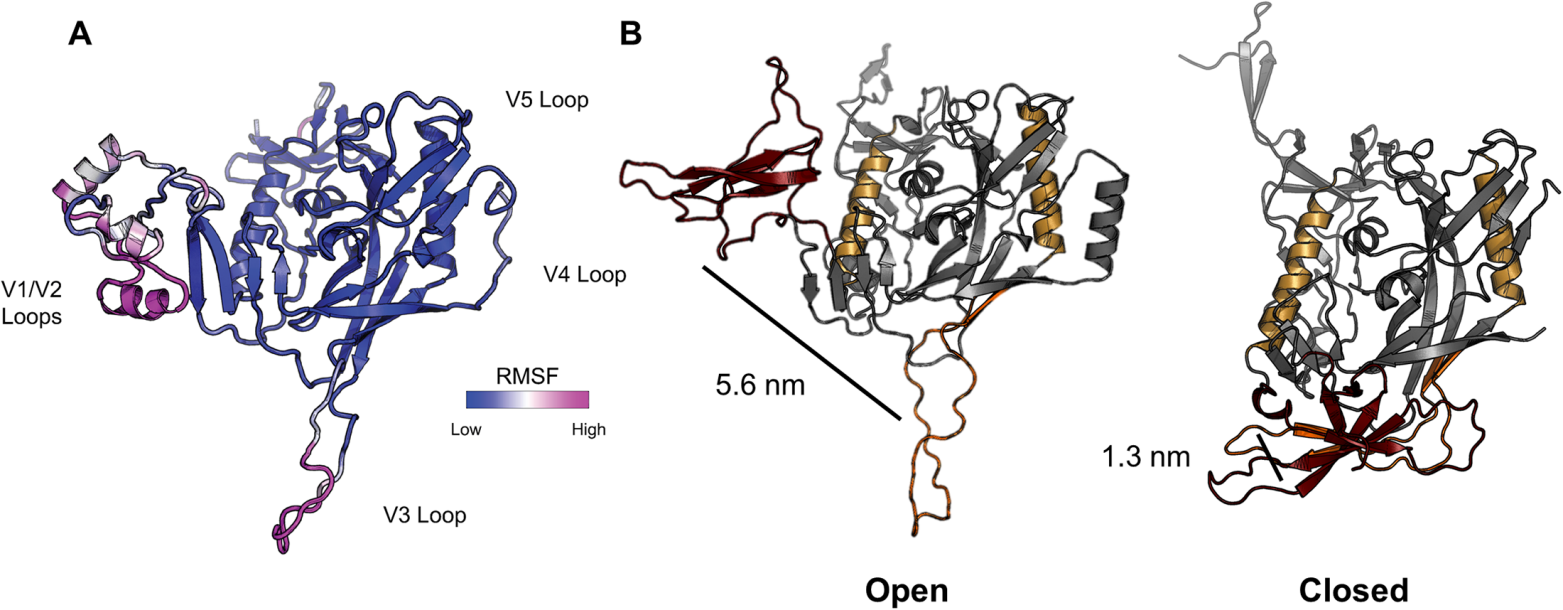
**(A)** gp120_IIIb_ homology model colored by RMSF following 600 ns of MD simulation (replicate 1). V1/V2 and V3 loop regions account for the primary structural fluctuations throughout the simulation, representative of the general dynamic behavior of all gp120 simulations (Figures LL and MM in S1 File). **(B)** Distance between the COM of V1/V2 loops (maroon) and the V3 loop (orange) in open (3J70 [20], left) and closed (4TVP [81], right) state gp120. The COM distance increases significantly following the conformational change from the closed to open state. Reference points are shown in gold.

Based on the above observations, a more thorough analysis of structural transitions in gp120 during the MD simulations was conducted. Closed and open states of gp120 are typically described based on changes in the quaternary structure of the trimer [22, 76]. In addition, the transition between open and closed states of gp120 includes movement of the V1/V2 and V3 loops relative to one another, which we used as a way to monitor transitions between open and closed states during the MD simulations of the monomer (Fig 3). To develop a quantitative metric for the open and closed states of gp120, five crystal structures containing at least portions of the V1/V2 and V3 loops in conformations ranging from closed to open were assessed. Based upon initial overlays of these structures, we created an open/closed state metric dependent on V1/V2 to V3 loop distance (Table L in S1 File). The relative center of mass (COM) distance between V1/V2 loops and the V3 loop was used to define the structures as being closed (< 3 nm) or open (> 3 nm) for subsequent analysis (Fig 3).

All simulations began with the gp120 structures in the open state, which is the conformation obtained from modeling. Examination of the graphs of the V1/V2 to V3 distances revealed that in most of the simulations, the structures remained in the open state based on the > 3 nm distance criterion (Figures PP and QQ in S1 File). However, in a few cases, the structures transitioned to the closed state, in some cases abruptly and early in the simulations (Figure PP in S1 File) and in other cases, more gradually. Observing the conformational transition suggested that MD will be useful for examining the dynamics of gp120, though longer simulations likely are needed to observe repeated transitions between the conformational states. Also of note is that major differences were not observed between the force fields. With both force fields, a transition from the open to the closed state was observed to a limited extent.

**Fig 4:**
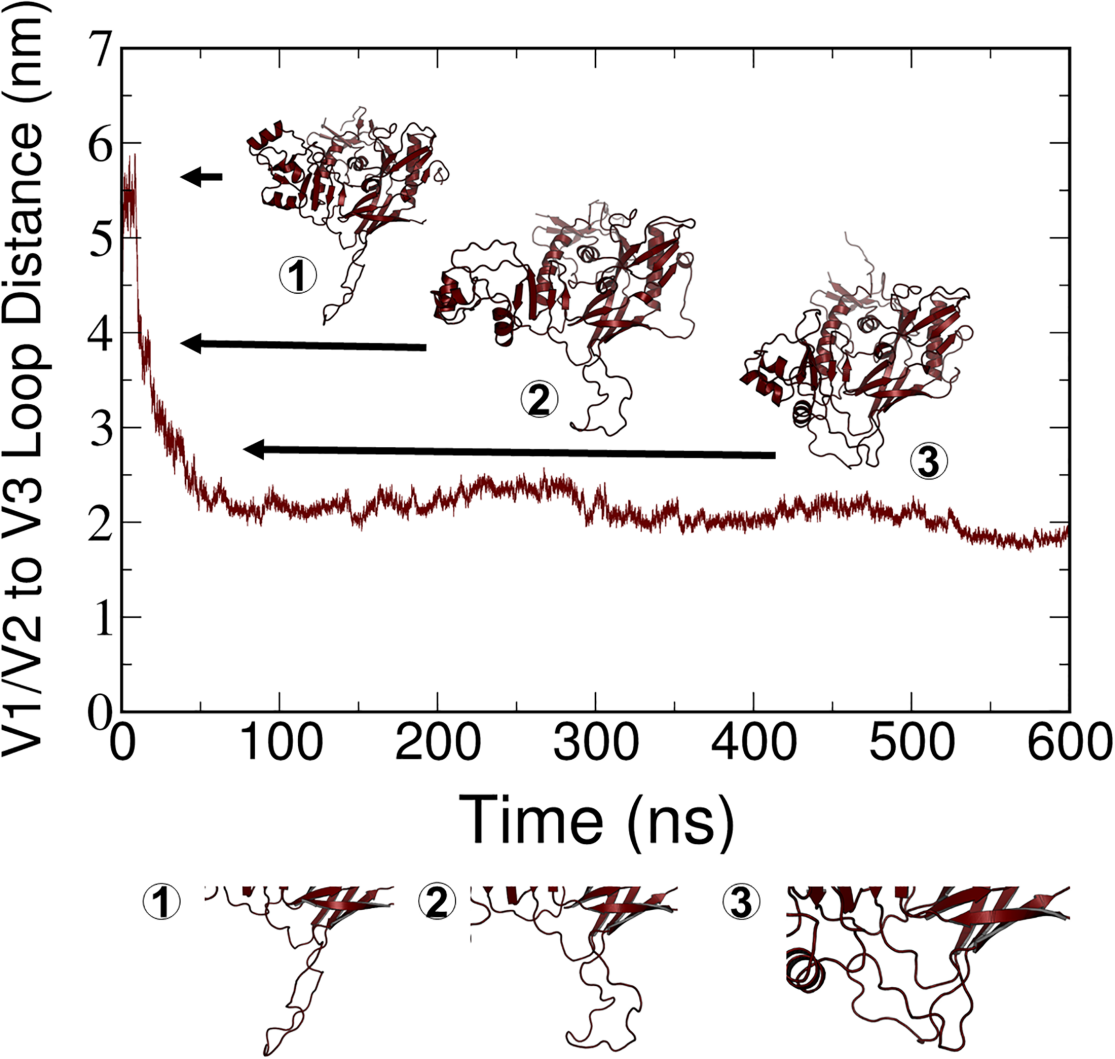
Results from MD simulation of gp120_IIIb_ (replicate 2) with the CHARMM36 force field. A clear transition from the open to closed state is apparent from the decrease in distance between the V1/V1 and V3 loops and is illustrated in the corresponding structures.

**Fig 5:**
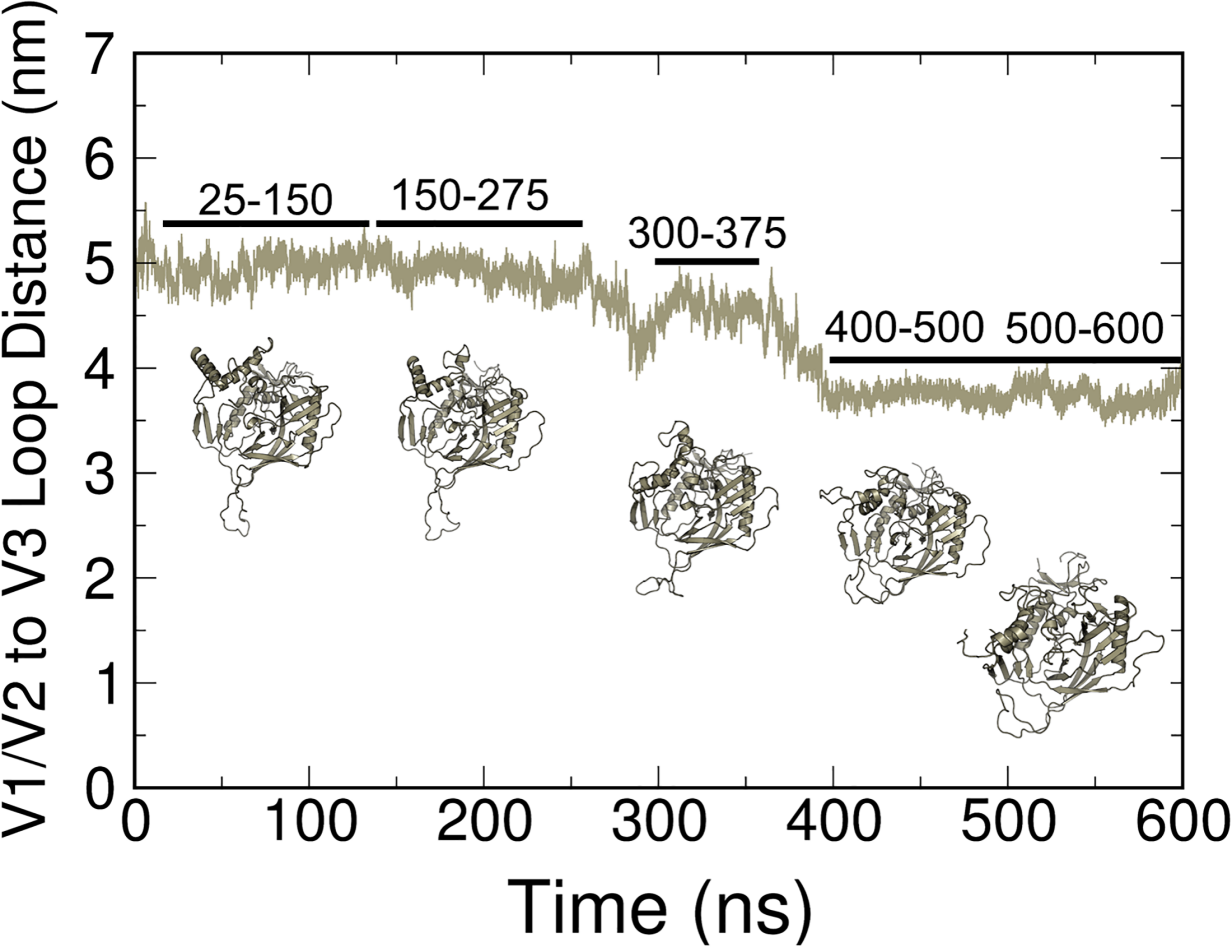
Representation of a partially open state in MD simulations. COM between the V1/V2 and V3 loop regions and representative clusters from the time frames that are indicated in the graph.

Although a distinct transition between open and closed states occurred in a few cases, as illustrated in Fig 4, several cases also were observed in which the V1/V2 to V3 loop distance decreased from the open state observed in the initial structures to a distance that was not below the 3-nm criterion for a closed state (Figure PP and QQ in S1 File). We classified these states as being “intermediate” between open and closed conformations. Based on RMSD criteria, these intermediate states are stable over at least the last 100 ns of simulation within the time period and conditions of our simulations. A closer examination of one of these cases is provided in Fig 5. The decreasing distance between the V1/V2 and V3 loops in one of the simulations is graphed along with structures that correspond to those loop distances that represent these partially open conformations. To better visualize the structural fluctuations, principal component analysis (PCA) of trajectories by using both Charmm (SM1) and Amber (SM2) force fields was conducted. This analysis shows that the concerted hinge motion of the V1/V2 loops away (closed to open transition) or towards (open to closed transition) the V3 loop accounts for the primary global protein motion in each of the gp120 proteins. Although we did not perform a quantitative comparison of these structural fluctuations from PCA, the importance of dynamics in the loop regions was apparent.

### Docking to gp120 structures after MD simulation

To select structures for docking following MD simulation, structures were clustered (0.2 nm RMSD cutoff) over the last 100 ns of each trajectory (Figures NN and OO in S1 File). The percentage of structures that were contained within the dominant cluster varied between force fields and among replicates, as would be expected from independent simulations (Tables J and K in S1 File). The representative structures from the dominant clusters showed some conformational heterogeneity (Figures NN and OO in S1 File), which is advantageous for docking studies. Because large differences were not observed in the dynamics of the gp120 structures when considering simulations with CHARMM or Amber, docking after MD simulation was done only with structures from the CHARMM simulations (Figure NN in S1 File).

The 112 ligands (16 enantiomers times 7 microspecies) were docked to the three replicates for each of the six gp120 structures, for a total of 2016 docking runs. The docking focused on the V3 loop and nearby residues that were included in the box centered on the center of mass of the V3 loop. Fingerprinting analyses (Figures R-II in S1 File) revealed which residues were interacting with each of the ligands. A list of those residues, selected based on frequency of interaction and categorized by strain, replicate number, and type of interaction is provided in Table F in S1 File. This list was assembled from the histograms at the top of the fingerprint analyses (Figures R-II in S1 File), mimicking the analysis of the pre-docking fingerprint interaction analysis. Protein-ligand interactions were much more heterogeneous for docking following MD simulations, as a result of the increased number of protein conformations to which ligands were docked (Figures NN in S1 File). The key residue interactions from docking to structures following MD simulation are summarized in Table G in S1 File. A comparison of key interactions pre-MD (Table 1) and post-MD (Table G in S1 File) reveals distinct similarities and differences. Residues that were key for docking to both pre-MD and post-MD structures are G26, I28, and I/M32. The occurrence of these residues for pre-MD and post-MD docking suggests an importance for hydrophobicity in docking the repeat unit of DCSti-*alt*-MA to gp120 proteins. Residues R3, N/S5, T8, and R9, which are key residues when docking to pre-MD structures, do not participate in docking to the post-MD structures to any great extent. Additional interacting residues for post-MD structures often are unique to a gp120 from only one of the strains, further reflecting the heterogeneity among these post-MD structures. An interesting example is the gp120 from strain IIIb, in which R11 (S11 in the other strains) is observed to interact with the ligands in post-MD docking. Strain IIIb is the only CXCR4 tropic strain in this study, and it is the strain most strongly inhibited by DCSti-*alt*-MA in the experimental studies [13]. This additional positive charge in the V3 loop of gp120_IIIb_ may render this strain more susceptible to this polyanion inhibitor.

## Discussion

Multiple approaches are being investigated to identify therapeutics that will prevent HIV infection. Among these approaches is the search for HIV entry inhibitors, those compounds that can prevent the fusion and entry of HIV into host cells. One class of these compounds is designed to interrupt the binding of the gp120 domain of HIV Env to the CD4 receptor on the host cell, with this binding being fundamental to the subsequent processes involved in fusion. An early example of this type of inhibitor is denoted NBD-556, which has been shown to bind in the Phe43 cavity of gp120 [82]. A subsequent generation of this type of compound is based on guanidinium-containing trans-1,2-indanes, for which the interactions with gp120 have been characterized [83]. The polyanionic polymers that are the subject of this study represent another class of HIV entry inhibitors. Based on experiments, these compounds are proposed to interact via electrostatic interactions with the variable V3 loop of gp120, which is positively charged. Early examples of these compounds included sulfonated and sulfated polyanions, though some of these compounds were reported to enhance infection rates by disrupting the integrity of mucosal cell membranes [7].

An alternative class of polyanionic polymers are those containing carboxylate moieties. The previous experimental study upon which this current modeling study is based considered the anti-HIV properties of chemically synthesized carboxylated alternating copolymers that have been carefully characterized in terms of their molar masses, polydispersity index, and degree of polymerization [13]. These compounds protected against HIV infection in HeLa cells and human peripheral blood mononuclear cells. To gain insight into the mechanism of action of these compounds, the current modeling study was undertaken. In this initial characterization of the molecular details of the interaction between these polyanions and gp120, monomeric units of the polyanion were examined for their interaction with gp120 monomers from different strains of HIV. Structural insight into strain-specific gp120-ligand interactions may therefore enhance the efficacy of therapeutics targeting HIV. We recognize the challenges that can arise when simplifying the systems to their fundamental units, but we present these results as our first step towards modeling the system in greater complexity. By docking to pre- and post-MD structures, we can begin to understand initial events in polyanion-V3 loop binding and look for differences among strains to elucidate differences in strain response.

A challenge in initiating these studies was the lack of high resolution structures of gp120 that included all of the variable loops. Using homology modeling, we generated models of the gp120 proteins of interest and evaluated their structural properties using several assessment metrics. Although not available when we began our studies, one structure has since been deposited in the PDB (3J70) that includes complete V1/V2/V3 loops [20]. This structure was generated using partial crystal structures, coarse-resolution electron microscopy models, and homology modeling. The models generated through homology modeling in our study were comparable to the 3J70 structure and provided us with a set of gp120 structures from different HIV strains to study dynamics and ligand binding.

MD simulations have been applied to a limited extent in studies of the gp120 protein. In one study, simulations were conducted of only the V3 loop, in which the ends of the loop were connected by a disulfide bond between Cys residues at the termini [84]. A comprehensive analysis of the loop dynamics was conducted, though the results are not directly comparable with our studies given that the remainder of the protein was not included in the simulations. Prior MD simulations have been performed on gp120, but limitations in these studies included the absence of V1/V2 loop regions in most gp120 crystal structures [18, 19, 85, 86]. A number of other MD simulations have been done to gain insight into the dynamics of the V3 loop within the context of the larger gp120 structure. Limitations of these studies are that they are primarily of short duration (< 30 ns) and typically consist of only a single MD run, thereby reducing the possibility for thorough conformational sampling. While these studies included much of the gp120 structure and the V3 loop, notably the V1/V2 loop region was absent. Nevertheless, the studies were designed and executed to answer questions related to some aspect of V3 loop dynamics. For example, MD simulations of gp120 in the CD4-bound and unbound states revealed that unbound gp120 has greater conformational diversity [34]. Another study was designed to understand how mutations in the V3 loop contributed to the resistance to maraviroc, an HIV-1 entry inhibitor [87]. The results from MD simulations revealed that these mutations altered dynamics within the V3 loop, which could contribute to the resistance. The role of glycosylation on V3 loop dynamics also was examined using MD simulation, with the results showing that glycosylation reduced the extent of conformational sampling of the loop [35]. In our study, we did not include glycosylation of the V3 loop, and the absence of glycans could alter the conformational states that were sampled and the orientation of docked poses. However, given the potential heterogeneity of glycosylation, it is not straightforward to construct relevant models for gp120 from multiple strains. Our current outcomes provide the foundation for future work in which the complexities of glycosylation can be considered. The importance of charged residues within the V3 loop was noted above, and MD simulations also have been done to examine the effect of charge on V3 loop dynamics. In a study using monomeric gp120, it was observed that charge alone could alter fluctuations and conformational sampling within the V3 loop [36].

Our studies expanded upon these MD simulations of the gp120 monomer in that they were done for a much longer simulation time and multiple replicates of each simulation were performed, thereby providing much greater conformational sampling. It is recognized that the functional unit of the Env protein is a trimer, but focusing our initial studies on the monomer allowed for this extensive sampling. Our fitting to the trimer structure further demonstrates the trimeric arrangement with our monomeric models and gives insight into individual monomeric influence on structure-function relationships. One MD simulation of a trimer consisting of the gp120-CD4 complex has appeared in which it was reported that the V3 loops within the trimer exhibited different conformational dynamics, which was attributed to the effects of electrostatic potential acting between the subunits of the trimer [32]. Experimental studies of the conformational dynamics of the soluble Env trimer also can be related to our work. The experimental approaches include H-D exchange [39, 88] and single molecule fluorescence resonance energy transfer [38, 89], both of which provide dynamic information about the proteins in solution. Cryo-EM of the soluble trimer in complex with CD4 and/or antibodies also reveals the presence of multiple conformational states [81, 90]. An outcome from these experimental studies that is particularly relevant to our research was the observation of multiple conformational states that are classified as open, partially open (or intermediate), and closed.

As our simulations consisted of a monomer, we could not apply the same definition of conformational states that was used in experimental studies of the trimer. However, by using a monomer that included all of the variable loops, including V1/V2/V3, we were able to devise an index for open, intermediate, and closed states that was based on the movement of the V1/V2 and V3 loop regions relative to one another. Single-molecule fluorescence resonance energy transfer (smFRET) data suggest three distinct closed state Env conformations exist and differ in occupancy between HIV strains. [38] Taken together with PCA (S1 and S2 Movies), RMSF (Figures LL and MM in S1 File), and conformational heterogeneity (Fig 5), these results strongly support that open and closed state transitions on Env take place spontaneously in a broad range of HIV-1 strains. Herein, we demonstrate that gp120 conformation is dynamic across six simulated viral strains, and that this conformational sampling is responsible for global protein motion across all MD simulations (Figs 4 and 5, SM1 and SM2). We also like to note that sampling of the last 100 ns of the simulations for analysis was to observe major, stable conformations. We performed RMSD on the entire protein to define convergence, as discussed in the results, but also RMSD analysis on only the V3 loop region. We recognize that while a stable conformation has been achieved, there is a caveat that other conformations, potentially similar, could also be present given the multiple, flexible loops in the gp120 structure.

**Fig 6:**
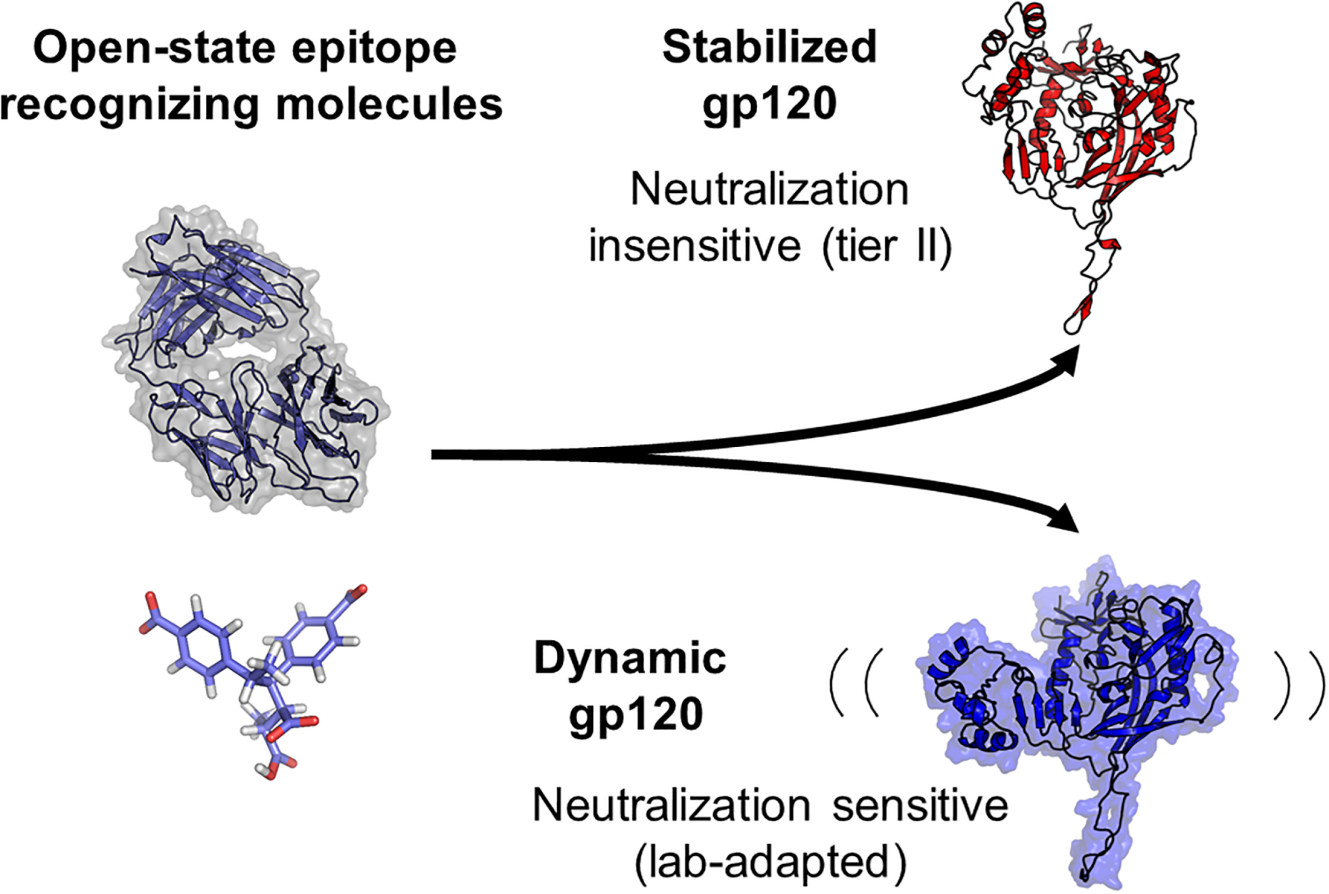
Simplified model showing the interplay between Env dynamics and drug neutralization. All Env displays some degree of transience due to low barriers of energy between prefusion conformations; however, propensity for structural rearrangement determines neutralization by molecules that recognize the open state. Highly dynamic gp120 allows transient access to open state epitopes, while less dynamic strains are less sensitive to neutralization by open state recognizing molecules.

An interesting observation of this aspect of the MD simulations is that the gp120’s from the lab-adapted strains (BaL and IIIb), which are more susceptible to inhibition by DCSti-*alt*-MA, clearly transitioned to the closed state in one replicate of each simulation set, whereas none of the replicates from the Tier II strains (92UG037 and JR-CSF) did so. It is hypothesized that globally dynamic (Fig 4) strains expose epitopes for both small and large molecules, leading to greater neutralization by molecules that recognize the open state. We propose a model, building on previous studies [39, 40, 91–93], wherein static Env are less sensitive to small molecules, such as DCSti-*alt*-MA, and antibodies that bind to open-state epitopes, such as the V3 loop, that are less exposed in closed state Env (Fig 6). Further studies with gp120’s from each type of strain are needed to more definitively establish the significance between conformational preferences and susceptibility to inhibition by this polyanionic inhibitor.

The MD simulations not only revealed conformational preferences for the gp120’s that were examined in this study, they also were used to generate conformers for molecular docking. Previous studies report an increase in docking performance by docking to MD conformational ensembles [78, 79]. Docking to an ensemble of conformers generated by MD simulation has been applied by other investigators when targeting the Phe43 region of gp120, which is part of the critical gp120-CD4 interface. For example, Li et al. [94] examined the binding of BMS-488043 to gp120 using structures selected at regular intervals from an MD simulation, but their structures had truncated V1/V2 and V3 loops, so binding to the loops could not be considered. More recently, Moraca et al. [95] used crystal structures with PDB ID codes 4NCO [96] and 4TVP [97], which include nearly intact V1/V2/V3 loops, as the basis for their studies and focused on identifying small-molecule CD4 mimetics and used a combination of approaches that included molecular docking and MD simulations. Notably, the region at which these mimetics bound was within the gp120-CD4 binding interface and away from the V3 loop that was the focus of our study. Similarly, Andrianov et al. [98] applied molecular modeling, to include pharmacophore modeling, molecular docking, and MD simulations with free energy calculations, to search for CD4 mimetics that would bind to gp120 proteins. Their study also looked at the residues in the gp120-CD4 hotspot, which is distinct from the V3 loop.

Docking of ligands to the V3 loop region of gp120 is virtually unexplored, most likely due to lack of previous structural information that has recently became more available. One study has been reported in which β-galactosylceramide was docked to 35-residue long models of the V3 loop [99]. To our knowledge, our study represents the first examination of docking of ligands to the V3 loop in the context of the full gp120 monomer structure. With the extensive sampling of protein conformer structures through multiple replicates of extended MD simulation, our studies lay the foundation for future computational and experimental studies that target the V3 loop of gp120 with potential therapeutic compounds. By using this method, we were able to identify key gp120 structure morphologies that highlight differences in open-closed states of gp120 across strains that can be used for strain-specific docking targets. Herein, these structures from post-MD, coupled with pre-MD structures, give insight into conserved residues across strains that are needed to bind DCSti-*alt*-MA.

The robustness of the proteins to dock all configurations and microspecies of the ligands without significance variance suggests that designing a ligand with a specific configuration and ionic state may not be the approach to developing an anti-HIV agent with wide-ranging activity against many HIV strains. Perhaps a polymer with random stereochemistry may be a better approach. Such a polymer would contain the repeat units that would serve as preferred ligands for gp120 proteins from different viral strains. Our observation for the role of hydrophobic residues (G26, I28, and I/M23) as key residues for interaction, in addition to polar residues (R3, N/S5, T8, and R9 or R11/S11), highlights the need for diverse physiochemical properties of ligands to bind this V3 loop region. It is predicted that this strategy will guide future design of potential therapeutics to more effectively bind the V3 loop by exploiting the known electrostatic properties and hydrophobic core. Finally, for a polymer, one needs to study a longer sequence of repeat units to explore synergies or incompatibilities of having different configurations in adjacent repeat units. The results presented herein suggest that with increasing computational power such an investigation will be possible.

### Conclusions

- We have successfully generated full-length models of gp120 structures from HIV strains IIIb, BaL, 92UG037, JR-CSF by using comparative structure (homology) modeling. The models included the V1/V2/V3 loops that are often missing in crystal structures.
- Docking of the repeat unit of DCSti-*alt*-MA to the gp120 structures prior to MD simulation revealed a set of key amino acid residues (R3, N/S5, N/Q6, T8, R9, G26, I28, and I/M32) interacting with many of the 112 ligands that were docked. Binding scores revealed enantioselectivity and stereoselectivity among the ligands.
- MD simulations generated a heterogeneous set of gp120 conformations that releveled strain-specific difference between open-closed states, providing a range of structures for docking. Due to this heterogeneity, the identification of key residues was more difficult than for docking to pre-MD structures. Nevertheless, certain residues (R9 or R11/S11, G26, I28, and I/M32) were identified as key residues from both pre-MD and post-MD docking. These residues suggest the importance of the hydrophobic character of the ligands, in addition to their polar properties, which should be exploited for strain-specific gp120 therapeutic design.
- MD simulation can be used to monitor transitions among the open, intermediate, and closed conformations of gp120. Notably, gp120 from the lab-adapted strains (BaL and IIIb), which are more susceptible to inhibition by DCSti-*alt*-MA, clearly transitioned to the closed state in one replicate of each simulation set, whereas none of the replicates from the Tier II strains (92UG037 and JR-CSF) did so.

## Supporting information

S1 File Supplemental figures and tables document. Contains 43 figures and 12 tables of supporting information as referenced in the text.
(DOCX)

S1 Movie Depiction of the first eigenvector extremes for each gp120 CHARMM36 force field simulation. The movie shows large coordinated movements of the V1/V2 and V3 loops away or towards each other in most gp120 replicates.
(WMV)

S2 Movie Depiction of the first eigenvector extremes for each gp120 Amber99SB-ILDN force field simulation. The movie again shows large coordinated movements of the V1/V2 and V3 loops in most replicates, and isolated V1/V2 loop movements in a few replicates.
(WMV)
