# Supplementary material for: Computational study of HIV gp120 as a target for polyanionic entry inhibitors: Exploiting the V3 loop region
Source: PLoS One. 2018 Jan 18;13(1):e0190658. doi: 10.1371/journal.pone.0190658 (PMC5773097; doi:10.1371/journal.pone.0190658)
Supplement: S1 File — Contains 43 figures and 12 tables of supporting information as referenced in the text. (DOCX) [file pone.0190658.s001.docx]

**­­­­Supporting Information**

**Computational Study of HIV gp120 as a Target for Polyanionic Entry Inhibitors: Exploiting the V3 Loop Region**

**Louis R. Hollingsworth IV^1-3^, Anne M. Brown^2,4^, Richard D. Gandour^3,5,^ and David R. Bevan^2,5,^*.**

^1^Department of Chemical Engineering, Virginia Tech, Blacksburg, VA, USA

^2^Department of Biochemistry, Virginia Tech, Blacksburg, VA, USA

^3^Department of Chemistry, Virginia Tech, Blacksburg, VA, USA

^4^Research and Informatics, University Libraries, Virginia Tech, Blacksburg, VA, USA

^5^Virginia Tech Center for Drug Discovery, Virginia Tech, Blacksburg, VA, USA

*Corresponding author:

David R. Bevan

Email: [drbevan@vt.edu](mailto:drbevan@vt.edu)

**Homology Modeling**

Few structures of gp120 that contain the full length V1/V2 and V3 loop regions are available on the RCSB PDB.^1^ Two open state structures containing the V3 loop but an abridged V1/V2 loop region are both bound to CD4 and an antibody (PDB ID: 2QAD^2^ and 2B4C^3^). The 2QAD structure is the YU2 strain and the 2B4C structure is the JR-FL strain. As these structures are 3.3 Å resolution, precise knowledge of sidechain atom location, particularly of the flexible V3 loop region, is unknown. Loop building with the Molecular Operating Environment (MOE)^4^ program was necessary to construct the V1/V2 loop regions due to truncation that facilitated crystallization in these strains. Additionally, experimental structural information of the V3 loop region on CXCR4-tropic viral strains, which differ substantially from CCR5 tropic strains in their net charges, are largely understudied. Therefore, simulations on multiple strains of HIV and binding analysis on several conformational ensembles is desirable for drug design.


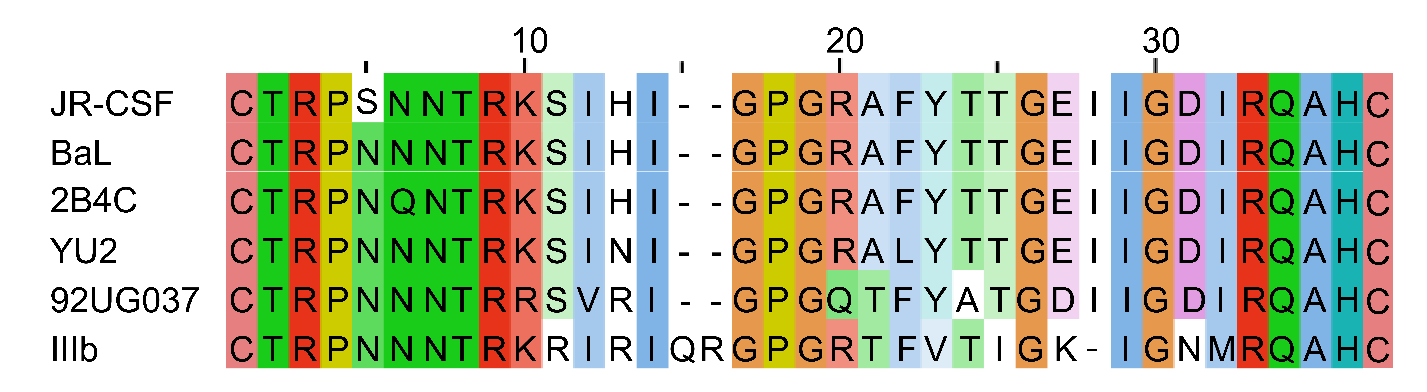


**Figure A.** Multiple sequence alignment of the V3 loops from the simulated strains shows variance between strains, particularly in the IIIb (CXCR4 tropic) strain’s net electrostatics; the residue color scheme is based on the Clustal X algorithm.^5^ V3 loops of HIV_JR-CSF_, HIV_BaL_, HIV_2B4C_ (HIV_JR-FL_), HIV_YU2_, HIV_92UG037_, and HIV_IIIb_ have net charges of approximately +3, +3, +3, +3, +3, and +9 at physiological pH (7.4), respectively. These V3 loop residue numbers are used for the discussion of molecular docking results. N7 and N38 (not shown) are potentially glycosylated in native env.^6^

Amino acid sequences were obtained from the UniProt database.^7^ Each viral strain was independently homology modeled using either of two different template structures (Table S1) then subsequently energy minimized with the Amber12EHT force field in MOE.^4^

**Table A.** Comparison of strains used in homology modeling and MD simulations.

| **Strain** | **Source** | **PDB Template** | **Length** | **Classification**^†^ | **IC_50_ (ng/mL)**^‡^ | **Tropism**^§^ |
| --- | --- | --- | --- | --- | --- | --- |
| 2B4C (JR-FL) | Crystal structure | N/A | V1/V2 abridged | Tier II | Unknown | CCR5 |
| BaL | Homology Model | 2B4C | Full | Lab adapted | 230 | CCR5 |
| IIIb | Homology Model | 2B4C | Full | Lab adapted | 95 | CXCR4 |
| JR-CSF | Homology Model | 2B4C | Full | Tier II | 760 | CCR5 |
| 92UG037 | Homology Model | 2B4C | Full | Tier II | 750 | CCR5 |
| YU2 | Homology Model | 2QAD | Full | Tier II | Unknown | CCR5 |

^†^Classifications are based upon sensitivity to neutralizing antibodies.

^‡^Ligand anti-HIV experimental IC_50_ values from Savage *et al.*^8^

^§^Tropisim refers to the propensity to bind either the CCR5 or CXCR4 co-receptor.


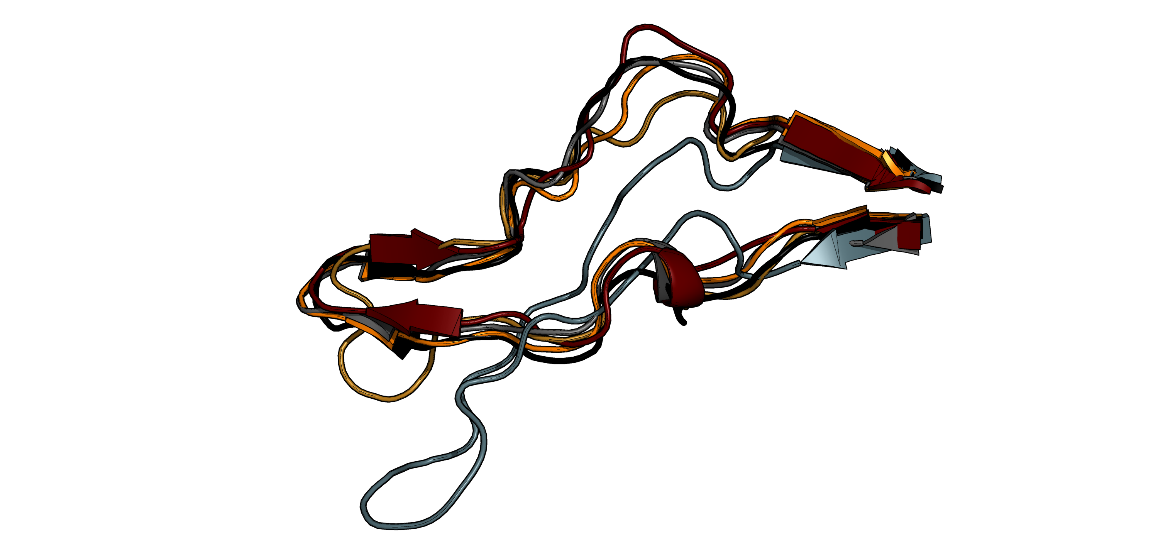


**Figure B.** Overlay of the V3 loop region of the homology models (orange, maroon, gold, gray), gp120_2B4C_ (black), and the V3 loop from gp120_YU2_ (2QAD, blue-gray). Homology models are biased towards the template structure (2B4C) in the V3 loop region as expected^9^, whereas the gp120_YU2_ structure from a separate crystal structure (2QAD) is positioned somewhat differently.

**Model Quality Analysis**

These six structures were analyzed with Ramachandran plots^10^, ANOLEA^11^, and QMEAN.^12^ In the Ramachandran plots, residues with outlier dihedral angles are marked with a red triangle or square and labeled (may require zooming in the web version of the supplemental information). Green in ANOLEA represents favorable (negative) residue energy scores, whereas red represents positive (unfavorable) energy scores. V3 loop residues are marked for clarity. High-resolution structures on average have a QMEAN score of zero; positive deviation from this score (right, blue) is increasingly favorable, whereas negative deviation (left, red) is increasingly unfavorable. Terms comprising the composite Z-score include C-β interaction potentials and all-atom interaction potentials (secondary structure dependency), torsion potential (backbone geometry and solvation potential).^12^

**gp120_2B4C_: JR-FL Strain**

V3

**Figure C.** Analysis metrics for the gp120_2B4C_ from the 2B4C^3^ crystal structure. The Ramachandran plot (left) shows favorable dihedral angles for all but six residues of the protein (299P and 323N lie in the V3 loop region). ANOLEA analysis (top right) shows unfavorable energetics in the V3 loop region in addition to a few other areas of the protein. The QMEAN score (bottom right) is fairly low, however, all metrics are with isolated gp120 without stabilizing peptides bound.

**gp120_92UG037_**

Uniprot Accession ID: Q0ED04

Sequence:

LWVTVYYGVPIWKDANTTLFCASDAKAYDTEVHNVWATHACVPTDPSPQELKMENVTEEFNMWKNNMVEQMHTDIISLWDQSLKPCVQLTPLCVTLDCSYNITNNITNSITNSSVNMREEIKNCSFNMTTELRDKNRKVYSLFYKLDVVQINNGNNSSNLYRLINCNTSALTQACPKVTFEPIPIHYCAPAGYAILKCNDKEFNGTGLCKNVSTVQCTHGIRPVVSTQLLLNGSLAEGKVMIRSENITNNVKNIIVQLNESVTINCTRPNNNTRRSVRIGPGQTFYATGDIIGDIRQAHCNVSGSQWNKTLHQVVEQLRKYWNNNTIIFNSSSGGDLEITTHSFNCGGEFFYCNTSGLFNSTWVNGTASIENGTITLPCRIKQIINMWQRVGQAIYAPPIQGVIRCVSNITGLILTRDGGGNSNENETFRPGGGDMRDNWRSELYKYKVVKIEPLGVAPTKARRRVVEREK

Homology Modeling Template (PDB ID): 2B4C

Analysis:

V3

**Figure D.** Analysis metrics for the gp120_92UG037_ homology model. Seven residues lie in outlier regions (left) on the Ramachandran plot (none in the V3 loop region). Additionally, some V3 loop residues have unfavorable ANOLEA scores (top right) and there is a very low QMEAN score (bottom right).

**gp120_BaL_**

Uniprot Accession ID: Q1XHI3

Sequence:

LWVTVYYGVPVWKEATTTLFCASDAKAYDTEVHNVWATHACVPTDPNPQEVKMENVTENFNMWKNNVVEQMHEDIISLWDQSLKPCVKLTPLCVTLNCTDLKNATNGNNTNTTSSSGGMMGGGEMKNCSFNITTNIRGKVQKEYALFYELDIVPIDNKIDSYRLISCNTSVITQACPKVSFEPIPIHYCAPAGFAILKCKDKKFNGKGPCSNVSTVQCTHGIRPVVSTQLLLNGSLAEEEVVIRSENFTNNAKIIVVQLNESVEINCTRPNNNTRKSIHIGPGRAFYTTGEIIGDIRQAHCNLSRAKWNDTLNKIVIKLREQFGNKTIVFKHSSGGDPEIVTHSFNCGGEFFYCNSTQLFNSTWNVTEESNNTVENNTITLPCRIKQIINMWQEVGRAMYAPPIRGQIRCSSNITGLLLTRDGGPEDDKTEVFRPGGGDMRDNWRSELYKYKVVKIEPLGVAPTKAKRRVVQREK

Homology Modeling Template (PDB ID): 2B4C

Analysis:

V3

**Figure E.** Analysis metrics for the gp120_BaL_ homology model. From the Ramachandran plot (left) nine residues, including two V3 loop residues (272N and 287Y) lie in outlier regions. The ANOLEA plot (top right) in the V3 loop region is unfavorable and the QMEAN score (bottom right) is low.

**gp120_IIIb_**

Uniprot Accession ID: A0A076PX70

Sequence:

KLWVTVYYGVPVWKEATTTLFCASDAKAYDTEVHNVWATHACVPTDPNPQEVVLVNVTENFNMWKNDMVEQMHEDIISLWDQSLKPCVKLTPLCVSLKCTDLKNDTNTNSSSGGMIMEKGEIKNCSFNISTSIRGKVQKEYAFFYKHDIIPIDNDTTSYTLTSCNTSVITQACPKVSFEPIPIHYCAPAGFAILKCNNKTFNGTGPCTNVSTVQCTHGIKPVVSTQLLLNGSLAEEEVVIRSANLTDNVKTIIVQLNQSVEINCTRPNNNTRKRIRIQRGPGRTFVTIGKIGNMRQAHCNISRAKWNNTLKQIASKLREQYGNNKTIIFKQSSGGDLEIVTHSFNCGGEFFYCNSTQLFNSTWFNSTWSTEGSNNTEGSDTITLPCRIKQIINMWQEVGKAMYAPPISGQIRCSSNITGLLLTRDGGNNNNGSEIFRPGGGDMRDNWRSELYKYKVVKIEPLGVAPTKAKRRVVQREK

Homology Modeling Template (PDB ID): 2B4C

Analysis:

V3

**Figure F.** Analysis metrics for the gp120_IIIb_ homology model. From the Ramachandran plot (left), six residues had unfavorable dihedral angles with one from the V3 loop region (266N). Despite the lowest sequence identity of gp120_IIIb_ to the template (2B4C) in the V3 loop region (Figure S1) the ANOLEA (top right) showed only a few negative energy residues in this region; however, the QMEAN score (bottom right) was low due to torsion angles.

**gp120_JR-CSF_**

Uniprot Accession ID: P20871

Sequence:

KLWVTVYYGVPVWKETTTTLFCASDAKAYDTEVHNVWATHACVPTDPNPQEVVLENVTEDFNMWKNNMVEQMQEDVINLWDQSLKPCVKLTPLCVTLNCKDVNATNTTSSSEGMMERGEIKNCSFNITKSIRDKVQKEYALFYKLDVVPIDNKNNTKYRLISCNTSVITQACPKVSFEPIPIHYCAPAGFAILKCNNKTFNGKGQCKNVSTVQCTHGIRPVVSTQLLLNGSLAEEKVVIRSDNFTDNAKTIIVQLNESVKINCTRPSNNTRKSIHIGPGRAFYTTGEIIGDIRQAHCNISRAQWNNTLKQIVEKLREQFNNKTIVFTHSSGGDPEIVMHSFNCGGEFFYCNSTQLFNSTWNDTEKSSGTEGNDTIILPCRIKQIINMWQEVGKAMYAPPIKGQIRCSSNITGLLLTRDGGKNESEIEIFRPGGGDMRDNWRSELYKYKVVKIEPLGVAPTKAKRRVVQREK

Homology Modeling Template (PDB ID): 2B4C

Analysis:

V3

**Figure G.** Analysis metrics for the gp120_JR-CSF_ homology model. The Ramachandran plot (left) shows that one (283Y) of seven unfavorable residues lies in the V3 loop region. The ANOLEA plot (top left) shows an overall stable gp120 with unfavorable V3 loop energetics. The QMEAN score (bottom left) is low.

**gp120_YU2_**

Uniprot Accession ID: P35961

Sequence:

QLWVTVYYGVPVWKEATTTLFCASDAKAYDTEVHNVWATHACVPTDPNPQEVKLENVTENFNMWKNNMVEQMHEDIISLWDQSLKPCVKLTPLCVTLNCTDLRNATNTTSSSWETMEKGEIKNCSFNITTSIRDKVQKEYALFYNLDVVPIDNASYRLISCNTSVITQACPKVSFEPIPIHYCAPAGFAILKCNDKKFNGTGPCTNVSTVQCTHGIRPVVSTQLLLNGSLAEEEIVIRSENFTNNAKTIIVQLNESVVINCTRPNNNTRKSINIGPGRALYTTGEIIGDIRQAHCNLSKTQWENTLEQIAIKLKEQFGNNKTIIFNPSSGGDPEIVTHSFNCGGEFFYCNSTQLFTWNDTRKLNNTGRNITLPCRIKQIINMWQEVGKAMYAPPIRGQIRCSSNITGLLLTRDGGKDTNGTEIFRPGGGDMRDNWRSELYKYKVVKIEPLGVAPTKAKRRVVQREKR

Homology Modeling Template (PDB ID): 2QAD

Analysis:

V3

**Figure H.** Analysis metrics for the gp120_YU2_ homology model (V1/V2 loops were built into the 2QAD crystal structure). From the Ramachandran plot, only four residues have unfavorable dihedral angles, none of which lie in the V3 loop region. The ANOLEA (top right) shows unfavorable residue positioning in the V1/V2 loop region (mutated protein did not express these variable regions) and the V3 loop region; the QMEAN value (bottom right) was very low.

**Molecular Dynamics**

Molecular dynamics simulations were run until all structures had 100 ns of convergence with either of the two force fields, CHARMM36 or Amber99SB-ILDN. Several metrics, including block averaging and RMSD, were used to judge structural convergence over the final 100 ns. Structural fluctuation was analyzed with root-mean-squared-fluctuation (RMSF) and principal component analysis.

**Simulation Conditions**

**Table B.** Molecular dynamics cubic box and solvation conditions.

| **Strain** | **Net Charge (+)** | **Cl- Ions** | **Na+ Ions** | **# Atoms** | **Box Side Length (Å)** |
| --- | --- | --- | --- | --- | --- |
| 2B4C | 1 | 138 | 137 | 151979 | 11.49764 |
| 92UG037 | 6 | 137 | 131 | 139352 | 11.31123 |
| BaL | 4 | 139 | 135 | 151074 | 11.44501 |
| IIIb | 10 | 145 | 135 | 151140 | 11.44138 |
| JR-CSF | 7 | 141 | 134 | 149130 | 11.40365 |
| YU2 | 6 | 134 | 128 | 139499 | 11.22564 |

**Molecular Docking
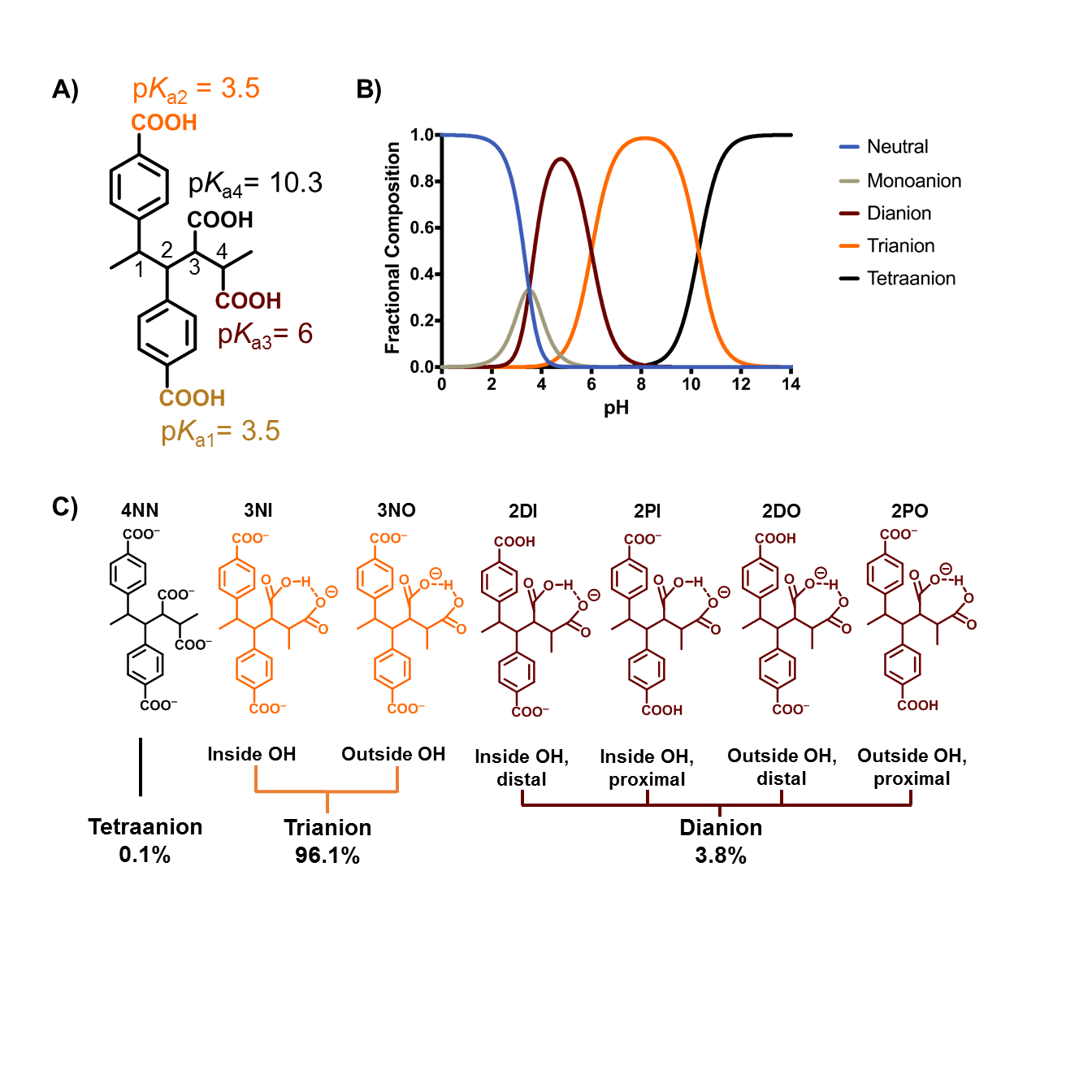
**

**Figure I.** (A) Repeat unit of the ligand with chirality centers and carboxyl p*K*_a_ values obtained from Figure 6 of Li *et al*.^13^ (B) Ligand pH vs. ionic species (microspecies) distribution, calculated using the Henderson-Hasselbach equation assuming the ligand behaves as a polyprotic acid. (C) Potential microspecies present at pH 7.4, microspecies nomenclature, and abbreviations used in subsequent tables.

Molecular docking was first conducted on gp120 structures (homology models and the 2B4C crystal structure) prior to MD simulations. Autodock Tools^14^ was used for file preparation and box generation (Tables S3 and S4); docking was conducted with Autodock Vina.^15^ All of the gp120s were aligned to gp120_2B4C_ with PyMOL^16^, and a box that encompassed all proteins (“Largebox”) was used for V3 loop binding verification (Table S5).

**Table C.** Box coordinates for the docking to gp120 models prior to MD.

| **Protein** | **X center** | **Y center** | **Z center** | **X size (Å)** | **Y size (Å)** | **Z size (Å)** |
| --- | --- | --- | --- | --- | --- | --- |
| **Largebox**^†^ | 94.625 | -124.912 | 138.136 | 79 | 84 | 98 |
| **gp120_2B4C_** | 92.195 | -138.227 | 166.06 | 26 | 33 | 40 |
| **gp120_92UG037_** | 95.043 | -138.227 | 165.307 | 26 | 33 | 40 |
| **gp120_BaL_** | 92.199 | -138.227 | 166.06 | 26 | 33 | 40 |
| **gp120_IIIb_** | 90.136 | -138.227 | 166.06 | 26 | 33 | 40 |
| **gp120_JR-CSF_** | 92.194 | -138.227 | 166.06 | 26 | 33 | 40 |
| **gp120_YU2_** | 92.195 | -138.227 | 168.6 | 26 | 33 | 40 |

^†^Box that encompasses the entire protein across all aligned proteins

**Table D.** Fraction of binding poses in the V3 loop regions for the RRRR ligand for the 20 poses that were generated in Vina with the Largebox.

|  | **gp120_2B4C_** | **gp120_YU2_** | **gp120_92UG037_** | **gp120_BaL_** |  | **gp120_IIIb_** | **gp120_JR-CSF_** |
| --- | --- | --- | --- | --- | --- | --- | --- |
| **RRRR4NN** | 0.15 | 0.1 | 0.4 | 0.25 |  | 0.1 | 0.3 |
| **RRRR3NI** | 0.35 | 0 | 0.6 | 0.35 |  | 0.1 | 0 |
| **RRRR3NO** | 0.45 | 0 | 0.35 | 0.2 |  | 0.05 | 0.1 |
| **RRRR2PI** | 0.25 | 0.05 | 0.3 | 0.5 |  | 0.2 | 0.2 |
| **RRRR2PO** | 0.6 | 0.1 | 0.5 | 0.4 |  | 0.2 | 0 |
| **RRRR2DI** | 0.35 | 0 | 0.35 | 0.4 |  | 0.2 | 0.15 |
| **RRRR2DO** | 0.4 | 0 | 0.5 | 0.4 |  | 0.5 | 0.15 |

**Table E.** Box coordinates for the docking done to gp120 models following MD simulations with the CHARMM36 force field and clustering over 500-600 ns (Figure S40).

| **Simulation** | **Replicate** | **X Center** | **Y Center** | **Z Center** | **X Size (Å)** | **Y Size (Å)** | **Z Size (Å)** |
| --- | --- | --- | --- | --- | --- | --- | --- |
| **Largebox**^†^ | N/A | 91.722 | -127.54 | 131.93 | 102 | 112 | 86 |
| **gp120_2B4C_** | 1 | 91.555 | -127.54 | 149.463 | 20 | 31 | 22 |
| **gp120_2B4C_** | 2 | 90.645 | -154.439 | 154.439 | 22 | 24 | 32 |
| **gp120_2B4C_** | 3 | 82.536 | -165.201 | 117.483 | 22 | 30 | 26 |
| **gp120_92UG037_** | 1 | 83.002 | -145.357 | 121.788 | 28 | 24 | 32 |
| **gp120_92UG037_** | 2 | 96.138 | -157.160 | 136.34 | 24 | 16 | 36 |
| **gp120_92UG037_** | 3 | 83.754 | -144.176 | 125.576 | 22 | 26 | 34 |
| **gp120_BaL_** | 1 | 81.006 | -146.821 | 123.922 | 26 | 32 | 26 |
| **gp120_BaL_** | 2 | 86.623 | -161.121 | 113.321 | 24 | 24 | 26 |
| **gp120_BaL_** | 3 | 79.538 | -153.626 | 125.704 | 28 | 22 | 18 |
| **gp120_IIIb_** | 1 | 80.337 | -157.223 | 116.218 | 32 | 26 | 24 |
| **gp120_IIIb_** | 2 | 76.885 | -144.308 | 112.956 | 30 | 28 | 18 |
| **gp120_IIIb_** | 3 | 89.078 | -159.253 | 119.097 | 30 | 30 | 28 |
| **gp120_JR-CSF_** | 1 | 81.699 | -146.234 | 121.386 | 24 | 28 | 26 |
| **gp120_JR-CSF_** | 2 | 95.563 | -154.764 | 121.386 | 32 | 20 | 22 |
| **gp120_JR-CSF_** | 3 | 90.849 | -146.669 | 112.946 | 20 | 30 | 28 |
| **gp120_YU2_** | 1 | 87.496 | -135.542 | 160.588 | 30 | 25 | 27 |
| **gp120_YU2_** | 2 | 93.978 | -133.442 | 159.301 | 33 | 26 | 25 |
| **gp120_YU2_** | 3 | 91.636 | -141.890 | 156.995 | 28 | 28 | 25 |

^†^Box that encompasses the entire protein across all strains

**
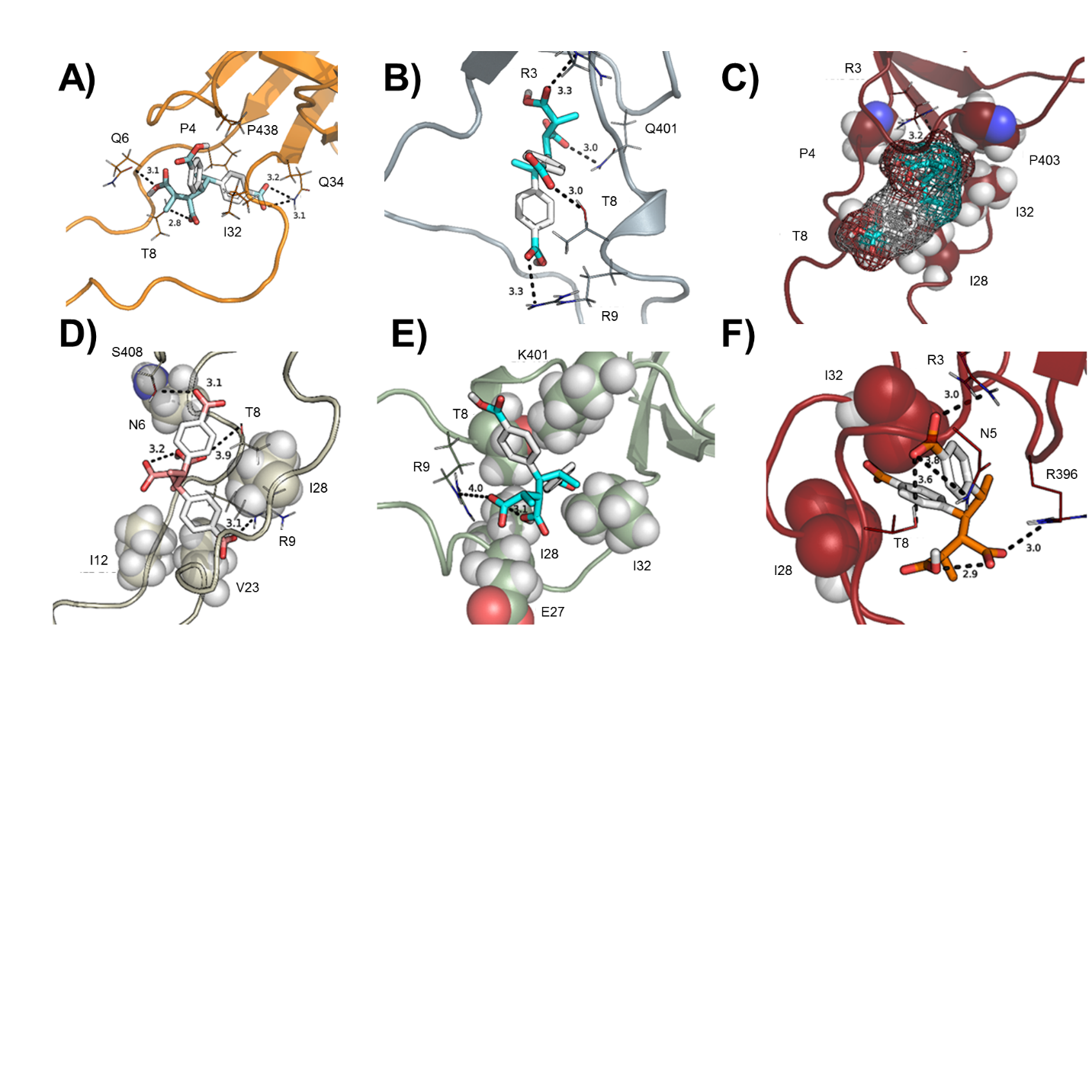
**

**Figure J.** Examples of binding of a ligand to gp120 from various strains of HIV. **A)** gp120_2B4C_ with SRRS2PO, **B)** gp120_92UG037_ with RSSS2DO, **C)** gp120_BaL_ with SRSR2DI, **D)** gp120_IIIb_ with SRSR2DI, **E)** gp120_JR-CSF_ with SSSR2PI, and **F)** gp120_YU2_ with RSSR3NO. Ligands are represented in stick and proteins are represented in cartoon, with the surface representation of hydrophobic residues and line representation of gp120 amino acids that take part in hydrogen bonding being shown. V3 loop residues are numbered according to aligned loop sequences (Figure S1).

**
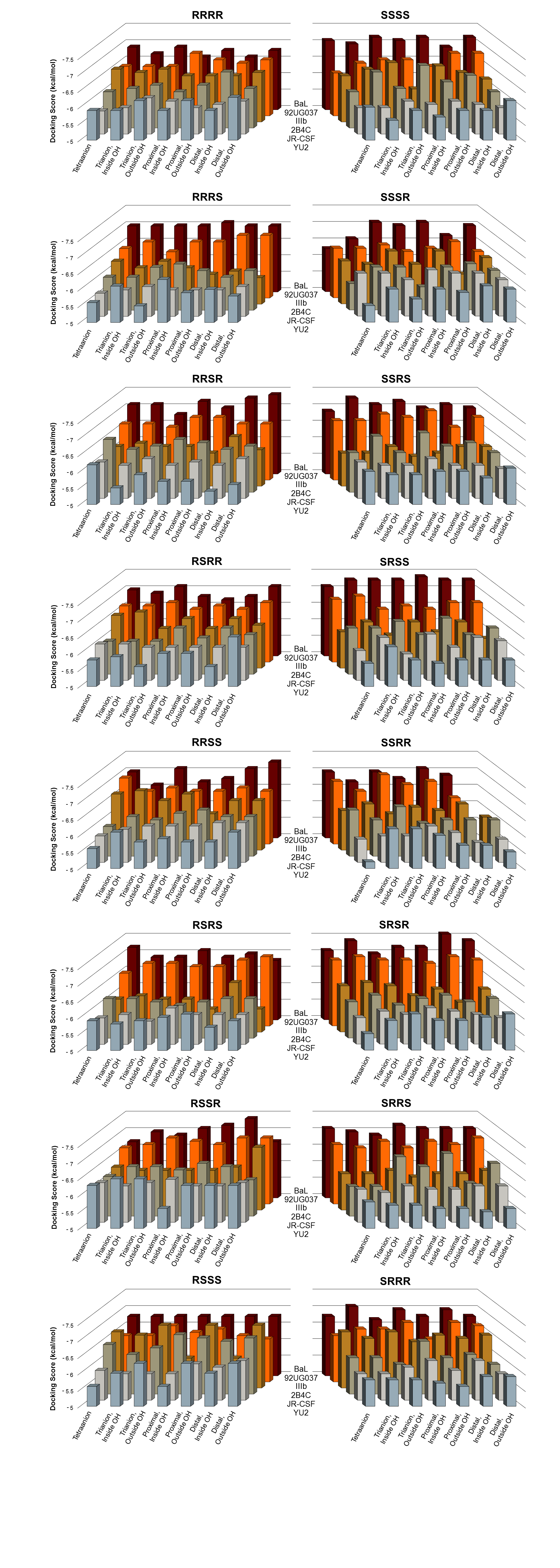
**

**
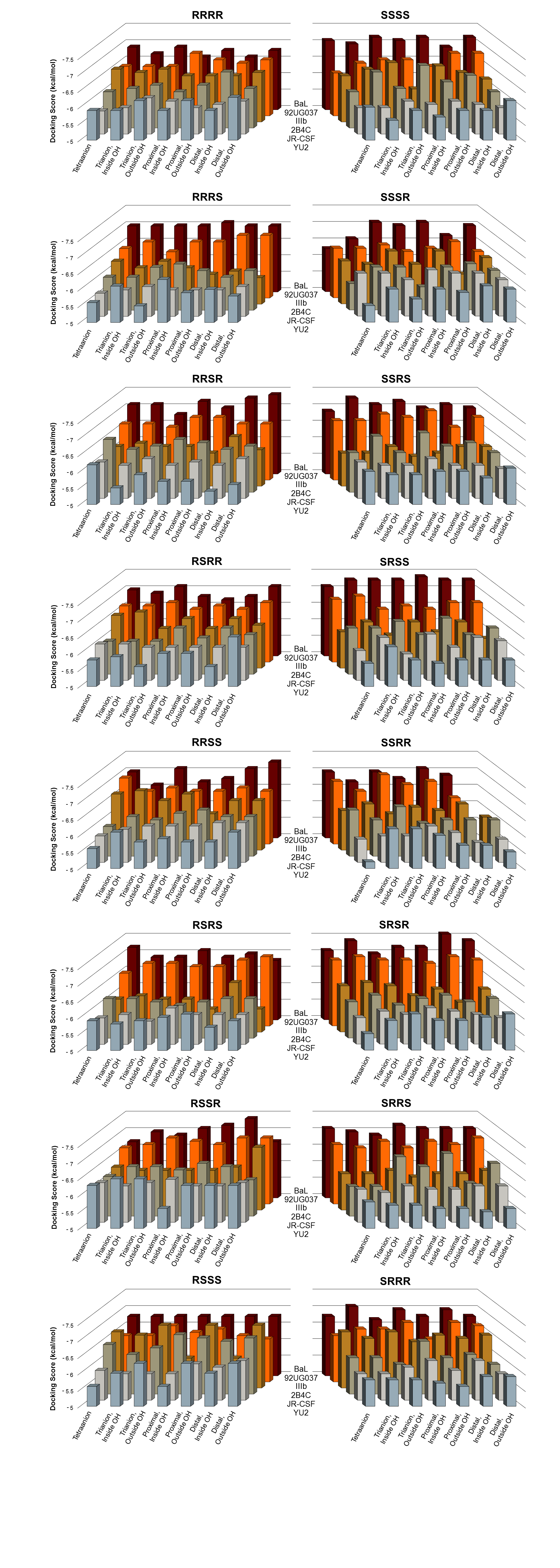
**

**Figure K.** Vina docking scores of ligands binding to the V3 loops of each gp120 (gp120_IIIb_, gp120_BaL_, gp120_92UG037_, gp120_JR-CSF_, gp120_YU2_, and gp120_2B4C_) grouped by ligand stereochemistry and protonation state (Figure S9) prior to MD simulations.

**Ligand Fingerprint Analysis: Models**

**gp120_2B4C_ prior to MD Simulation**

**
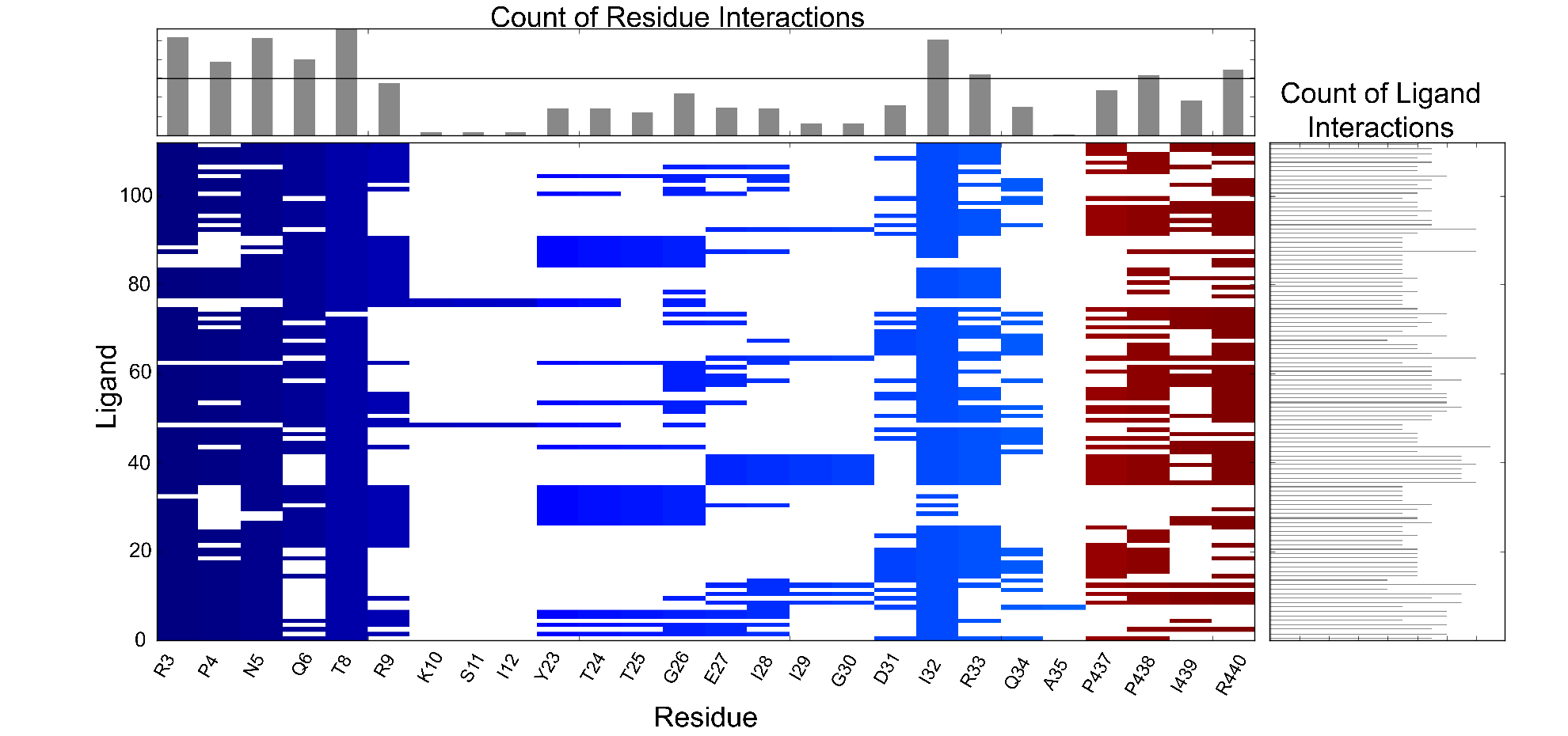
**

**Figure L.** Ligand fingerprinting of gp120_2B4C_ docking results prior to MD simulation. Residues in the V3 loop (R3–A35) are renumbered according to Figure S1, and a 50% threshold of interactions across poses is marked with a black line in the upper part of the figure. R3 and R9 form hydrogen bonds and have hydrophilic interactions with protonated carboxylic acid groups on ligands. There are hydrophobic contacts with P4, I32, and P438 while there are hydrophilic contacts with Q6 and T8. Backbone interactions include N5 and R440.

**gp120_92UG037_ prior to MD Simulation**


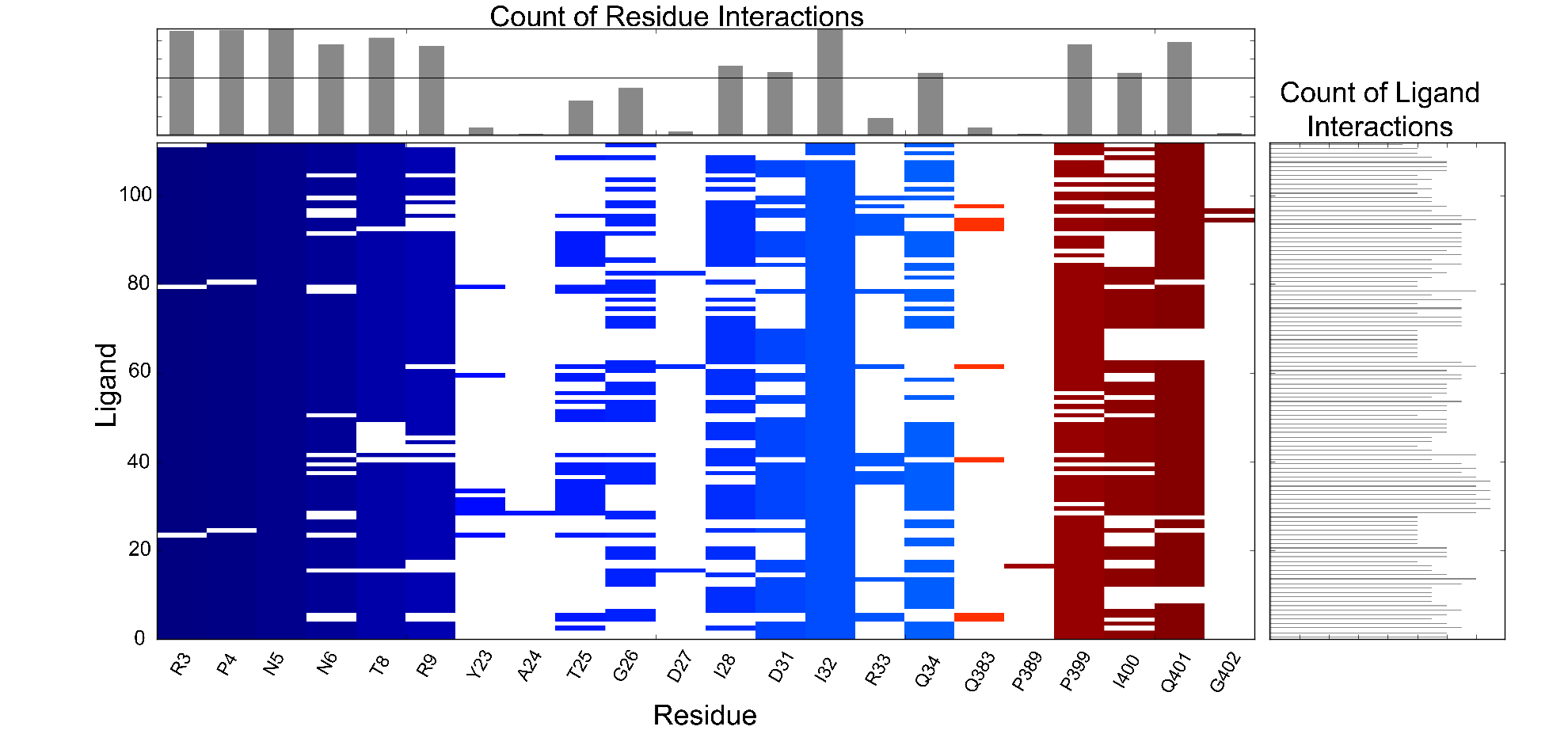


**Figure M.** Ligand fingerprinting of gp120_92UG037_ docking results prior to MD simulation. Residues in the V3 loop (R3–Q34) are renumbered according to Figure S1, and a 50% threshold of interactions across poses is marked with a black line in the upper part of the figure. The ligands form hydrophilic interactions with R3, T8, R9, Q34, and Q401, hydrophobic interactions with P4, I28, I32, and P399, and backbone interactions with N5, D31, and I400.

**gp120_BaL_ prior to MD Simulations
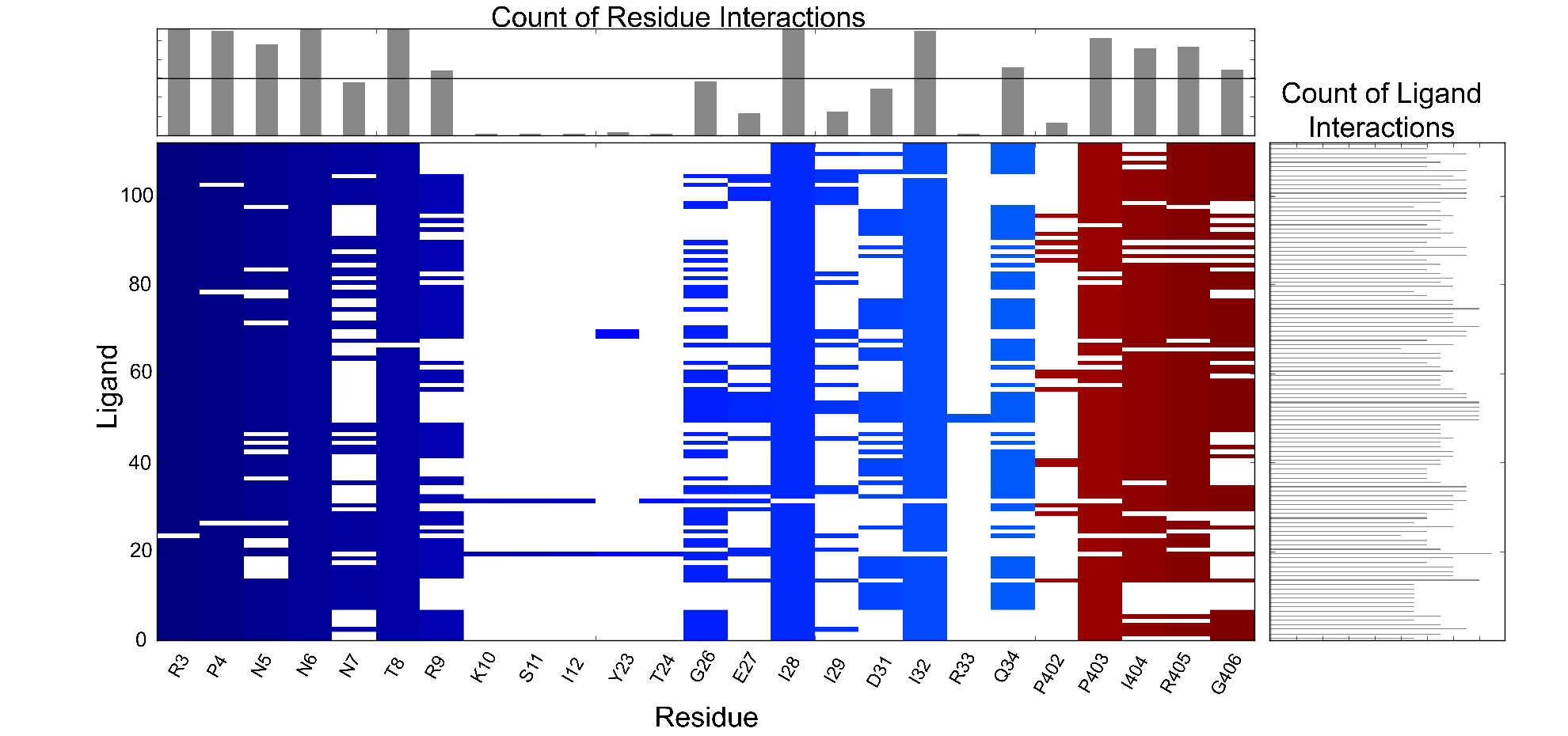
**

**Figure N.** Ligand fingerprinting of gp120_BaL_ docking results prior to MD simulation. Residues in the V3 loop (R3–Q34) are renumbered according to Figure S1, and a 50% threshold of interactions across poses is marked with a black line in the upper part of the figure. The ligands participate in hydrophilic interactions with R3, N6, T8, R9, Q34, and R405, whereas hydrophobic interactions include P4, I28, I32, and P403. There are backbone interactions with N5, G26, I404 and G406.

**gp120_IIIb_ prior to MD Simulation**


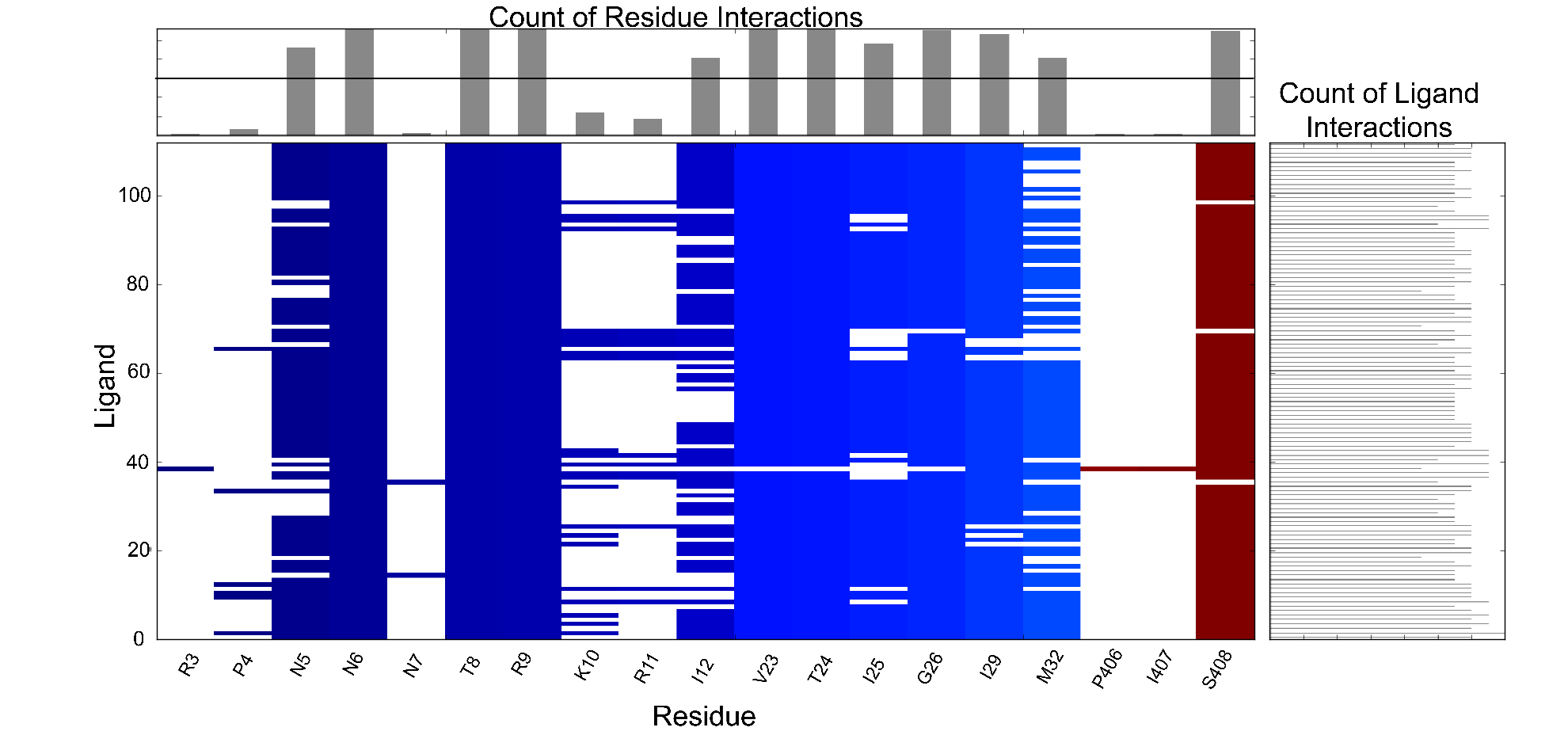


**Figure O.** Ligand fingerprinting of gp120_IIIb_ docking results prior to MD simulation. Residues in the V3 loop (R3–M32) are renumbered according to Figure S1, and a 50% threshold of interactions across poses is marked with a black line in the upper part of the figure. The ligands participate in hydrogen bonding interactions with T8 and R9, hydrophilic interactions with N6 and S408, and hydrophobic interactions with I12, V23, I29, M32. There are backbone interactions with N5, T24, I25, and G26.

**gp120_JR-CSF_ prior to MD Simulations**


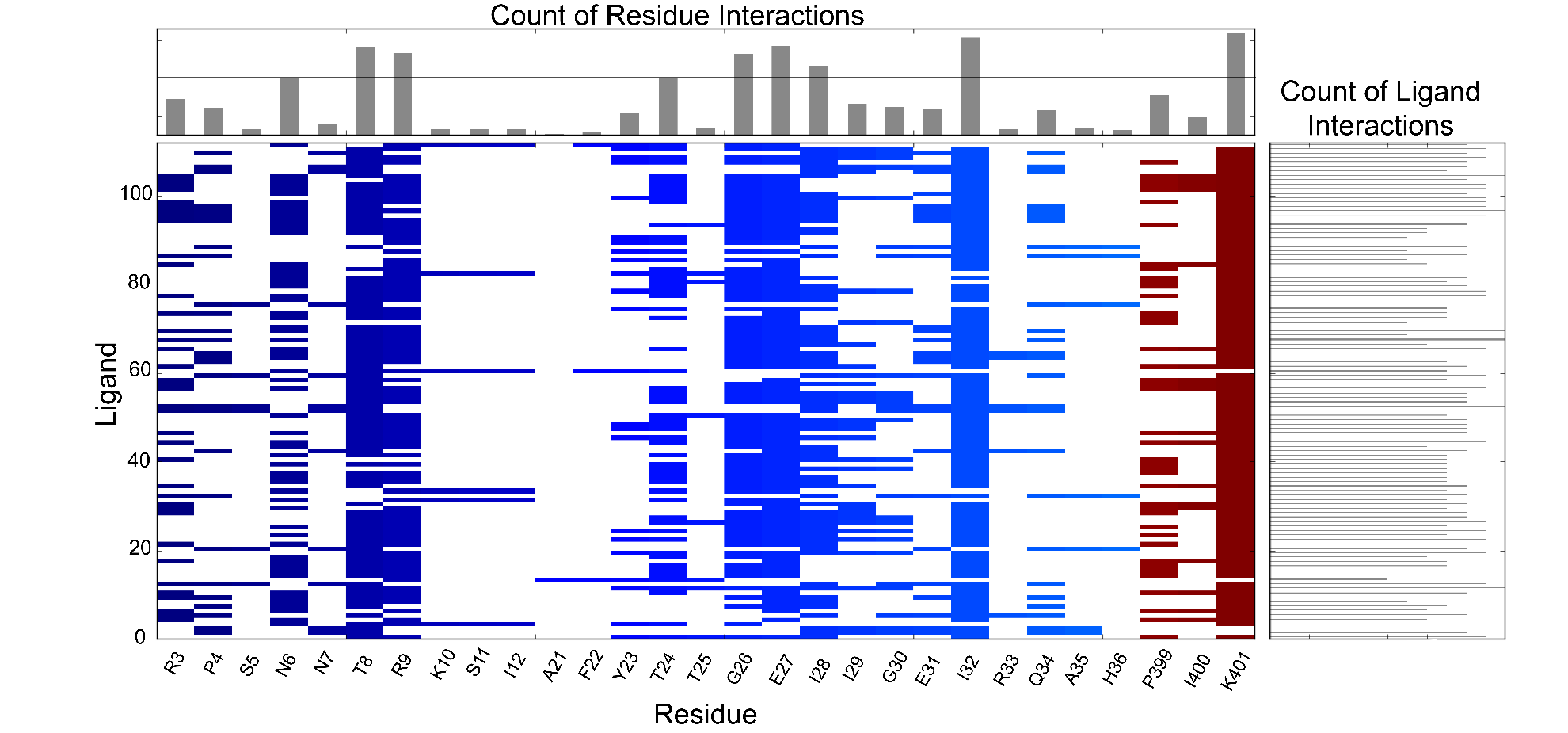


**Figure P.** Ligand fingerprinting of gp120_JR-CSF_ docking results prior to MD simulation. Residues in the V3 loop (R3–H36) are renumbered according to Figure S1, and a 50% threshold of interactions across poses is marked with a black line in the upper part of the figure. The ligands participate in hydrophilic interactions with N6, T8, R9, E27, and K401, hydrophobic interactions with I28 and I32, and backbone interactions with T24 and G26.

**gp120_YU2_ prior to MD Simulations**


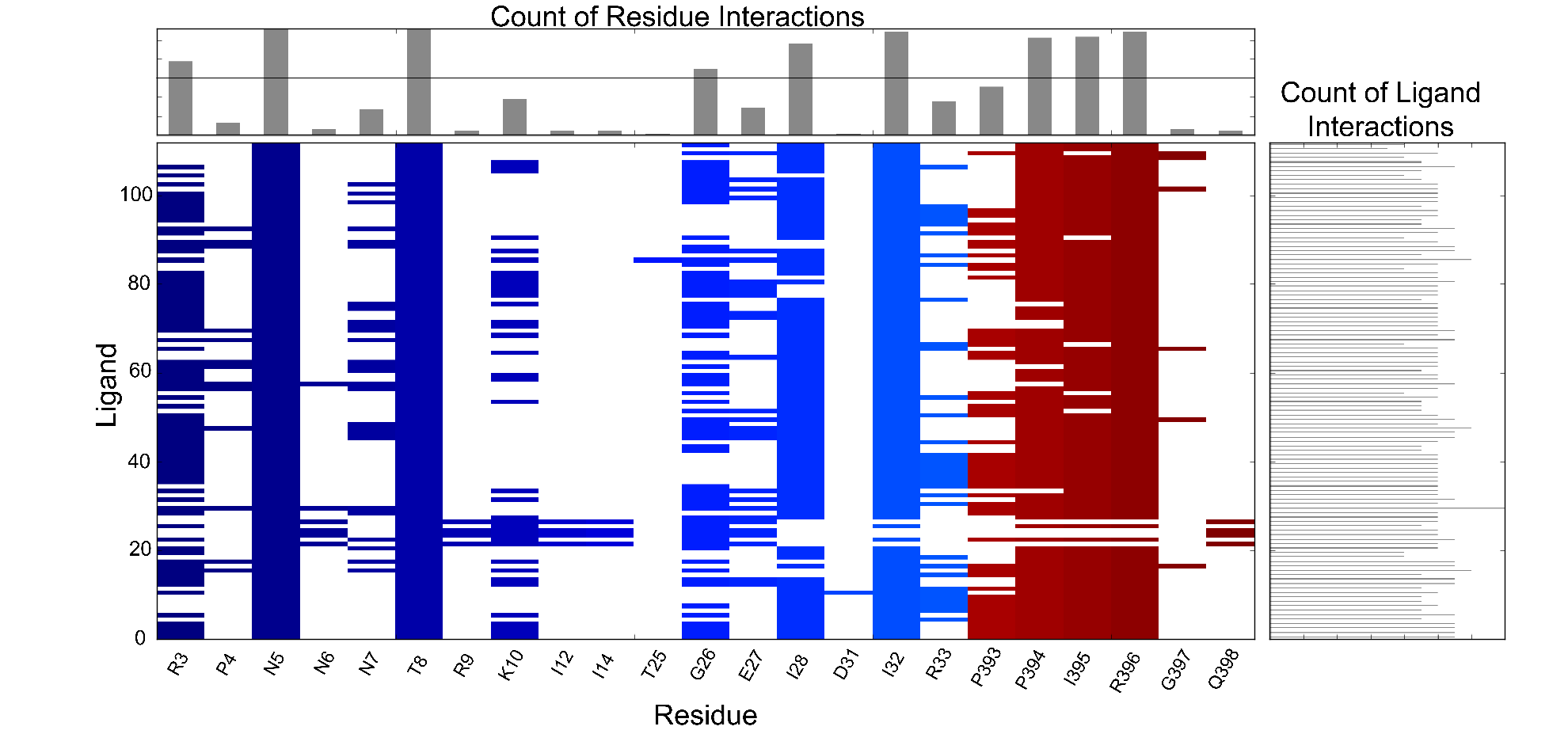


**Figure Q.** Ligand fingerprinting of gp120_YU2_ docking results prior to MD simulation. Residues in the V3 loop (R3–R33) are renumbered according to Figure S1, and a 50% threshold of interactions across poses is marked with a black line in the upper part of the figure. The ligands participate in a hydrogen bonding interactions with R33 (low population) and R396, hydrophilic interactions with R3, N5, and T8, hydrophobic interaction with I28, I32, and P393, and backbone interactions with G36, P394, and I395.

**Ligand Fingerprint Analysis: Ensembles from MD Simulations**

**gp120_2B4C_: Representative MD Cluster, Charmm Replicate 1 (Figure S40)**


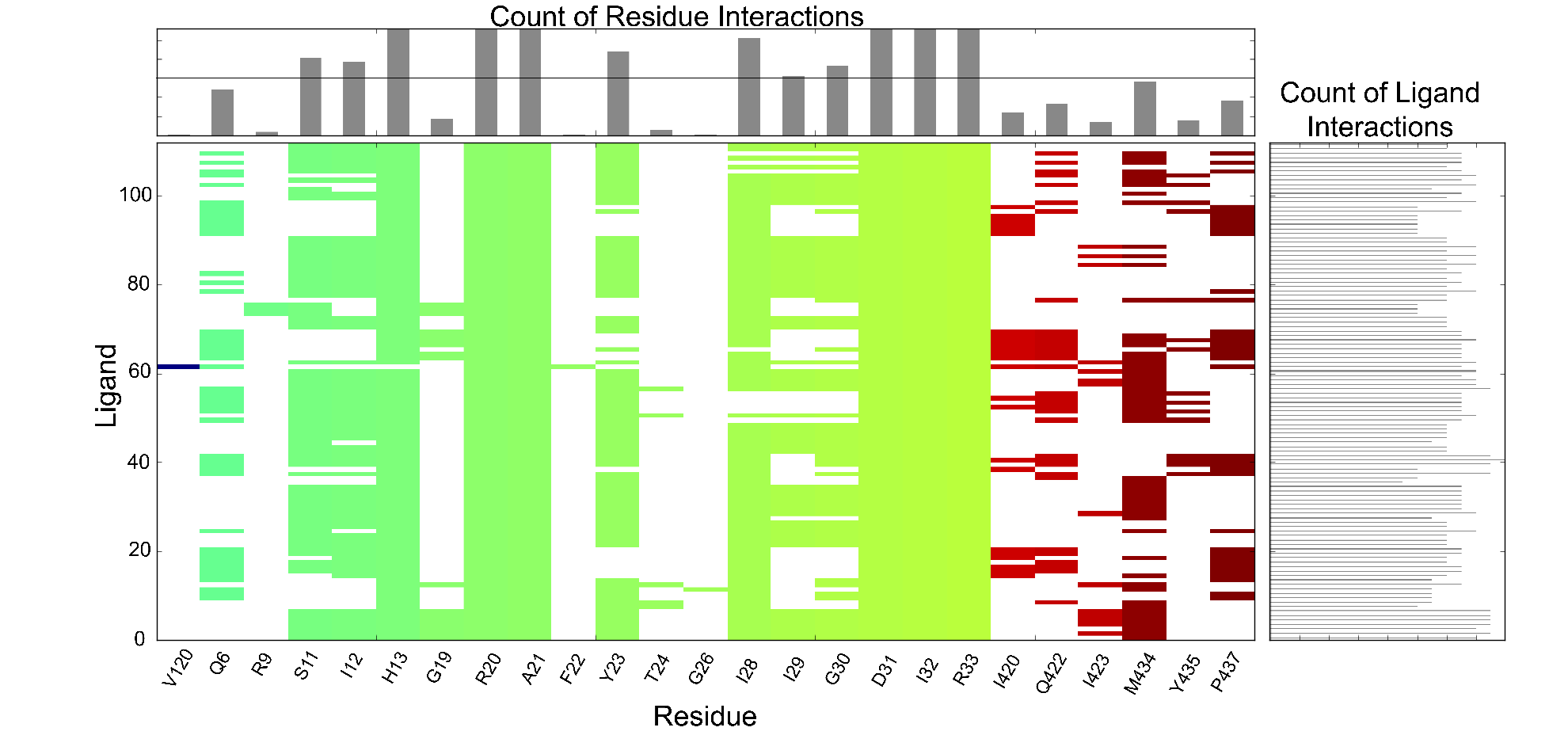


**Figure R.** Ligand fingerprinting of docking results from replicate 1 of gp120_2B4C_ following MD simulation. Residues in the V3 loop (Q6–R33) are renumbered according to Figure S1, and a 50% threshold of interactions across poses is marked with a black line in the upper part of the figure.

**gp120_2B4C_: Representative MD Cluster, Charmm Replicate 2 (Figure S40)**


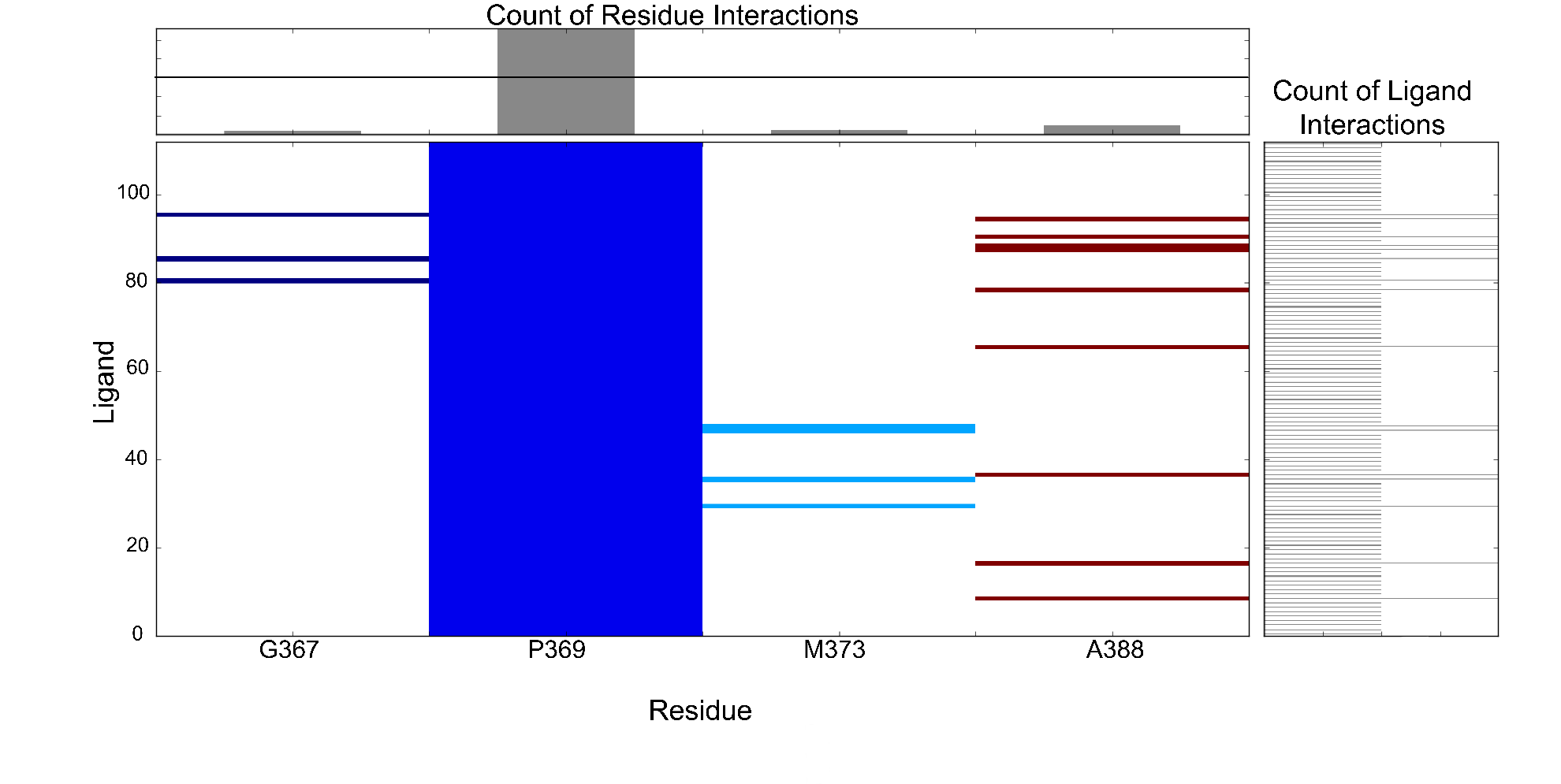


**Figure S.** Ligand fingerprinting of docking results from replicate 2 of gp120_2B4C_ following MD simulation. A 50% threshold of interactions across poses is marked with a black line in the upper part of the figure. There are hydrophobic interactions with P369, and the ligand does not interact with residues on the V3 loop. Folding of the V3 loop in this replicate likely explains the lack of interactions with ligands (Figure S40).

**gp120_2B4C_: Representative MD Cluster, Charmm Replicate 3 (Figure S40)**


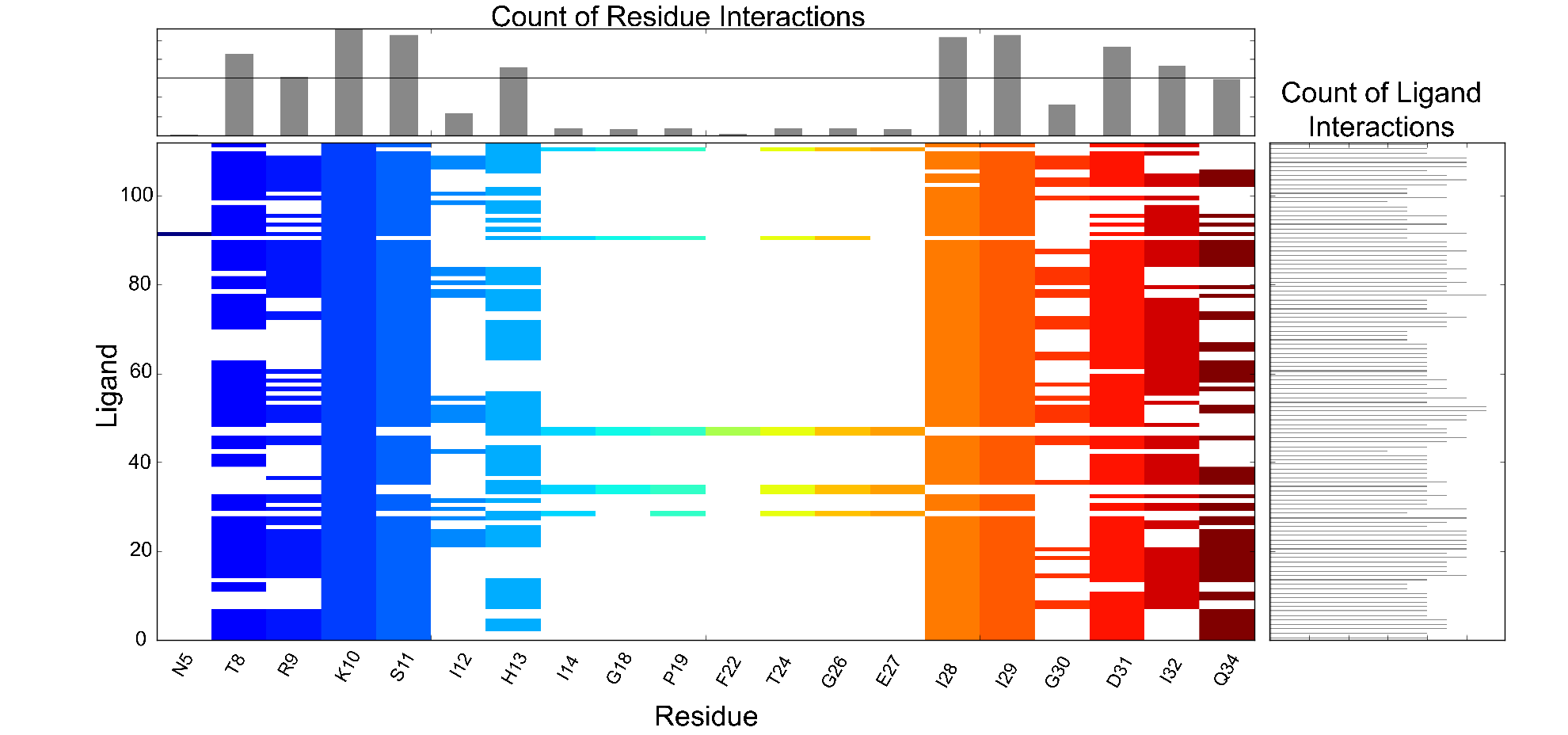


**Figure T.** Ligand fingerprinting of docking results from replicate 3 of gp120_2B4C_ following MD simulation. Residues in the V3 loop (N5–Q34) are renumbered according to Figure S1, and a 50% threshold of interactions across poses is marked with a black line in the upper part of the figure.

**gp120_92UG037_: Representative MD Cluster, Charmm Replicate 1 (Figure S40)**


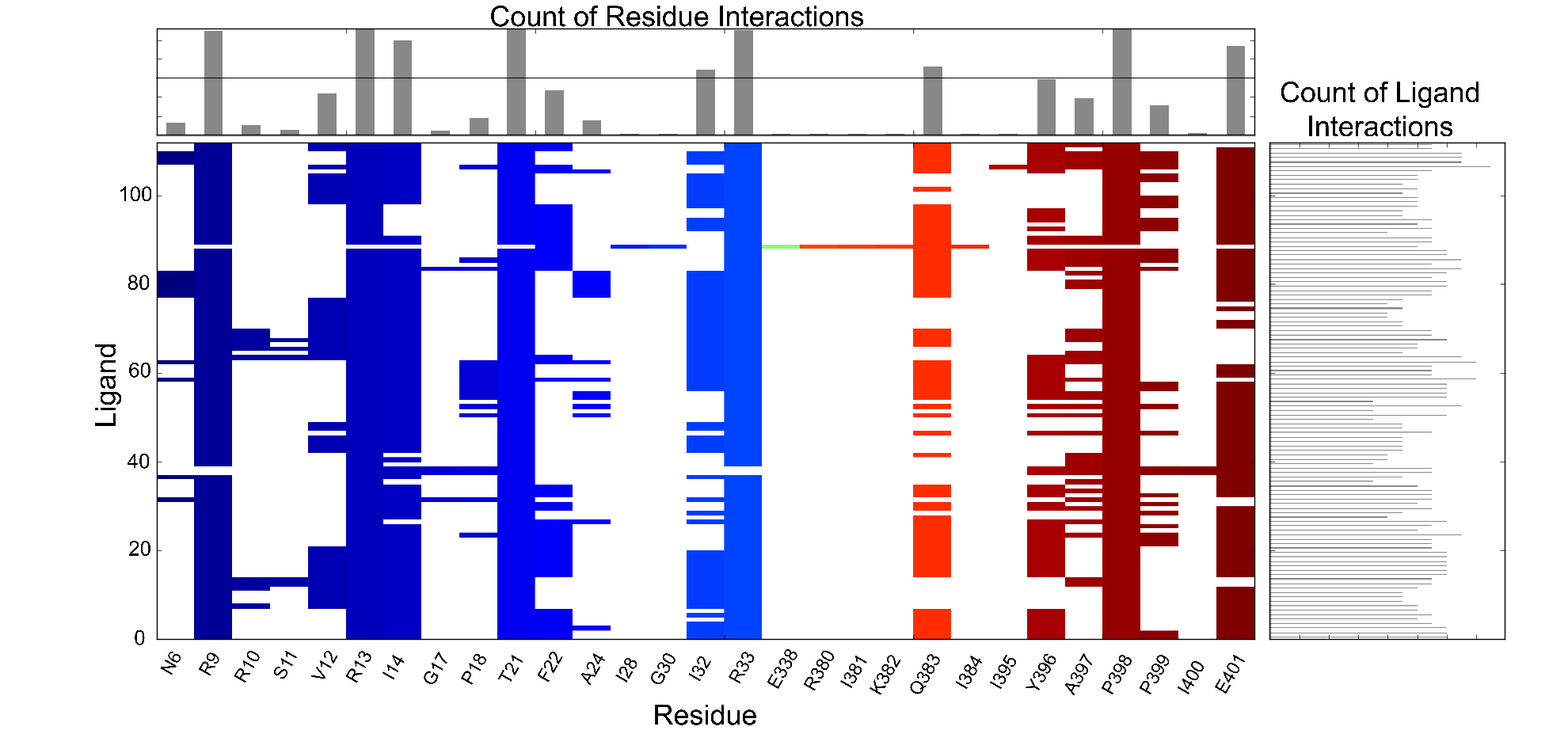


**Figure U.** Ligand fingerprinting of docking results from replicate 1 of gp120_92UG037_ following MD simulation. Residues in the V3 loop (N6–R33) are renumbered according to Figure S1, and a 50% threshold of interactions across poses is marked with a black line in the upper part of the figure.

**gp120_92UG037_: Representative MD Cluster, Charmm Replicate 2 (Figure S40)**


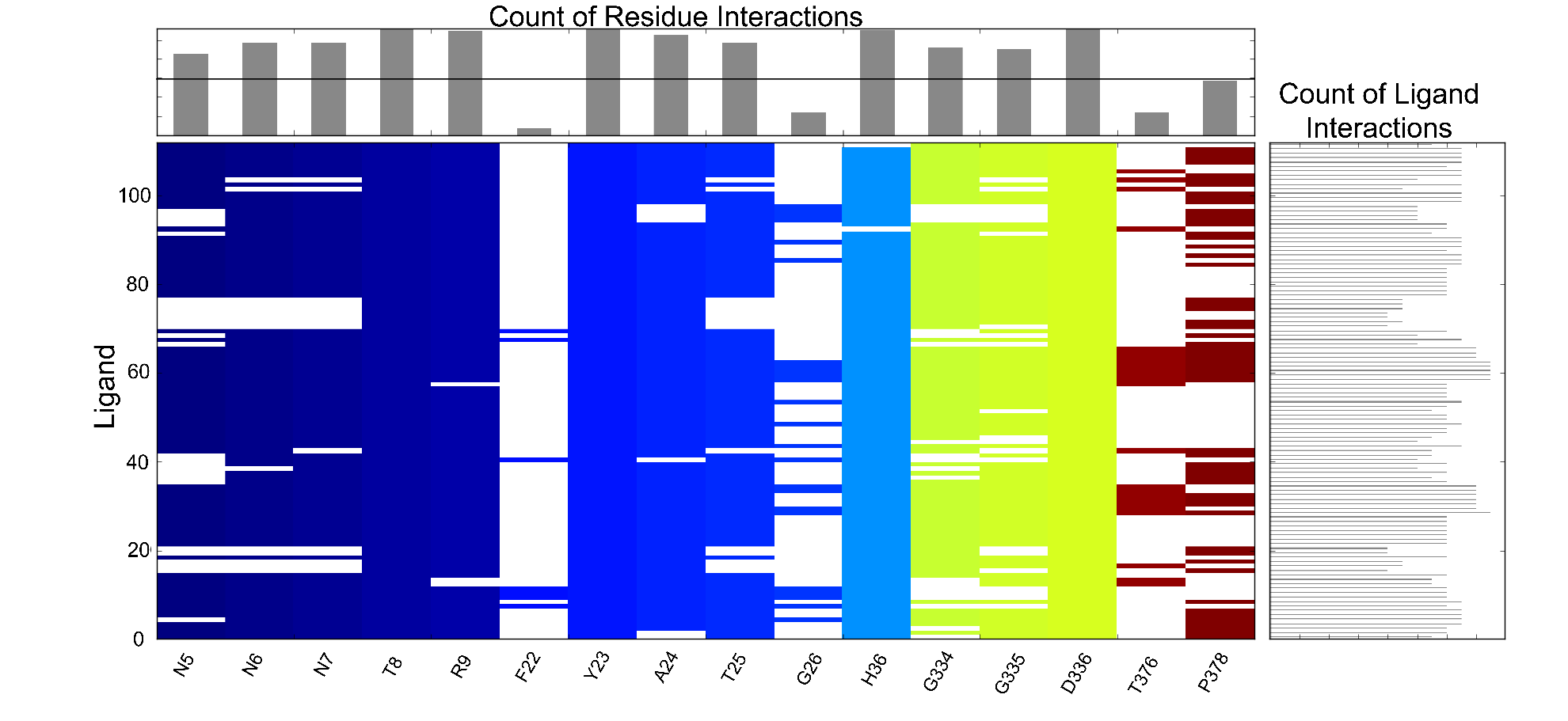


**Figure V.** Ligand fingerprinting of docking results from replicate 2 of gp120_92UG037_ following MD simulation. Residues in the V3 loop (N5–H36) are renumbered according to Figure S1, and a 50% threshold of interactions across poses is marked with a black line.

**gp120_92UG037_: Representative MD Cluster, Charmm Replicate 3 (Figure S40)**


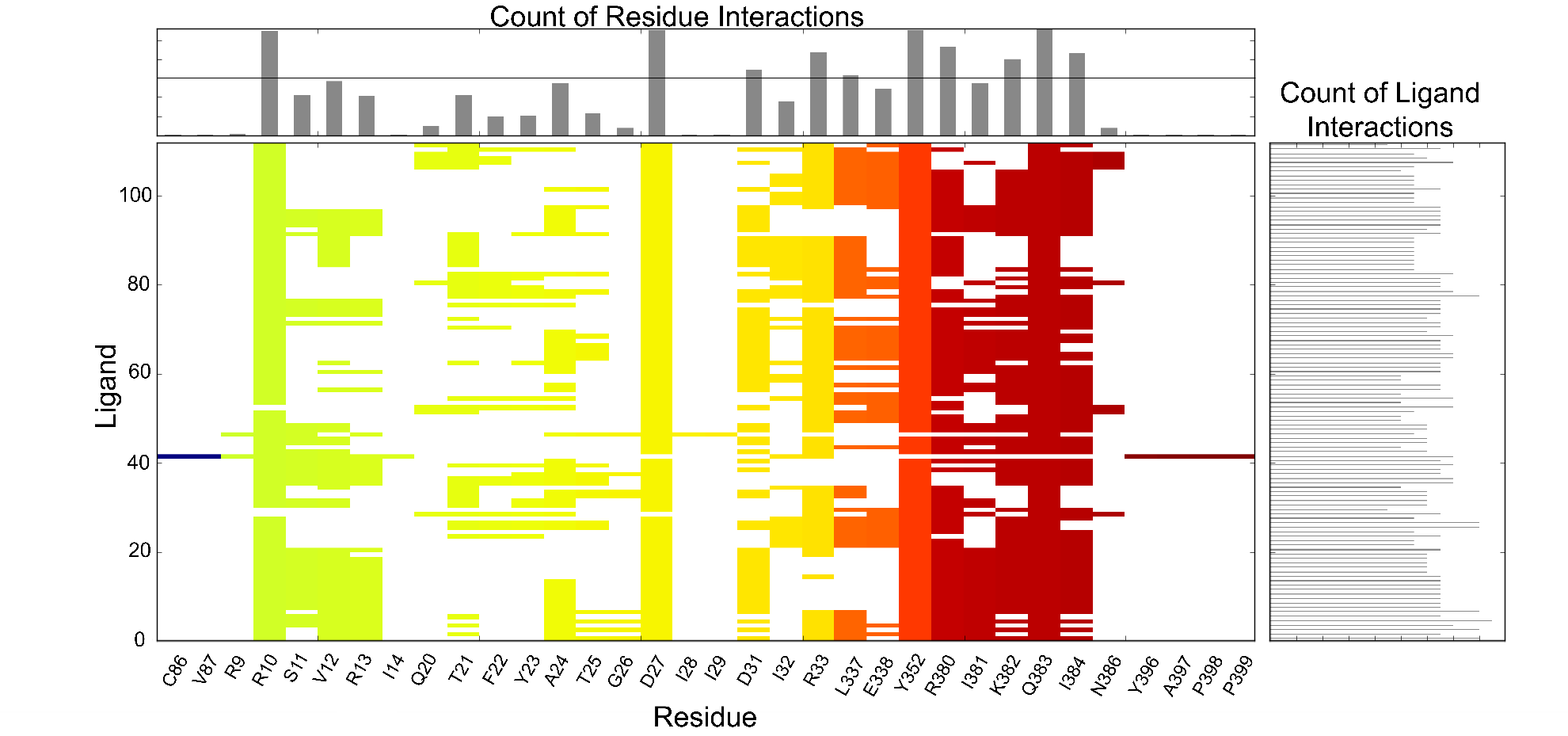


**Figure W.** Ligand fingerprinting of docking results from replicate 3 of gp120_92UG037_ following MD simulation. Residues in the V3 loop (R9–R33) are renumbered according to Figure S1, and a 50% threshold of interactions across poses is marked with a black line in the upper part of the figure.

**gp120_BaL_: Representative MD Cluster, Charmm Replicate 1 (Figure S40)**


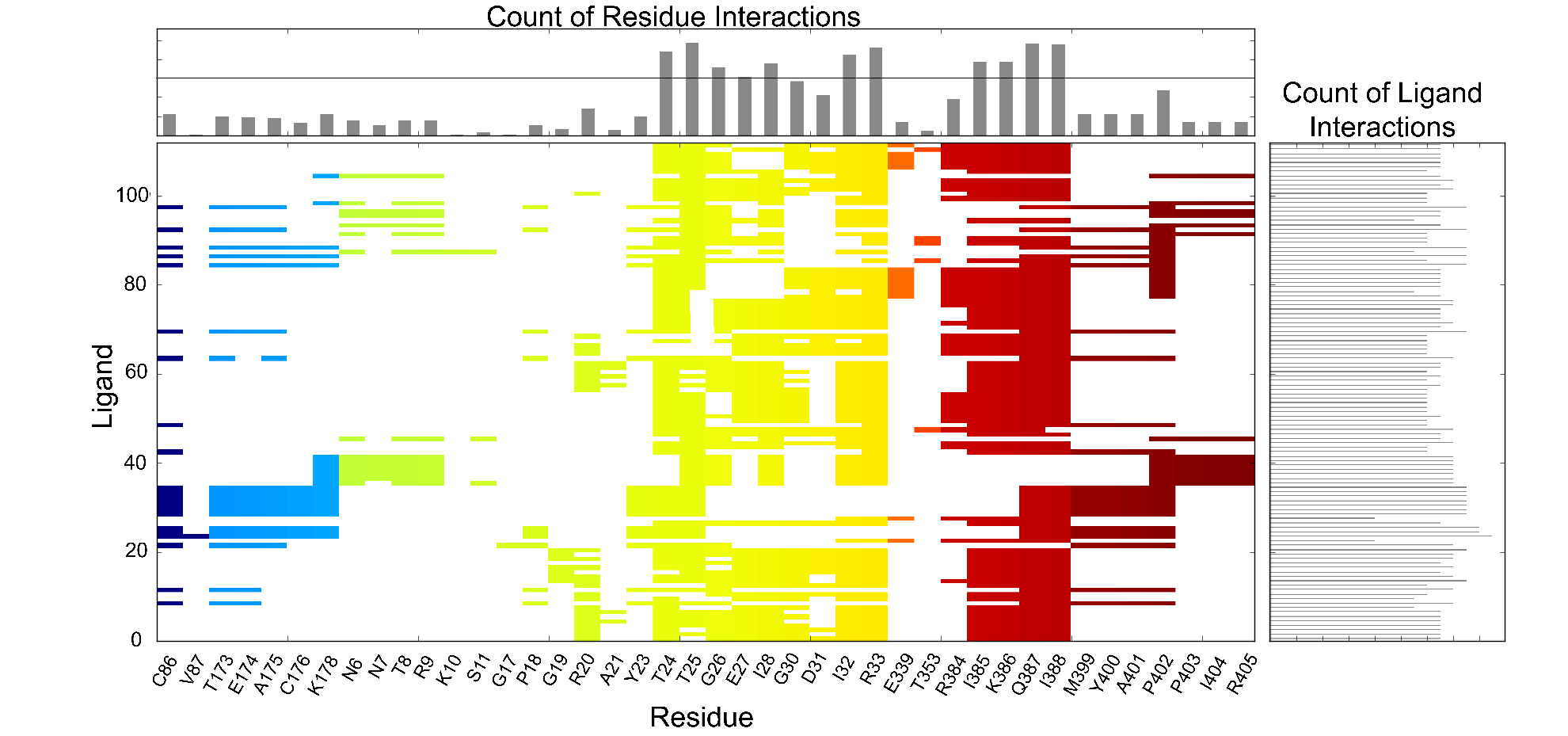


**Figure X.** Ligand fingerprinting of docking results from replicate 1 of gp120_BaL_ following MD simulation. Residues in the V3 loop (N6–R33) are renumbered according to Figure S1, and a 50% threshold of interactions across poses is marked with a black line.

**gp120_BaL_: Representative MD Cluster, Charmm Replicate 2 (Figure S40)**


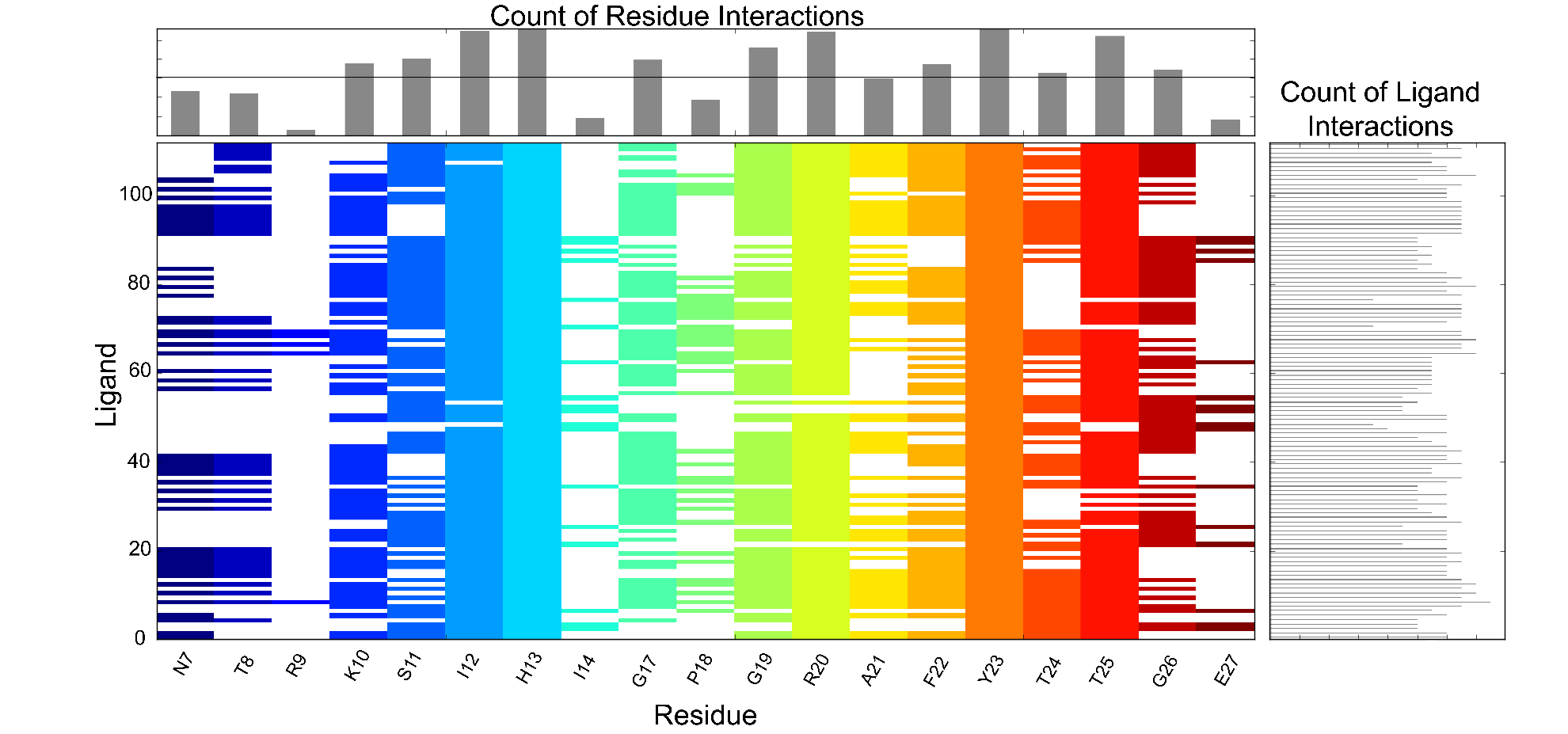


**Figure Y.** Ligand fingerprinting of docking results from replicate 2 of gp120_BaL_ following MD simulation. Residues in the V3 loop (N7–E27) are renumbered according to Figure S1, and a 50% threshold of interactions across poses is marked with a black line in the upper part of the figure.

**gp120_BaL_: Representative MD Cluster, Charmm Replicate 3 (Figure S40)**


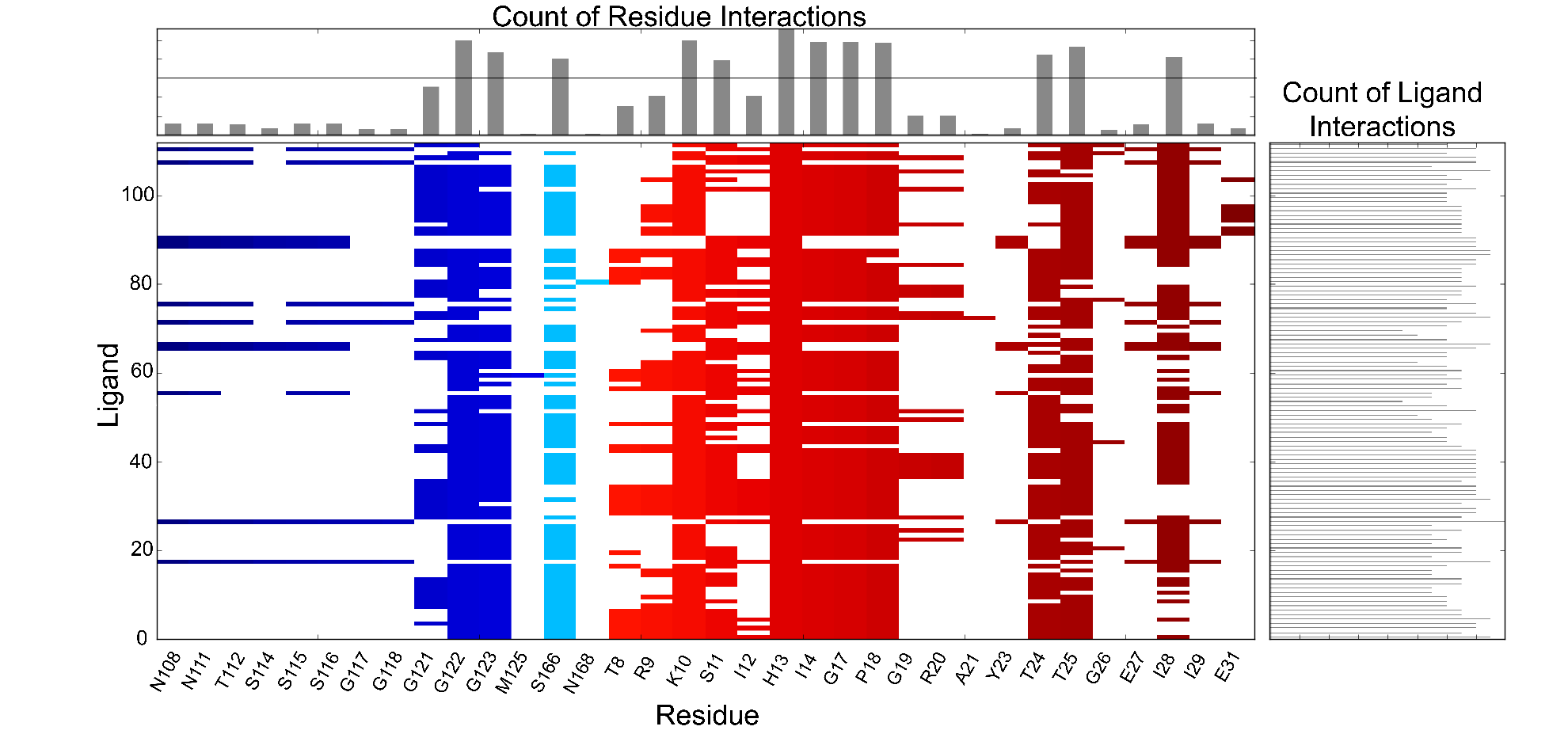


**Figure Z.** Ligand fingerprinting of docking results from replicate 3 of gp120_BaL_ following MD simulation. Residues in the V3 loop (T8–E31) are renumbered according to Figure S1, and a 50% threshold of interactions across poses is marked with a black line in the upper part of the figure.

**gp120_IIIb_: Representative MD Cluster, Charmm Replicate 1 (Figure S40)**

_
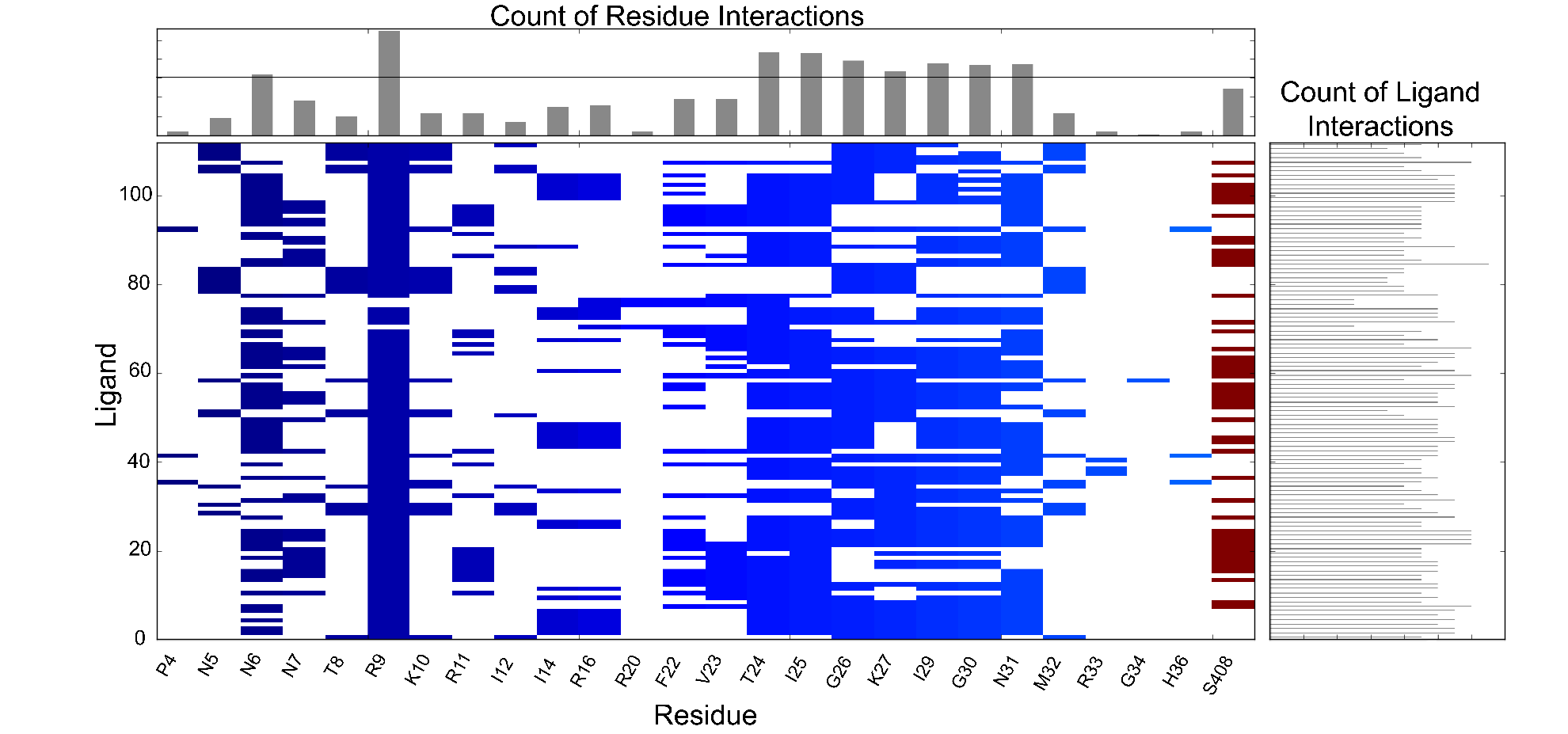
_

**Figure AA.** Ligand fingerprinting of docking results from replicate 1 of gp120_IIIb_ following MD simulation. Residues in the V3 loop (P4–H36) are renumbered according to Figure S1, and a 50% threshold of interactions across poses is marked with a black line in the upper part of the figure.

**gp120_IIIb_: Representative MD Cluster, Charmm Replicate 2 (Figure S40)**


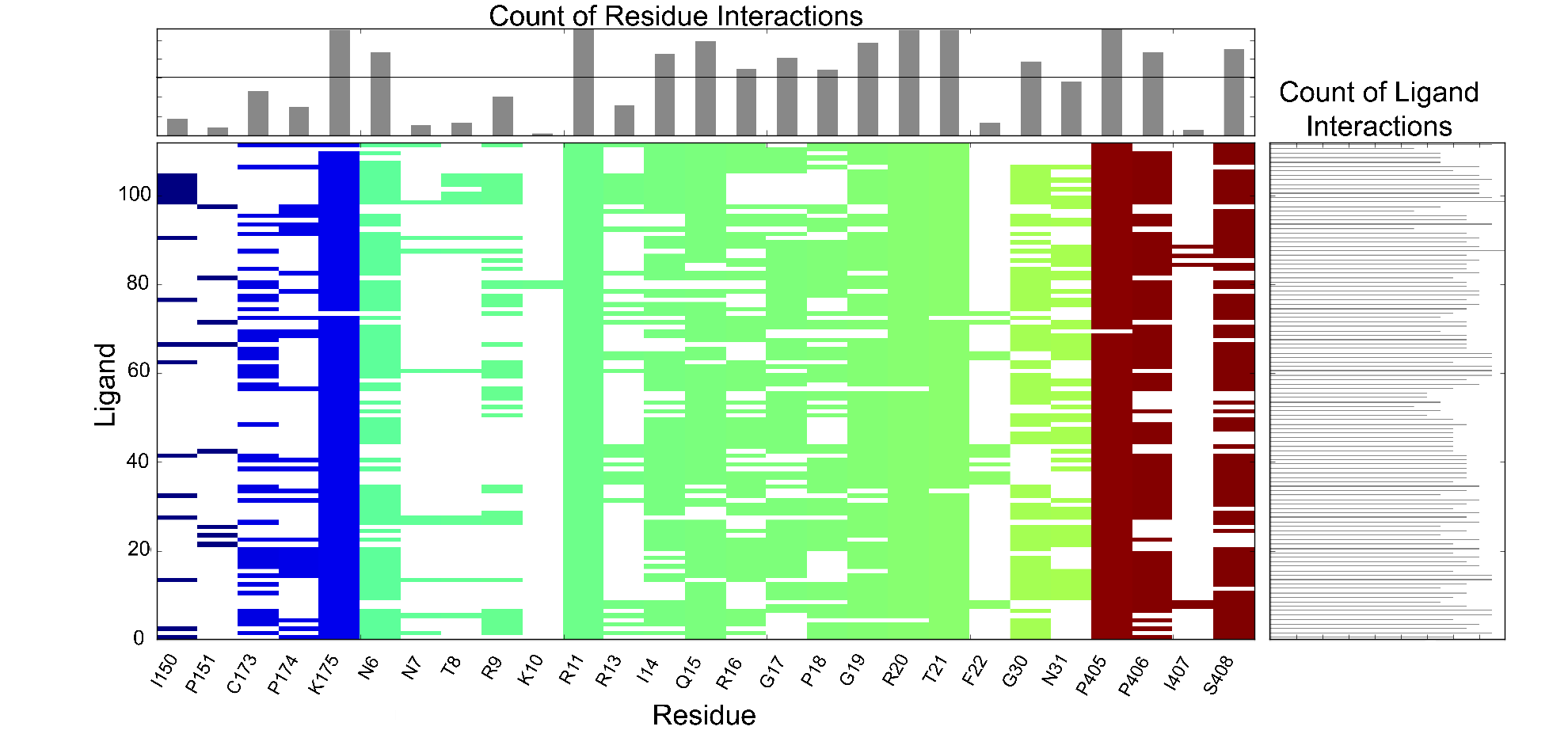


**Figure BB.** Ligand fingerprinting of docking results from replicate 2 of gp120_IIIb_ following MD simulation. Residues in the V3 loop (N6–N31) are renumbered according to Figure S1, and a 50% threshold of interactions across poses is marked with a black line in the upper part of the figure.

**gp120_IIIb_: Representative MD Cluster, Charmm Replicate 3 (Figure S40)**


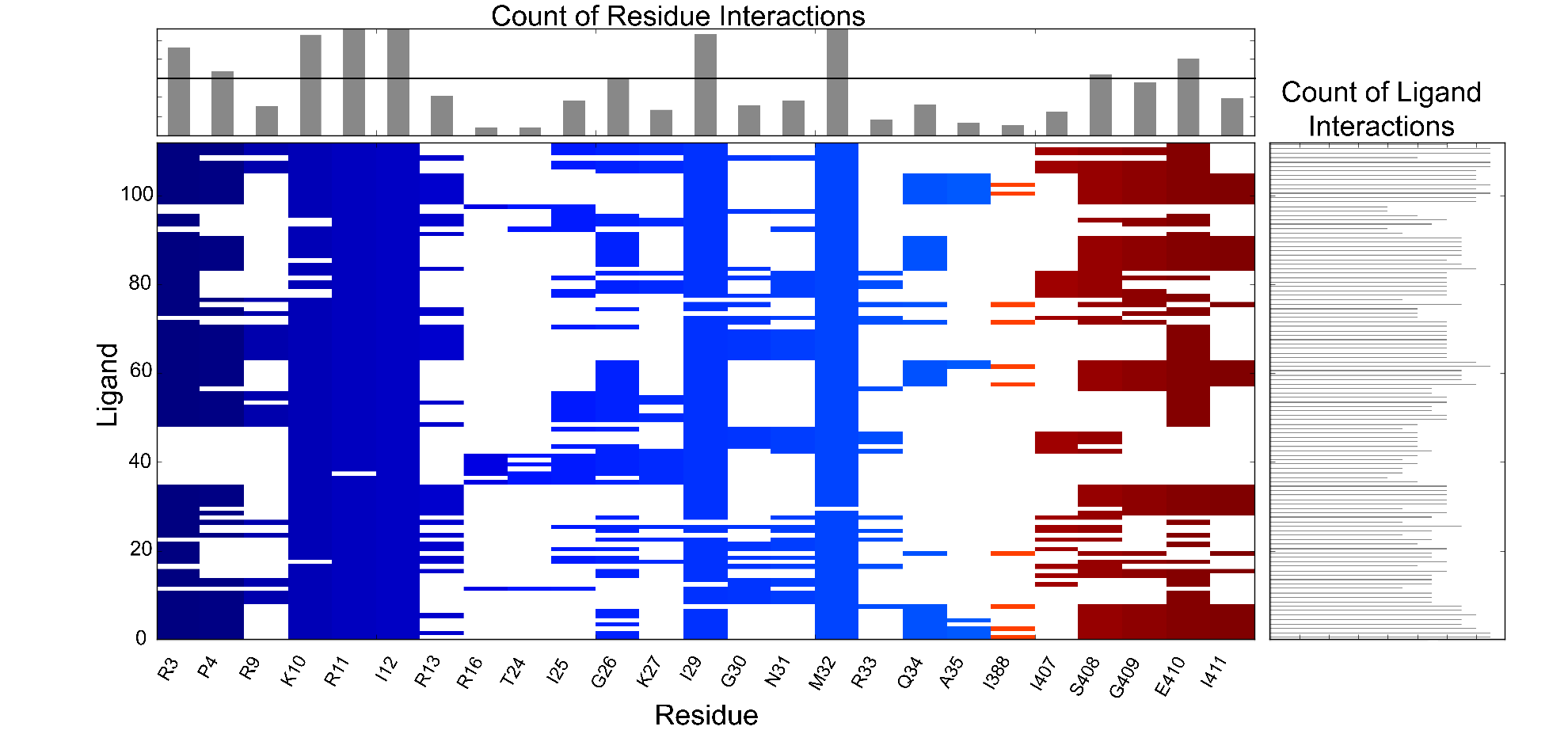


**Figure CC.** Ligand fingerprinting of docking results from replicate 3 of gp120_IIIb_ following MD simulation. Residues in the V3 loop (R3–A35) are renumbered according to Figure S1, and a 50% threshold of interactions across poses is marked with a black line in the upper part of the figure.

**gp120_JR-CSF_: Representative MD Cluster, Charmm Replicate 1 (Figure S40)**


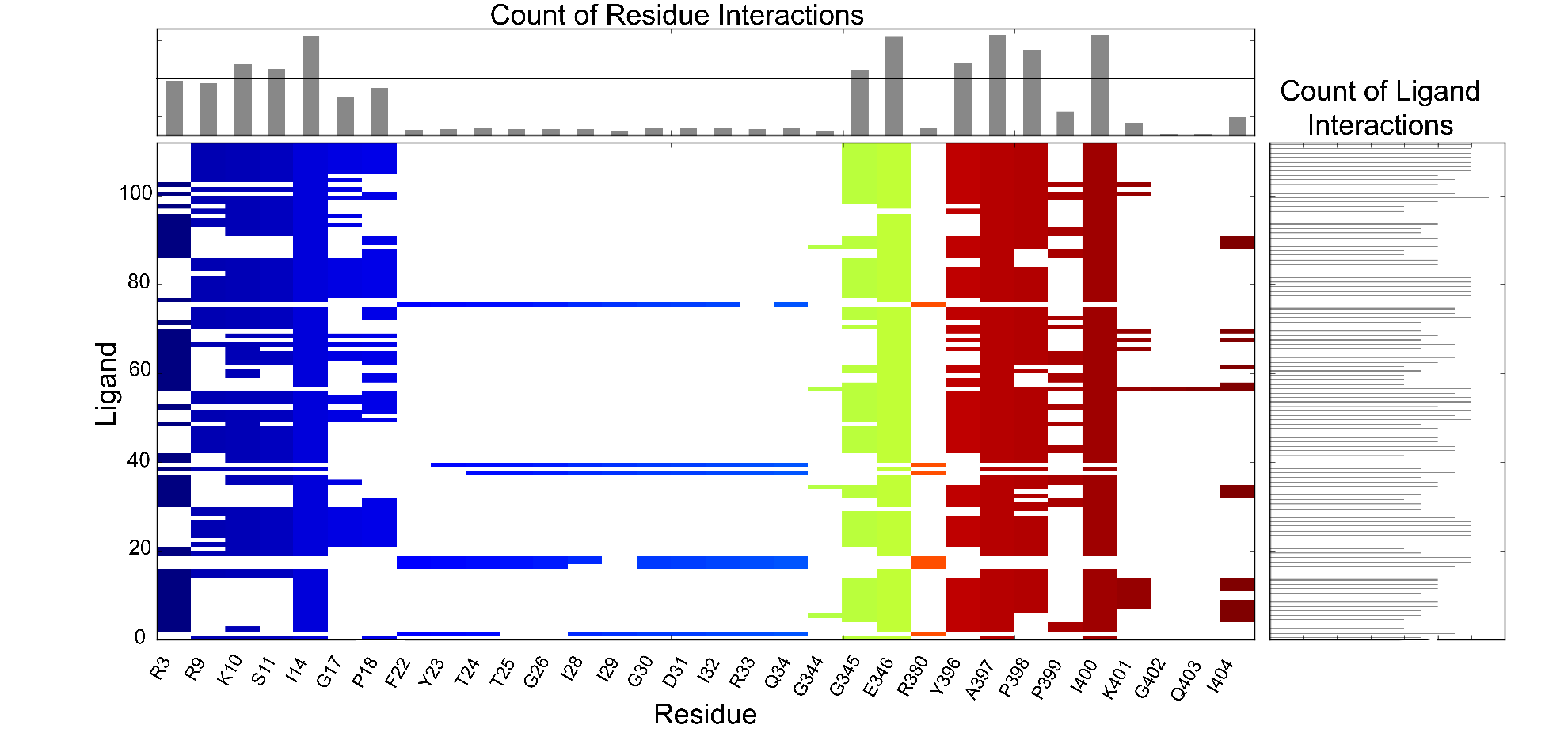


**Figure DD.** Ligand fingerprinting of docking results from replicate 1 of gp120_JR-CSF_ following MD simulation. Residues in the V3 loop (R3–Q34) are renumbered according to Figure S1, and a 50% threshold of interactions across poses is marked with a black line in the upper part of the figure.

**gp120_JR-CSF_: Representative MD Cluster, Charmm Replicate 2 (Figure S40)**


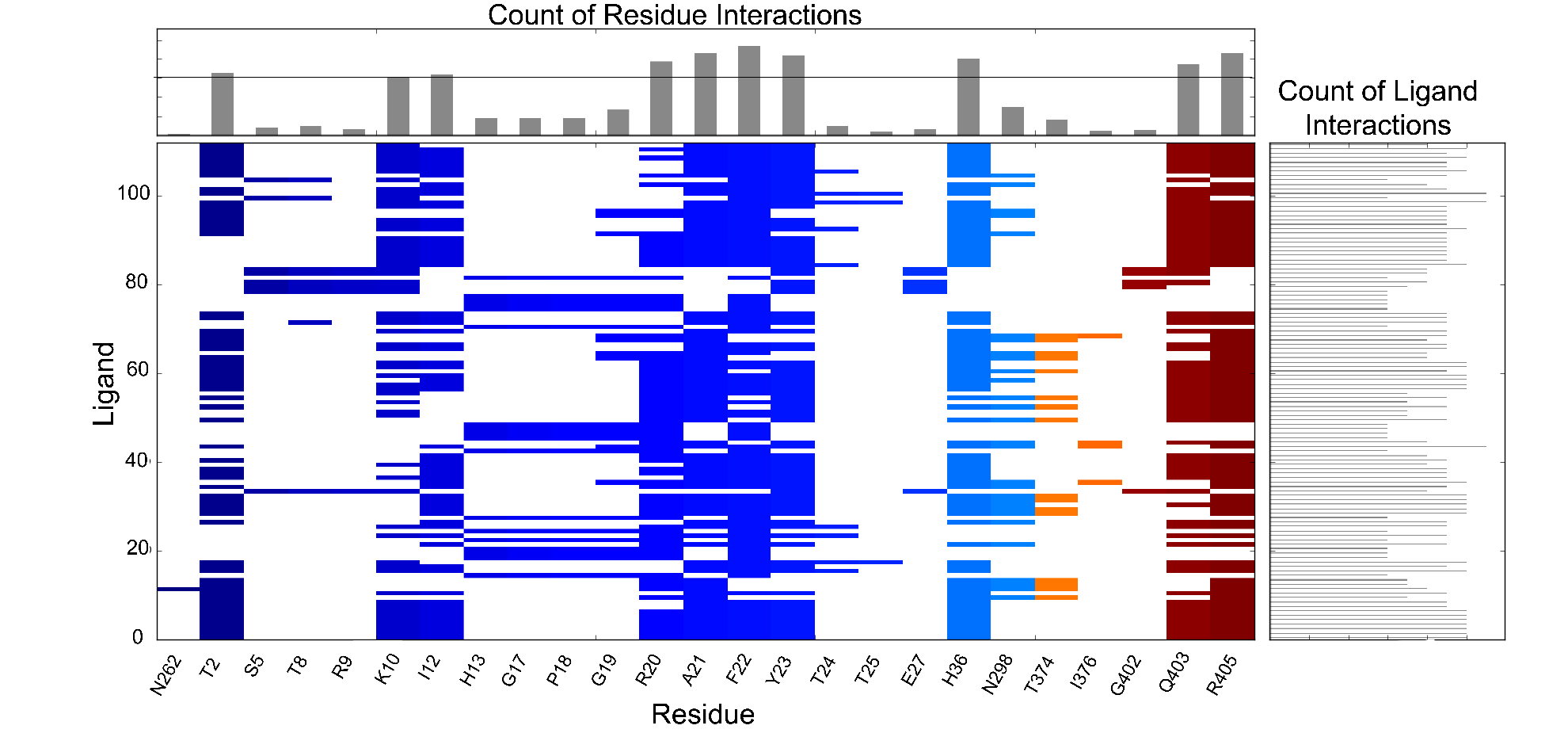


**Figure EE.** Ligand fingerprinting of docking results from replicate 2 of gp120_JR-CSF_ following MD simulation. Residues in the V3 loop (T2–H36) are renumbered according to Figure S1, and a 50% threshold of interactions across poses is marked with a black line in the upper part of the figure.

**gp120_JR-CSF_: Representative MD Cluster, Charmm Replicate 3 (Figure S40)**


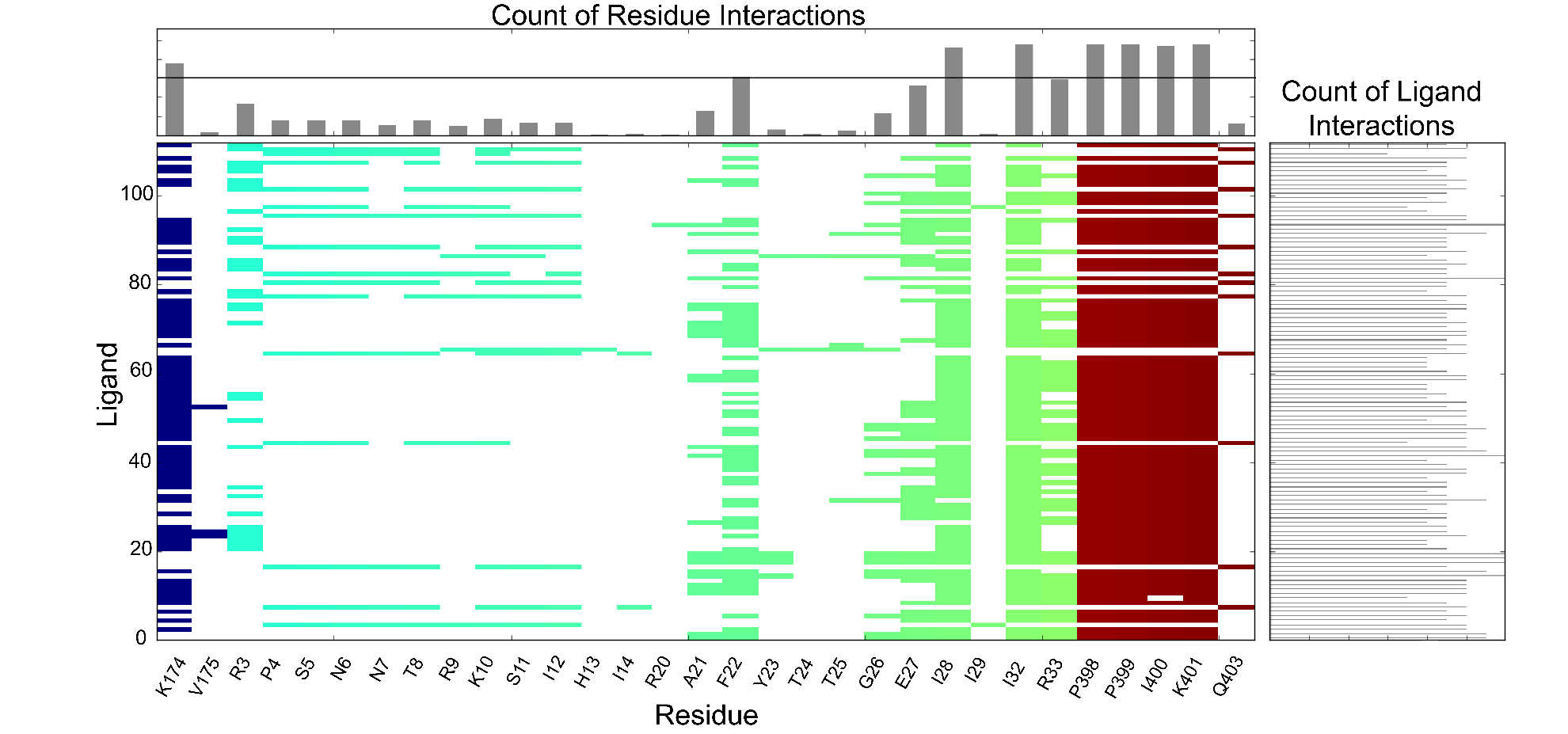


**Figure FF.** Ligand fingerprinting of docking results from replicate 3 of gp120_JR-CSF_ following MD simulation. Residues in the V3 loop (R3–R33) are renumbered according to Figure S1, and a 50% threshold of interactions across poses is marked with a black line in the upper part of the figure.

**gp120_YU2_: Representative MD Cluster, Charmm Replicate 1 (Figure S40)**


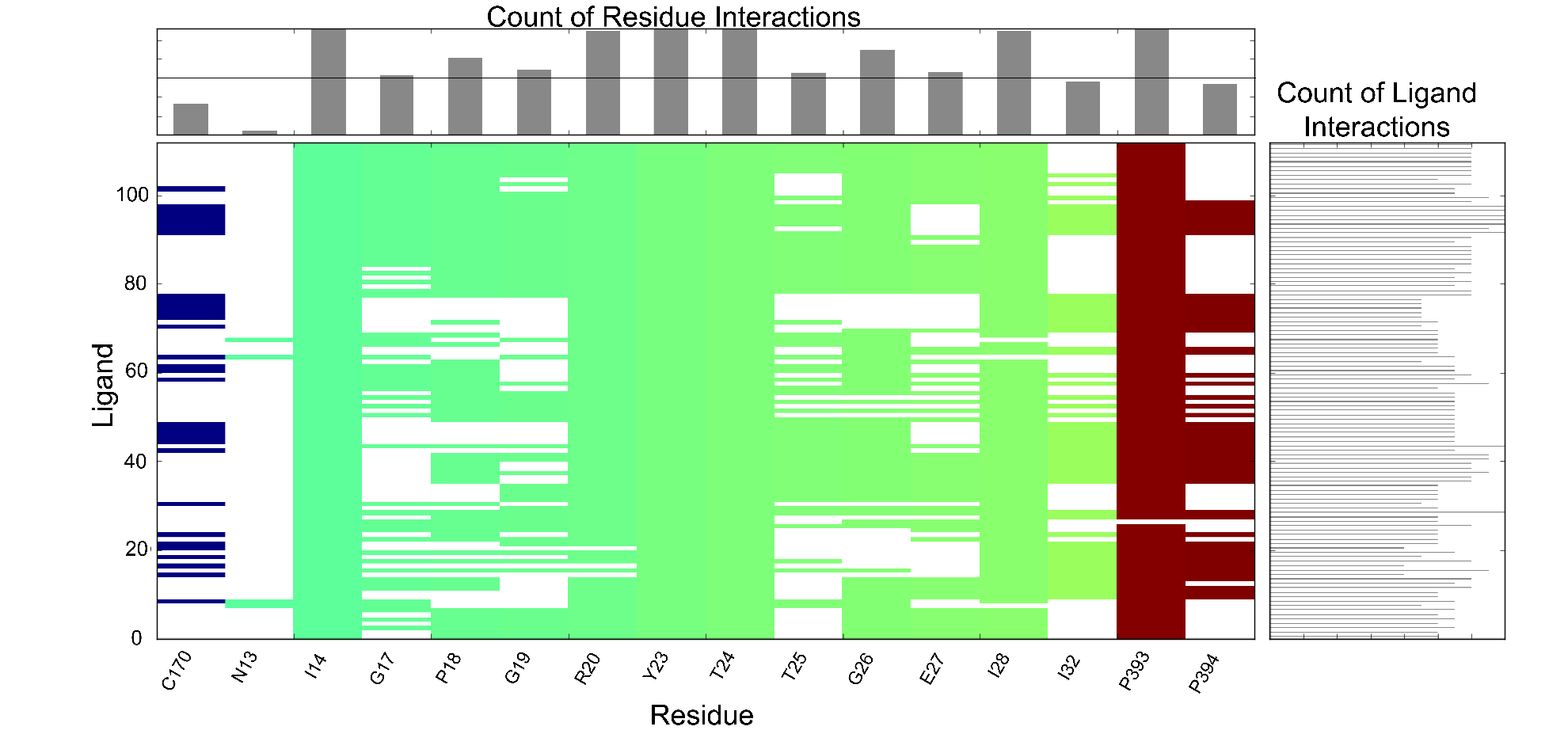


**Figure GG.** Ligand fingerprinting of docking results from replicate 1 of gp120_YU2_ following MD simulation. Residues in the V3 loop (N13–I32) are renumbered according to Figure S1, and a 50% threshold of interactions across poses is marked with a black line in the upper part of the figure.

**gp120_YU2_: Representative MD Cluster, Charmm Replicate 2 (Figure S40)**


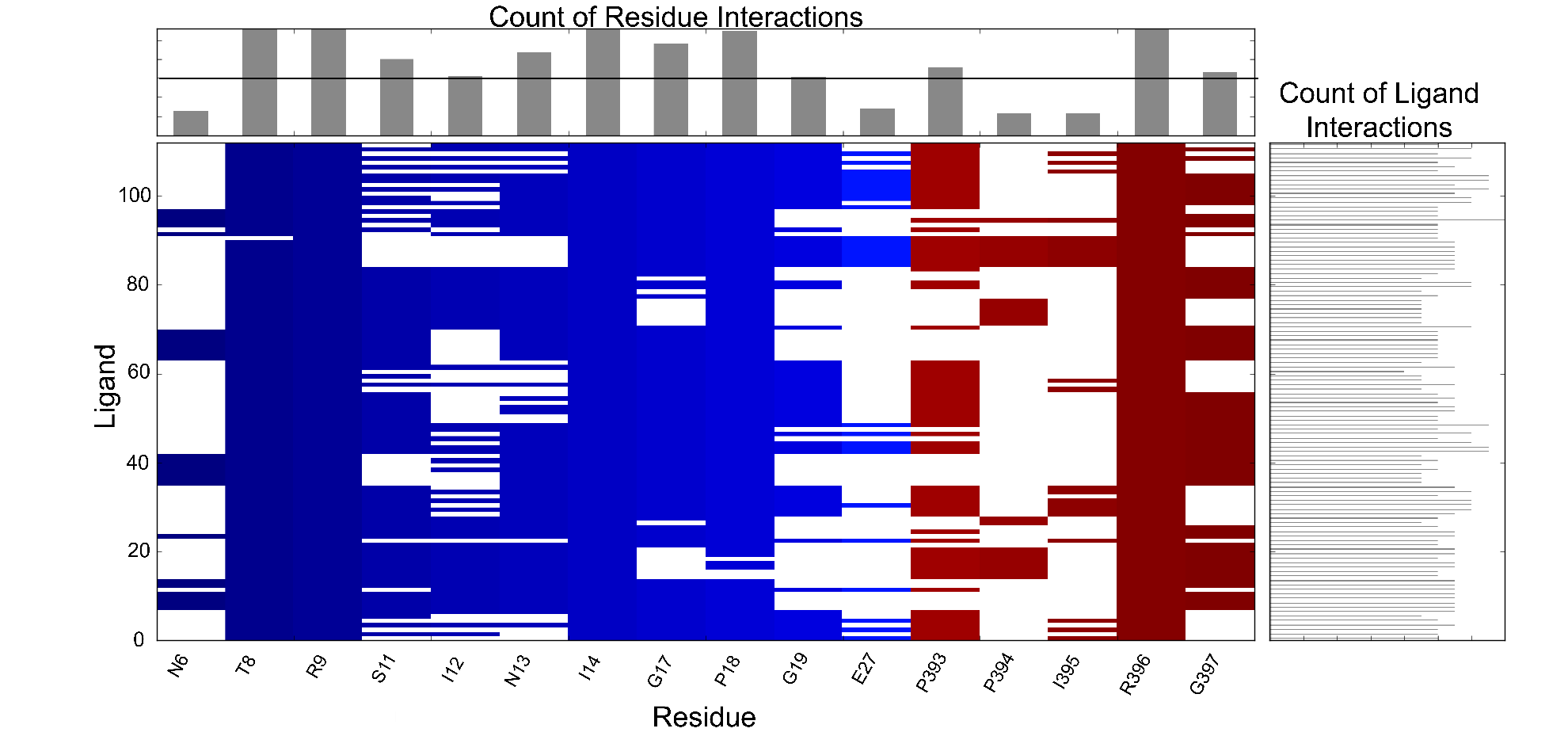


**Figure HH.** Ligand fingerprinting of docking results from replicate 2 of gp120_YU2_ following MD simulation. Residues in the V3 loop (N6–E27) are renumbered according to Figure S1, and a 50% threshold of interactions across poses is marked with a black line in the upper part of the figure.

**gp120_YU2_: Representative MD Cluster, Charmm Replicate 3 (Figure S40)**


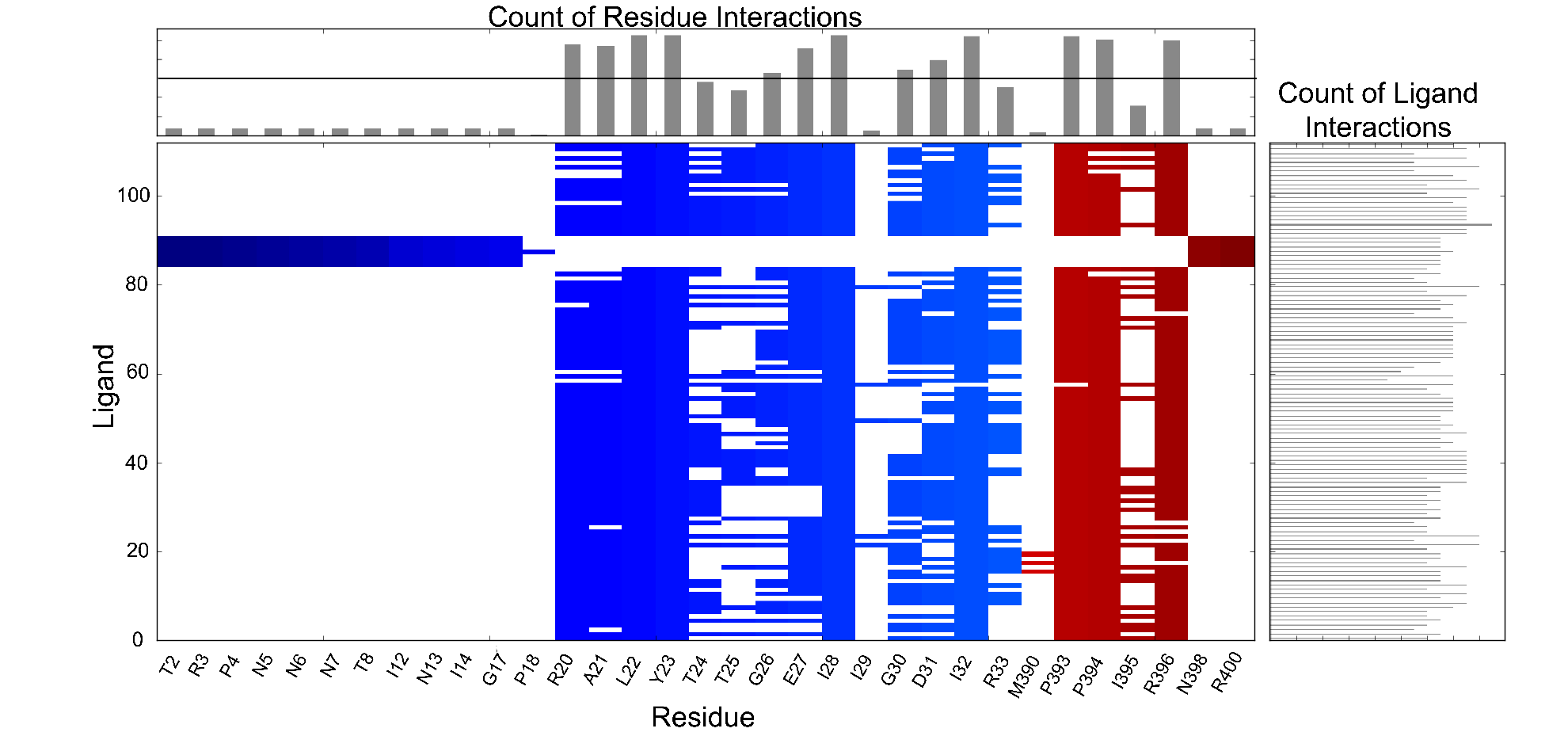


**Figure II.** Ligand fingerprinting of docking results from replicate 3 of gp120_YU2_ following MD simulation. Residues in the V3 loop (T2–R33) are renumbered according to Figure S1, and a 50% threshold of interactions across poses is marked with a black line in the upper part of the figure.

**Table F.** Residue interactions are numbered based on aligned V3 loop sequences (Figure S1) from all docking simulations on dominant morphologies derived from MD simulation (Figures S18-S35). Residues identified below interact with >50% of ligands and >40% if denoted with an asterisk.

| **Protein** | **Replicate** | **H-Bond** | **Hydrophilic** | **Hydrophobic** | **Backbone Interaction** |
| --- | --- | --- | --- | --- | --- |
| **2B4C** | 1 | R20 | S11, H13, D31, R33 | A21, I28, I32 | S11, I12, I29, G30, Y23, M434* |
|  | 2 | - | - | P369 | - |
|  | 3 |  | T8, K10, S11, H13, D31, Q34* | I28, I29, I32 | R9 |
| **92UG037** | 1 | R13, R33 | R9, T21, Q383, Q401 | I14, I32, P398 | Y396* |
|  | 2 | R9, H36 | N5, N7, T8, T25, D336 | Y23, A24, P378 | N6, G334, G335 |
|  | 3 | R380 | R10, D27, D31, R33, K382, Q383 | A24*, L337, Y352 | V12*, I381*, I384 |
| **BaL** | 1 | - | T24, T25, R33, E27, K386, Q387 | I28, I32, I388 | G26, G30*, I385 |
|  | 2 | R20, F22, Y23, G26 | K10, H13, T25 | I12, Y23 | S11, G17, G19, A21, T24 |
|  | 3 | G123, T25 | S166, K10, H13, T24 | P18, I28 | G122, S11, I14, G17 |
| **IIIb** | 1 | R9 | N6, T24, K27, N31 | I25, I29 | G26, G30 |
|  | 2 | K175, N6, R11, G19, R20, S408 | R16, T21, N31* | I14, P405 | Q15, G17, P18, G30, P406 |
|  | 3 | R11 | R3, E410 | P4, I12, I29, M32 | K10, G26, S408, G409* |
| **JR-CSF** | 1 | S11 | R3*, E346 | I14, Y396, A397, I400 | R9*, K10, G345, P398 |
|  | 2 | Q403, R405 | T2, K10, R20, H36 | I12, F22, Y23 | A21 |
|  | 3 | R33*, K401 | K174 | F22, I28, I32, P398, P399, I400 | - |
| **YU2** | 1 | R20 | T24, E27 | I14, I28, I32*, P393, P394*, Y23 | G17, P18, G19, T25, G26 |
|  | 2 | N13 | T8, R9, R396 | P18, P393 | S11, I12, I14, G17, G19, G397 |
|  | 3 | Y23 | E27, D31, R396 | L22, I28, I32, P393 | R20, A21, T24*, G26, G30, P394 |

**Table G.** Summary of key interactions that occur in at least two of three replicates from MD-derived structures. Residues are numbered based on aligned V3 loop sequences (Figure S1)

| **Strain** | **IC_50_ (ng/mL)^8^** | **H-Bond** | **Hydrophilic** | **Hydrophobic** | **Backbone Interaction** |
| --- | --- | --- | --- | --- | --- |
| 2B4C | Unknown | - | S11, H13, D31 | I28, I29, I32 | - |
| 92UG037 | 750 | - | Q383 | - | - |
| BaL | 230 | - | - | I28 | S11, G17, G26 |
| IIIb | 95 | R11 | N6, R11, N31, S408 | I29 | G26, G30 |
| JR-CSF | 760 | - | K10 | F22, P398, I400, F22 | - |
| YU2 | Unknown | - | R20, T24, E27, R396 | I14, P18, Y23, I28, I32, P393 | G17, G19, G26, P394 |

**RMSD Plots**

**CHARMM36**


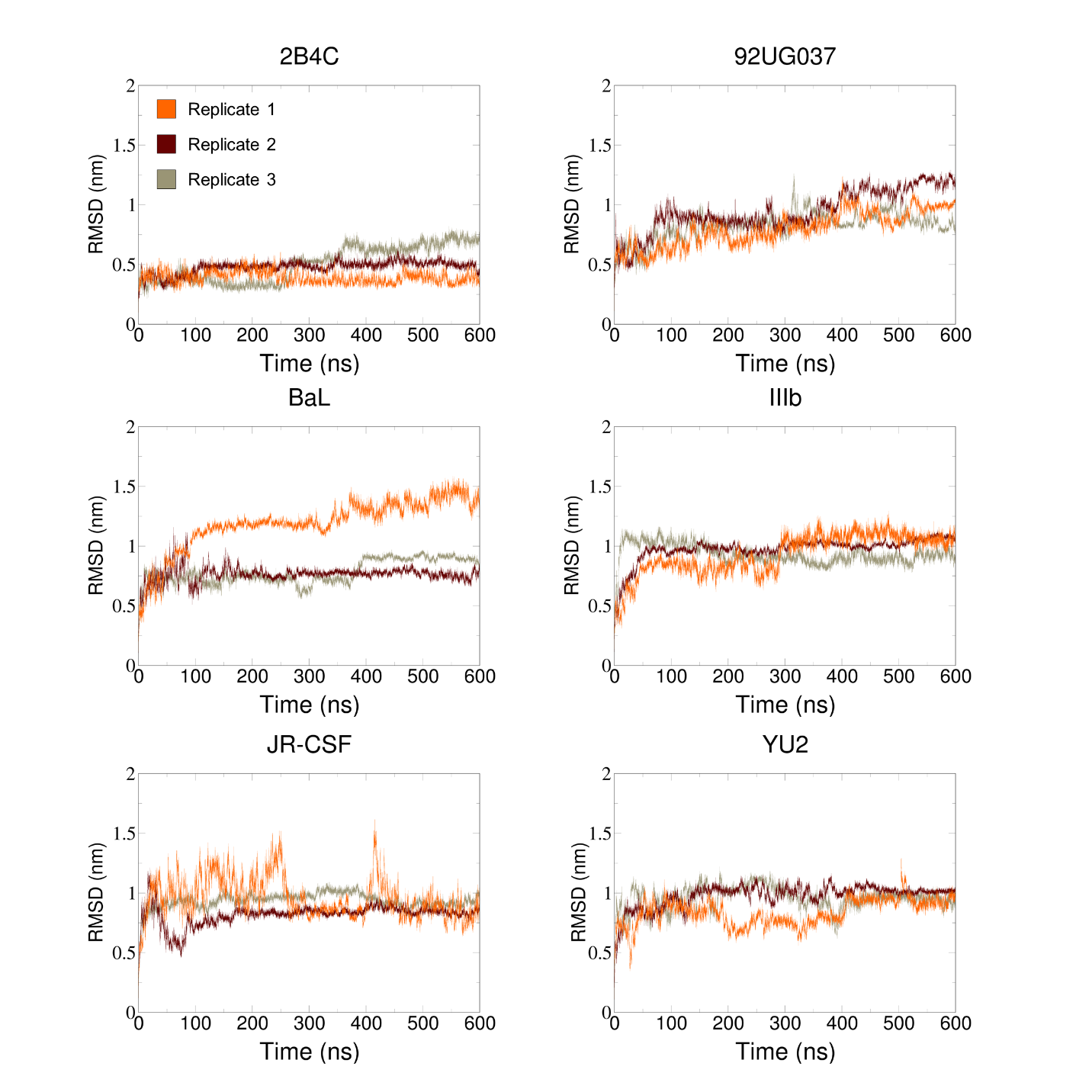


**Figure JJ.** RMSD following 600 ns of simulation (CHARMM36) with three replicates of each structure.

**Amber99SB-ILDN**


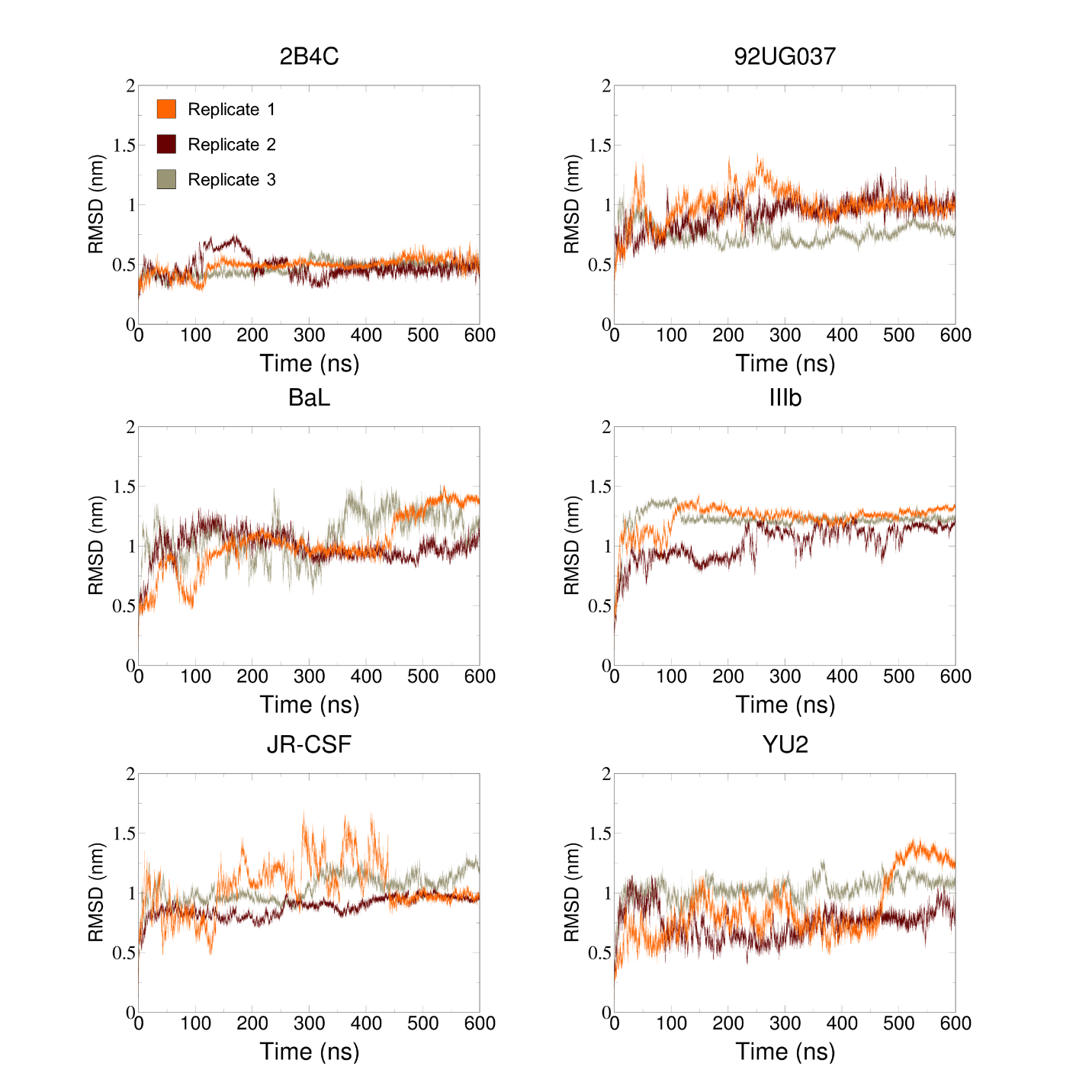


**Figure KK.** RMSD following 600 ns of simulation (Amber99SB-ILDN) with three replicates of each structure.

**Block Averaging and Convergence**

Block averaging was conducted to provide a quantitative measure of structural convergence. Structures were determined to be converged when every 25 ns block average RMSD between 500-600 ns was within standard deviation of another, except in noted cases where the standard deviation was small due to minute fluctuations over the final timespan.

**Table H.** Block average RMSD values in 25 ns increments over the last 100 ns of each simulation (CHARMM36 force field).

| **Simulation** | **Replicate** | **Time Range** | **RMSD (Å)**^†^ |
| --- | --- | --- | --- |
| **2B4C** | 1 | 500-525 ns | 0.67±0.04 |
|  |  | 525-550 ns | 0.69±0.04 |
|  |  | 550-575 ns | 0.72±0.03 |
|  |  | 575-600 ns | 0.70±0.03 |
|  | 2 | 500-525 ns | 0.67±0.04 |
|  |  | 525-550 ns | 0.69±0.04 |
|  |  | 550-575 ns | 0.72±0.03 |
|  |  | 575-600 ns | 0.70±0.03 |
|  | 3 | 500-525 ns | 0.39±0.03 |
|  |  | 525-550 ns | 0.37±0.04 |
|  |  | 550-575 ns | 0.35±0.03 |
|  |  | 575-600 ns | 0.39±0.04 |
| **92UG037** | 1 | 500-525 ns | 0.89±0.05 |
|  |  | 525-550 ns | 0.85±0.04 |
|  |  | 550-575 ns | 0.85±0.04 |
|  |  | 575-600 ns | 0.82±0.04 |
|  | 2 | 500-525 ns | 1.13±0.05 |
|  |  | 525-550 ns | 1.21±0.03 |
|  |  | 550-575 ns | 1.20±0.03 |
|  |  | 575-600 ns | 1.20±0.04 |
|  | 3 | 500-525 ns | 0.92±0.07 |
|  |  | 525-550 ns | 0.99±0.05 |
|  |  | 550-575 ns | 0.98±0.02 |
|  |  | 575-600 ns | 1.00±0.02 |
| **BaL** | 1 | 500-525 ns | 0.90±0.02 |
|  |  | 525-550 ns | 0.91±0.01 |
|  |  | 550-575 ns | 0.90±0.02 |
|  |  | 575-600 ns | 0.88±0.02 |
|  | 2 | 500-525 ns | 0.76±0.03 |
|  |  | 525-550 ns | 0.76±0.02 |
|  |  | 550-575 ns | 0.74±0.03 |
|  |  | 575-600 ns | 0.76±0.04 |
|  | 3 | 500-525 ns | 1.38±0.06 |
|  |  | 525-550 ns | 1.43±0.05 |
|  |  | 550-575 ns | 1.46±0.04 |
|  |  | 575-600 ns | 1.37±0.07 |
| **IIIb** | 1 | 500-525 ns | 0.87±0.04 |
|  |  | 525-550 ns | 0.92±0.04 |
|  |  | 550-575 ns | 0.93±0.03 |
|  |  | 575-600 ns | 0.94±0.04 |
|  | 2 | 500-525 ns | 1.03±0.02 |
|  |  | 525-550 ns | 1.05±0.01 |
|  |  | 550-575 ns | 1.08±0.01 |
|  |  | 575-600 ns | 1.07±0.01 |
|  | 3 | 500-525 ns | 1.09±0.04 |
|  |  | 525-550 ns | 1.05±0.04 |
|  |  | 550-575 ns | 1.06±0.03 |
|  |  | 575-600 ns | 1.05±0.05 |
| **JR-CSF** | 1 | 500-525 ns | 0.90±0.02 |
|  |  | 525-550 ns | 0.91±0.02 |
|  |  | 550-575 ns | 0.91±0.03 |
|  |  | 575-600 ns | 0.95±0.03 |
|  | 2 | 500-525 ns | 0.86±0.02 |
|  |  | 525-550 ns | 0.84±0.02 |
|  |  | 550-575 ns | 0.83±0.02 |
|  |  | 575-600 ns | 0.83±0.03 |
|  | 3 | 500-525 ns | 0.91±0.06 |
|  |  | 525-550 ns | 0.85±0.07 |
|  |  | 550-575 ns | 0.83±0.09 |
|  |  | 575-600 ns | 0.81±0.08 |
| **YU2** | 1 | 500-525 ns | 0.97±0.03 |
|  |  | 525-550 ns | 0.95±0.03 |
|  |  | 550-575 ns | 0.97±0.03 |
|  |  | 575-600 ns | 0.91±0.04 |
|  | 2 | 500-525 ns | 1.01±0.02 |
|  |  | 525-550 ns | 1.01±0.02 |
|  |  | 550-575 ns | 1.01±0.02 |
|  |  | 575-600 ns | 1.02±0.01 |
|  | 3 | 500-525 ns | 1.01±0.08 |
|  |  | 525-550 ns | 0.90±0.03 |
|  |  | 550-575 ns | 0.92±0.03 |
|  |  | 575-600 ns | 0.94±0.03 |

^†^All values are ± one standard deviation.

**Table I.** Block average RMSD values in 25 ns increments over the last 100 ns of each simulation (Amber99SB-ILDN force field).

| **Simulation** | **Replicate** | **Time Range** | **RMSD (Å)** ^†^ |
| --- | --- | --- | --- |
| **2B4C** | 1 | 500-525 ns | 0.52±0.02 |
|  |  | 525-550 ns | 0.50±0.02 |
|  |  | 550-575 ns | 0.49±0.02 |
|  |  | 575-600 ns | 0.50±0.02 |
|  | 2 | 500-525 ns | 0.45±0.03 |
|  |  | 525-550 ns | 0.46±0.04 |
|  |  | 550-575 ns | 0.49±0.05 |
|  |  | 575-600 ns | 0.48±0.05 |
|  | 3 | 500-525 ns | 0.55±0.03 |
|  |  | 525-550 ns | 0.56±0.03 |
|  |  | 550-575 ns | 0.52±0.05 |
|  |  | 575-600 ns | 0.52±0.06 |
| **92UG037** | 1 | 500-525 ns | 0.82±0.03 |
|  |  | 525-550 ns | 0.84±0.02 |
|  |  | 550-575 ns | 0.81±0.02 |
|  |  | 575-600 ns | 0.78±0.02 |
|  | 2 | 500-525 ns | 1.02±0.06 |
|  |  | 525-550 ns | 0.99±0.06 |
|  |  | 550-575 ns | 0.98±0.05 |
|  |  | 575-600 ns | 1.00±0.05 |
|  | 3 | 500-525 ns | 1.00±0.03 |
|  |  | 525-550 ns | 1.00±0.04 |
|  |  | 550-575 ns | 0.97±0.04 |
|  |  | 575-600 ns | 0.97±0.04 |
| **BaL** | 1 | 500-525 ns | 1.27±0.06 |
|  |  | 525-550 ns | 1.31±0.08 |
|  |  | 550-575 ns | 1.19±0.08 |
|  |  | 575-600 ns | 1.12±0.07 |
|  | 2 | 500-525 ns | 1.01±0.04 |
|  |  | 525-550 ns | 0.96±0.05 |
|  |  | 550-575 ns | 0.98±0.04 |
|  |  | 575-600 ns | 1.03±0.05 |
|  | 3 | 500-525 ns | 1.35±0.04 |
|  |  | 525-550 ns | 1.41±0.03 |
|  |  | 550-575 ns | 1.38±0.03 |
|  |  | 575-600 ns | 1.39±0.02 |
| **IIIb** | 1 | 500-525 ns | 1.22±0.01 |
|  |  | 525-550 ns | 1.24±0.03 |
|  |  | 550-575 ns | 1.23±0.02 |
|  |  | 575-600 ns | 1.22±0.02 |
|  | 2 | 500-525 ns | 1.12±0.05 |
|  |  | 525-550 ns | 1.17±0.02 |
|  |  | 550-575 ns | 1.15±0.03 |
|  |  | 575-600 ns | 1.16±0.02 |
|  | 3 | 500-525 ns | 1.29±0.02 |
|  |  | 525-550 ns | 1.27±0.01 |
|  |  | 550-575 ns | 1.31±0.01 |
|  |  | 575-600 ns | 1.32±0.01 |
| **JR-CSF** | 1 | 500-525 ns | 1.06±0.04 |
|  |  | 525-550 ns | 1.10±0.04 |
|  |  | 550-575 ns | 1.15±0.05 |
|  |  | 575-600 ns | 1.24±0.03 |
|  | 2 | 500-525 ns | 0.99±0.02 |
|  |  | 525-550 ns | 0.97±0.01 |
|  |  | 550-575 ns | 0.96±0.01 |
|  |  | 575-600 ns | 0.95±0.01 |
|  | 3 | 500-525 ns | 0.97±0.03 |
|  |  | 525-550 ns | 0.95±0.02 |
|  |  | 550-575 ns | 0.98±0.03 |
|  |  | 575-600 ns | 0.98±0.03 |
| **YU2** | 1 | 500-525 ns | 1.11±0.06 |
|  |  | 525-550 ns | 1.13±0.03 |
|  |  | 550-575 ns | 1.08±0.03 |
|  |  | 575-600 ns | 1.08±0.03 |
|  | 2 | 500-525 ns | 0.76±0.04 |
|  |  | 525-550 ns | 0.77±0.06 |
|  |  | 550-575 ns | 0.87±0.06 |
|  |  | 575-600 ns | 0.88±0.07 |
|  | 3 | 500-525 ns | 1.32±0.05 |
|  |  | 525-550 ns | 1.36±0.04 |
|  |  | 550-575 ns | 1.33±0.04 |
|  |  | 575-600 ns | 1.37±0.04 |

^†^All values are ± one standard deviation.

**Clustering**

Trajectories were clustered over the last 100 ns (500-600 ns) with a 0.2 nm backbone RMSD cutoff using the gromos method.^17^

**Table J.** Dominant cluster sizes (morphologies) for CHARMM36 force field simulations.

| **Protein** | **Replicate** | **Cluster Size** | **# Frames in Dominant Cluster** | **% Dominant Morphology** |
| --- | --- | --- | --- | --- |
| 2B4C | 1 | 10001 | 6253 | 62.5 |
|  | 2 | 10001 | 9174 | 91.7 |
|  | 3 | 10001 | 5844 | 58.4 |
| 92UG037 | 1 | 10001 | 2106 | 21.1 |
|  | 2 | 10001 | 3708 | 37.1 |
|  | 3 | 10001 | 4187 | 41.9 |
| BaL | 1 | 10001 | 7487 | 74.9 |
|  | 2 | 10001 | 5422 | 54.2 |
|  | 3 | 10001 | 1456 | 14.6 |
| IIIb | 1 | 10001 | 3102 | 31.0 |
|  | 2 | 10001 | 3125 | 31.2 |
|  | 3 | 10001 | 2350 | 23.5 |
| JR-CSF | 1 | 10001 | 8457 | 84.6 |
|  | 2 | 10001 | 8368 | 83.7 |
|  | 3 | 10001 | 1273 | 12.7 |
| YU2 | 1 | 10001 | 5116 | 51.2 |
|  | 2 | 10001 | 9933 | 99.3 |
|  | 3 | 10001 | 3203 | 32.0 |

**Table K.** Dominant cluster sizes (morphologies) for Amber99SB-ILDN force field simulations.

| **Protein** | **Replicate** | **Cluster Size** | **# Frames in Dominant Cluster** | **% Dominant Morphology** |
| --- | --- | --- | --- | --- |
| 2B4C | 1 | 10001 | 9447 | 94.5 |
|  | 2 | 10001 | 2576 | 25.8 |
|  | 3 | 10001 | 1636 | 16.4 |
| 92UG037 | 1 | 10001 | 4978 | 50.0 |
|  | 2 | 10001 | 1760 | 17.6 |
|  | 3 | 10001 | 2268 | 22.7 |
| BaL | 1 | 10001 | 1011 | 10.1 |
|  | 2 | 10001 | 2088 | 20.9 |
|  | 3 | 10001 | 7382 | 73.8 |
| IIIb | 1 | 10001 | 4296 | 43.0 |
|  | 2 | 10001 | 3017 | 30.2 |
|  | 3 | 10001 | 5494 | 54.9 |
| JR-CSF | 1 | 10001 | 2202 | 22.0 |
|  | 2 | 10001 | 6329 | 63.8 |
|  | 3 | 10001 | 3814 | 38.1 |
| YU2 | 1 | 10001 | 4100 | 41.1 |
|  | 2 | 10001 | 2422 | 24.2 |
|  | 3 | 10001 | 2752 | 27.5 |

**RMSF Plots**

**CHARMM36**

**
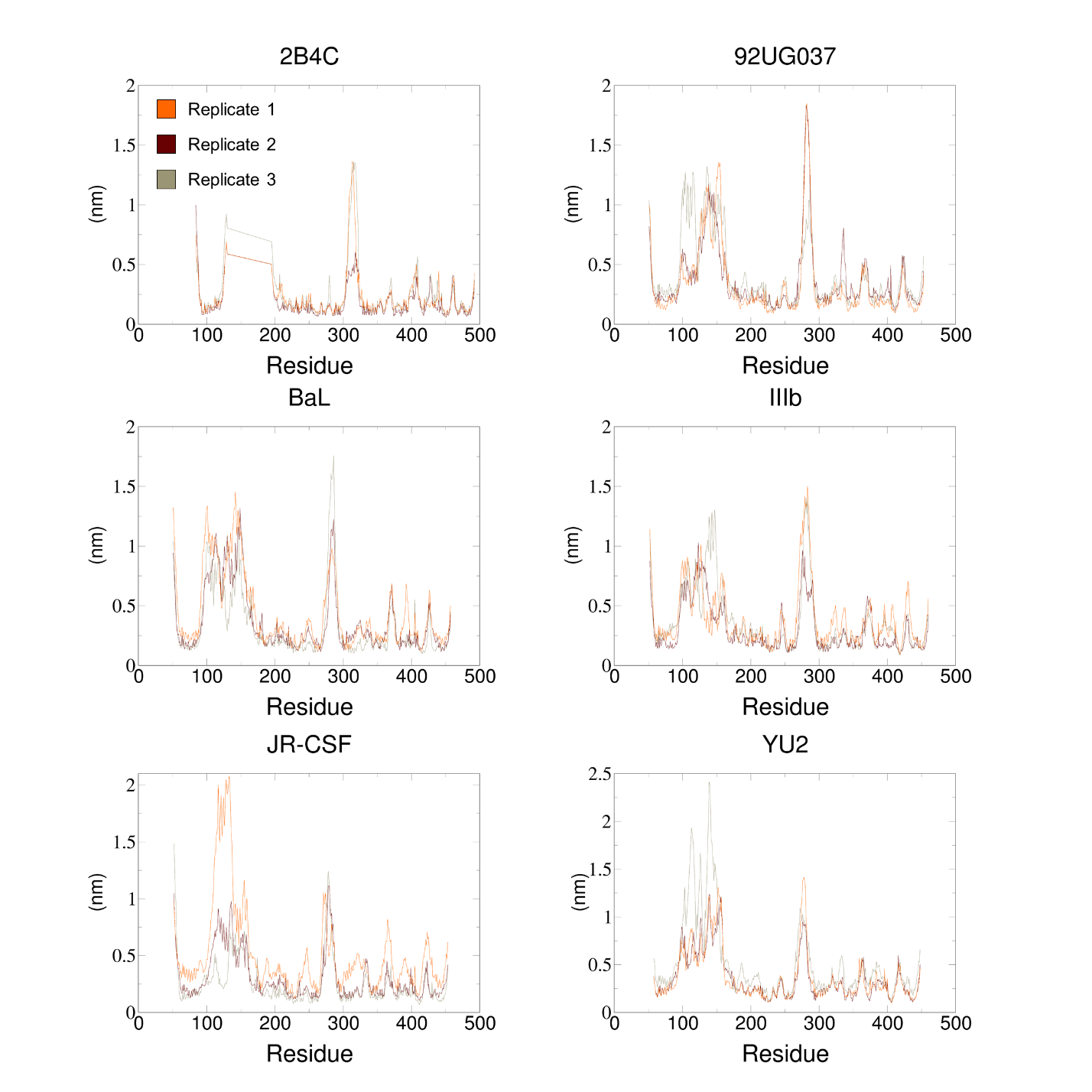
**

**Figure LL.** RMSF following 600 ns of simulation (CHARMM36) with three replicates of each structure. Residues 131-194 are missing from the 2B4C crystal structure and these ends are joined to facilitate crystal formation but numbering is retained, causing the apparent discontinuity on the RMSF graph. Large peaks occur in the V1/V2 loops (approximately residues 100 to 175), the V3 loop region (approximately residues 265-300), the V4 loop region (approximately residues 365-380), and the V5 loop region (approximately residues 420-435).

**Amber99SB-ILDN**


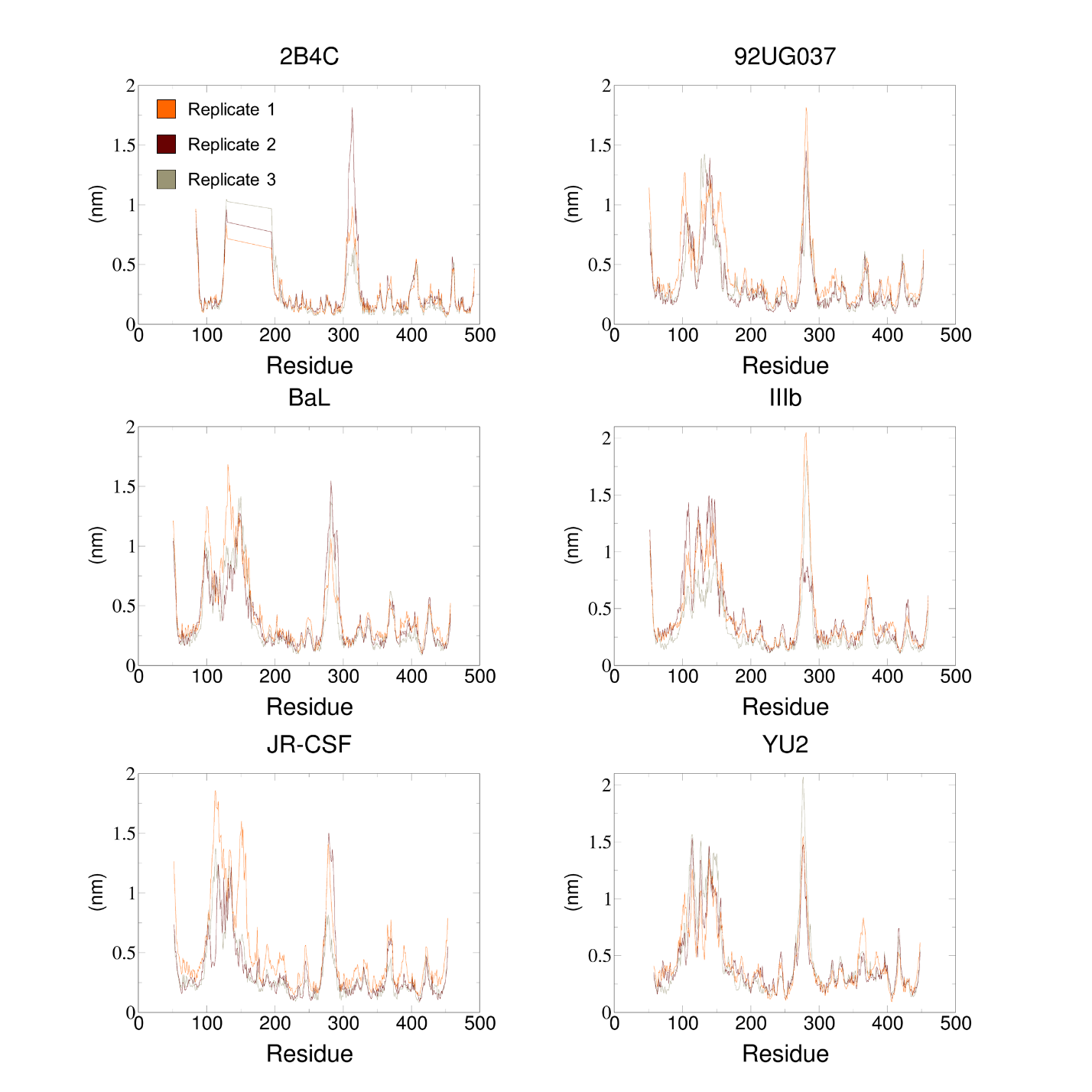


**Figure MM.** RMSF following 600 ns of simulation (Amber99SB-ILDN) with three replicates of each structure. Residues 131-194 are missing from the 2B4C crystal structure and these ends are joined to facilitate crystal formation but numbering is retained, causing the apparent discontinuity on the RMSF graph. Large peaks occur in the V1/V2 loops (approximately residues 100 to 175), the V3 loop region (approximately residues 265-300), the V4 loop region (approximately residues 365-380), and the V5 loop region (approximately residues 420-435).

**Dominant Clusters**

**CHARMM36**

**
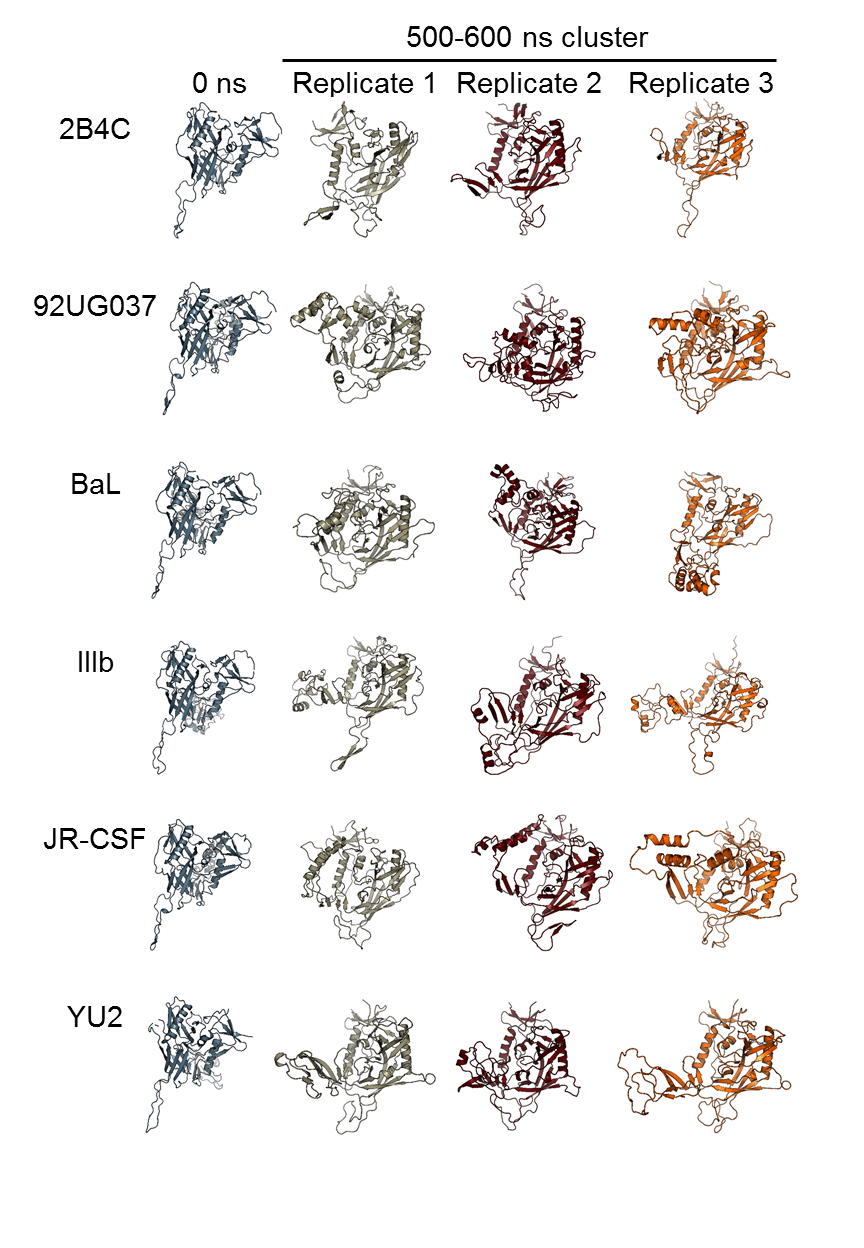
**

**Figure NN.** Visualization of all clusters before (initial homology model or crystal structure) and after MD and clustering over the final 100 ns with the CHARMM36 force field. Structures are colored by starting structure (blue) or dominant cluster replicate (1 – gold, 2 – maroon, 3 – orange).

**Amber99SB-ILDN**

**
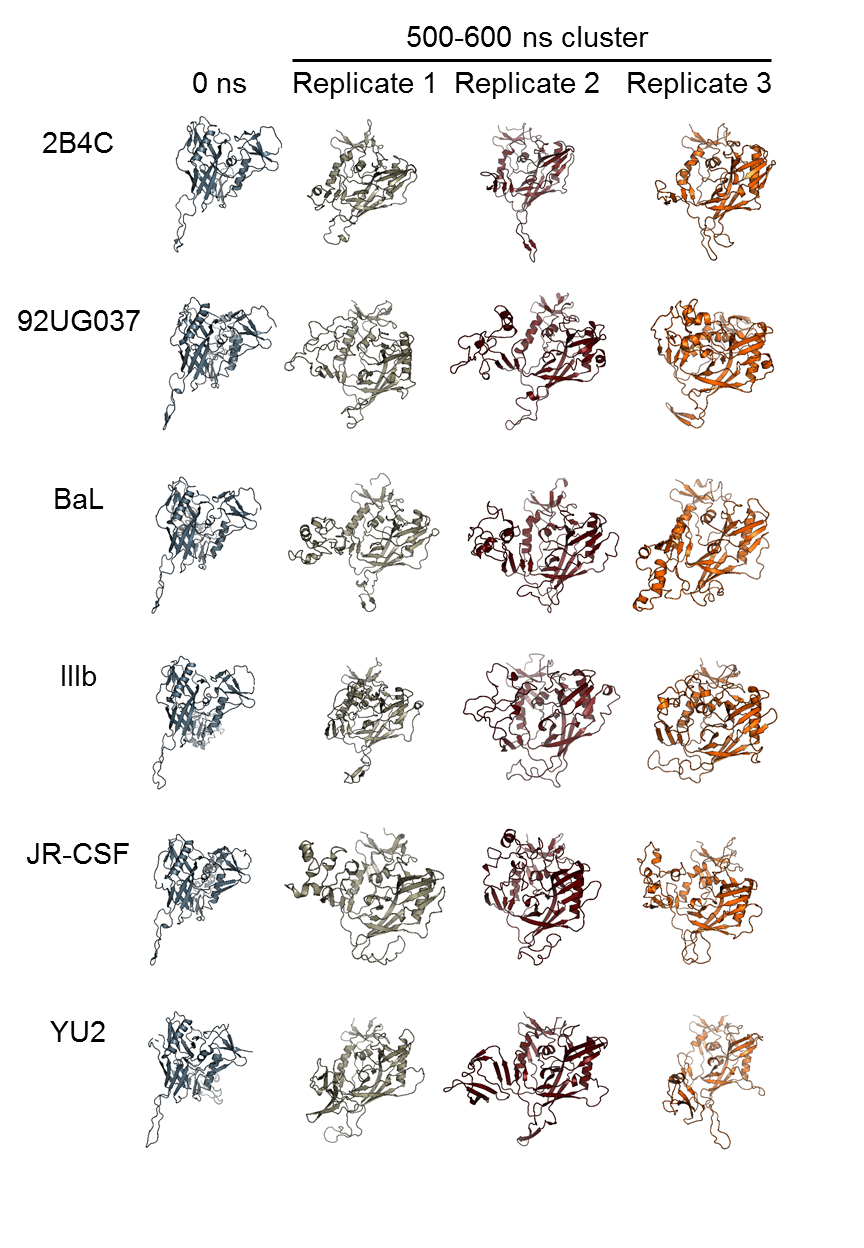
**

**Figure OO.** Visualization of all clusters before (initial homology model or crystal structure) and after MD and clustering over the final 100 ns with the Amber99SB-ILDN force field. Structures are colored by starting structure (blue) or dominant cluster replicate (1 – gold, 2 – maroon, 3 – orange).

**Open/Closed States**

The transition from the close to the open state of HIV is characterized by global rearrangements of the gp120 trimer, specifically with movement of the V3 loop away from the V1/V2 loop region to become more exposed for co-receptor binding.^18^ Therefore, distance between the V1/V2 loops and the V3 loop presents a plausible metric to determine open/closed state within a gp120 monomer. Center of mass (COM) distances between the V1/V2 loops and the V3 loop were computed with the gmx distance function in GROMACS. First, crystal structures of known conformations were analyzed as benchmarks, establishing the following limits: below 3.0 nm for the “closed state” and above 3.0 nm for the “open state”. Unfortunately, few crystal structures contain full length V1/V2 and V3 loop regions, particularly of open and partially open gp120, preventing broad-spectrum verification of this metric.

**Table L.** Benchmark gp120 structures for V1/V2 to V3 distances.

| **PDB ID** | **State** | **V1/V2 to V3 Distance (nm)** |
| --- | --- | --- |
| 4TVP^19^ | Closed | 1.346 |
| 4ZMJ^20^ | Closed | 1.333 |
| 5CJX^21^ | Closed | 1.193 |
| 3J70^22^ | Open | 5.647 |
| 2B4C^3^ | Open | 4.639^†^ |

^†^Heavily abridged V1/V2 loop region

**V1/V2 to V3 COM Distance**

**CHARMM36**


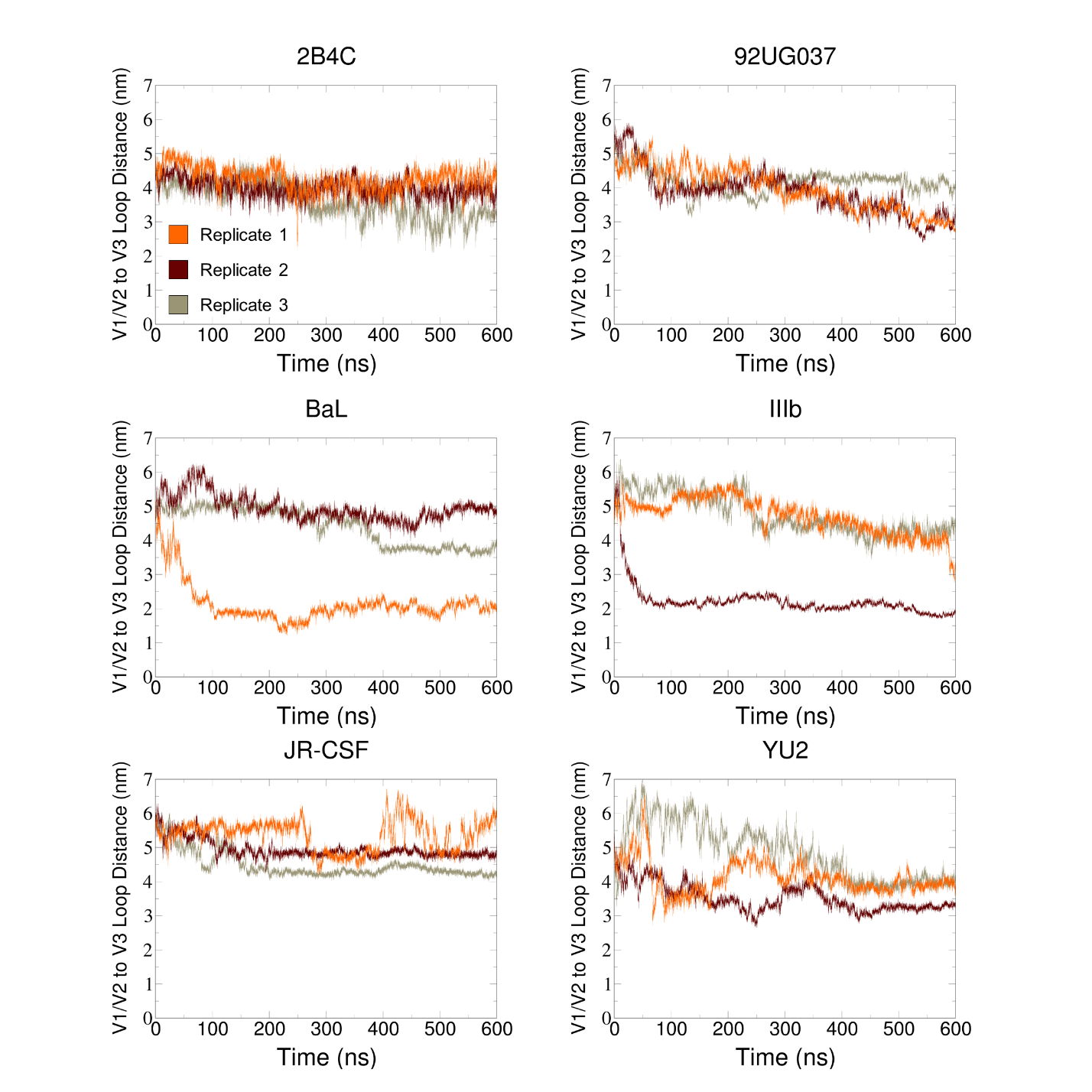


**Figure PP.** Distances between variable loops over the course of CHARMM36 force field simulations. The closed state is defined here as below 3.0 nm whereas above 3.0 nm is the open state.

**Amber99SB-ILDN**


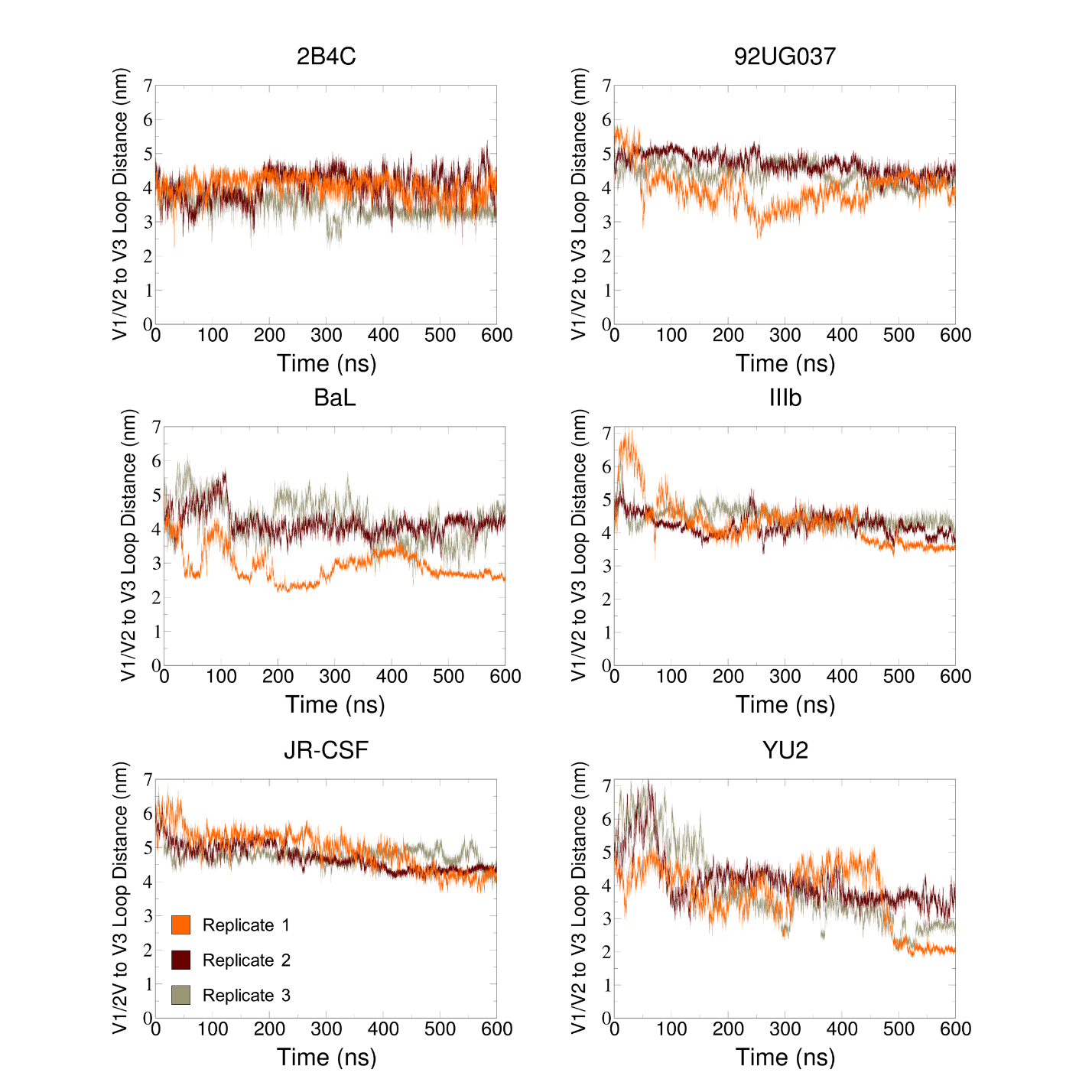


**Figure QQ.** Distances between variable loops over the course of Amber99SB-ILDN force field simulations. The closed state is defined here as below 3.0 nm whereas above 3.0 nm is the open state.

**Principal Component Analysis**

The covariance matrix from molecular dynamics simulations was calculated with gmx covar and subsequently analyzed with gmx anaeig within GROMACS v5.0.5.^23^ Eigenvector extremes were computed and eight intermediary structures between these extremes were interpolated. To create movies, additional corkscrew interpolation between frames and movie file generation was conducted with the UCSF Chimera^24^ morph function.

**SM1. Supplemental Movie** **1:** Depiction of the first eigenvector extremes for each gp120 CHARMM36 force field simulation. The movie shows large coordinated movements of the V1/V2 and V3 loops away or towards each other in most gp120 replicates.

**SM2. Supplemental Movie** **2**: Depiction of the first eigenvector extremes for each gp120 Amber99SB-ILDN force field simulation. The movie again shows large coordinated movements of the V1/V2 and V3 loops in most replicates, and isolated V1/V2 loop movements in a few replicates.

Both Amber99SB-ILDN and CHARMM36 force fields predict the largest contribution to the eigenvector to be from the closed to open state transition as shown by coordinated movement of the V1/V2 loops relative to the V3 loop.

**References**

1. Berman, H. M.; Westbrook, J.; Feng, Z.; Gilliland, G.; Bhat, T. N.; Weissig, H.; Shindyalov, I. N.; Bourne, P. E., The Protein Data Bank. *Nucleic Acids Res.* **2000,** *28* (1), 235-242.

2. Huang, C. C.; Lam, S. N.; Acharya, P.; Tang, M.; Xiang, S. H.; Hussan, S. S., Structures of the CCR5 N terminus and of a tyrosine-sulfated antibody with HIV-1 gp120 and CD4. *Science* **2007,** *317*.

3. Huang, C.-c.; Tang, M.; Zhang, M.-Y.; Majeed, S.; Montabana, E.; Stanfield, R. L.; Dimitrov, D. S.; Korber, B.; Sodroski, J.; Wilson, I. A.; Wyatt, R.; Kwong, P. D., Structure of a V3-Containing HIV-1 gp120 Core. *Science (New York, N.Y.)* **2005,** *310* (5750), 1025-1028.

4. *Molecular Operating Environment (MOE)*, 2013.08; Chemical Computing Group Inc.: 1010 Sherbooke St. West, Suite #910, Montreal, QC, Canada, H3A 2R7, **2016**.

5. Waterhouse, A. M.; Procter, J. B.; Martin, D. M. A.; Clamp, M.; Barton, G. J., Jalview Version 2—a multiple sequence alignment editor and analysis workbench. *Bioinformatics (Oxford, England)* **2009,** *25* (9), 1189-1191.

6. Pejchal, R.; Doores, K. J.; Walker, L. M.; Khayat, R.; Huang, P.-S.; Wang, S.-K.; Stanfield, R. L.; Julien, J.-P.; Ramos, A.; Crispin, M.; Depetris, R.; Katpally, U.; Marozsan, A.; Cupo, A.; Maloveste, S.; Liu, Y.; McBride, R.; Ito, Y.; Sanders, R. W.; Ogohara, C.; Paulson, J. C.; Feizi, T.; Scanlan, C. N.; Wong, C.-H.; Moore, J. P.; Olson, W. C.; Ward, A. B.; Poignard, P.; Schief, W. R.; Burton, D. R.; Wilson, I. A., A Potent and Broad Neutralizing Antibody Recognizes and Penetrates the HIV Glycan Shield. *Science* **2011,** *334* (6059), 1097-1103.

7. Consortium, T. U., UniProt: a hub for protein information. *Nucleic Acids Res.* **2015,** *43* (D1), D204-D212.

8. Savage, A. M.; Li, Y.; Matolyak, L. E.; Doncel, G. F.; Turner, S. R.; Gandour, R. D., Anti-HIV activities of precisely defined, semirigid, carboxylated alternating copolymers. *J Med Chem* **2014,** *57* (15), 6354-63.

9. Yokoyama, M.; Naganawa, S.; Yoshimura, K.; Matsushita, S.; Sato, H., Structural Dynamics of HIV-1 Envelope Gp120 Outer Domain with V3 Loop. *PLoS One* **2012,** *7* (5), e37530.

10. Lovell, S. C.; Davis, I. W.; Arendall, W. B., 3rd; de Bakker, P. I.; Word, J. M.; Prisant, M. G.; Richardson, J. S.; Richardson, D. C., Structure validation by Calpha geometry: phi,psi and Cbeta deviation. *Proteins* **2003,** *50* (3), 437-50.

11. Melo, F.; Feytmans, E., Assessing protein structures with a non-local atomic interaction energy. *Journal of molecular biology* **1998,** *277* (5), 1141-52.

12. Benkert, P.; Biasini, M.; Schwede, T., Toward the estimation of the absolute quality of individual protein structure models. *Bioinformatics (Oxford, England)* **2011,** *27* (3), 343-50.

13. Li, Y.; Savage, A. M.; Zhou, X.; Turner, S. R.; Davis, R. M., Solution properties of stilbene-containing sterically crowded alternating polyanions. *Journal of Polymer Science Part B: Polymer Physics* **2013,** *51* (21), 1565-1570.

14. Morris, G. M.; Huey, R.; Lindstrom, W.; Sanner, M. F.; Belew, R. K.; Goodsell, D. S.; Olson, A. J., AutoDock4 and AutoDockTools4: Automated Docking with Selective Receptor Flexibility. *Journal of computational chemistry* **2009,** *30* (16), 2785-2791.

15. Trott, O.; Olson, A. J., AutoDock Vina: improving the speed and accuracy of docking with a new scoring function, efficient optimization, and multithreading. *J Comput Chem* **2010,** *31* (2), 455-61.

16. Schrodinger, LLC, The PyMOL Molecular Graphics System, Version 1.8. 2015.

17. Daura, X.; Gademann, K.; Jaun, B.; Seebach, D.; van Gunsteren, W. F.; Mark, A. E., Peptide Folding: When Simulation Meets Experiment. *Angewandte Chemie International Edition* **1999,** *38* (1-2), 236-240.

18. White, T. A.; Bartesaghi, A.; Borgnia, M. J.; Meyerson, J. R.; de la Cruz, M. J. V.; Bess, J. W.; Nandwani, R.; Hoxie, J. A.; Lifson, J. D.; Milne, J. L. S.; Subramaniam, S., Molecular Architectures of Trimeric SIV and HIV-1 Envelope Glycoproteins on Intact Viruses: Strain-Dependent Variation in Quaternary Structure. *PLoS Pathog* **2010,** *6* (12), e1001249.

19. Pancera, M.; Zhou, T.; Druz, A.; Georgiev, I. S.; Soto, C.; Gorman, J.; Huang, J.; Acharya, P.; Chuang, G. Y.; Ofek, G.; Stewart-Jones, G. B.; Stuckey, J.; Bailer, R. T.; Joyce, M. G.; Louder, M. K.; Tumba, N.; Yang, Y.; Zhang, B.; Cohen, M. S.; Haynes, B. F.; Mascola, J. R.; Morris, L.; Munro, J. B.; Blanchard, S. C.; Mothes, W.; Connors, M.; Kwong, P. D., Structure and immune recognition of trimeric pre-fusion HIV-1 Env. *Nature* **2014,** *514* (7523), 455-61.

20. Do Kwon, Y.; Pancera, M.; Acharya, P.; Georgiev, I. S.; Crooks, E. T.; Gorman, J.; Joyce, M. G.; Guttman, M.; Ma, X.; Narpala, S.; Soto, C.; Terry, D. S.; Yang, Y.; Zhou, T.; Ahlsen, G.; Bailer, R. T.; Chambers, M.; Chuang, G.-Y.; Doria-Rose, N. A.; Druz, A.; Hallen, M. A.; Harned, A.; Kirys, T.; Louder, M. K.; O'Dell, S.; Ofek, G.; Osawa, K.; Prabhakaran, M.; Sastry, M.; Stewart-Jones, G. B. E.; Stuckey, J.; Thomas, P. V.; Tittley, T.; Williams, C.; Zhang, B.; Zhao, H.; Zhou, Z.; Donald, B. R.; Lee, L. K.; Zolla-Pazner, S.; Baxa, U.; Schon, A.; Freire, E.; Shapiro, L.; Lee, K. K.; Arthos, J.; Munro, J. B.; Blanchard, S. C.; Mothes, W.; Binley, J. M.; McDermott, A. B.; Mascola, J. R.; Kwong, P. D., Crystal structure, conformational fixation and entry-related interactions of mature ligand-free HIV-1 Env. *Nat Struct Mol Biol* **2015,** *22* (7), 522-531.

21. Scharf, L.; Wang, H.; Gao, H.; Chen, S.; McDowall, A. W.; Bjorkman, P. J., Broadly Neutralizing Antibody 8ANC195 Recognizes Closed and Open States of HIV-1 Env. *Cell* **2015,** *162* (6), 1379-90.

22. Rasheed, M.; Bettadapura, R.; Bajaj, C., Computational Refinement and Validation Protocol for Proteins with Large Variable Regions Applied to Model HIV Env Spike in CD4 and 17b Bound State. *Structure* **2015,** *23* (6), 1138-1149.

23. Abraham, M. J.; Murtola, T.; Schulz, R.; Páll, S.; Smith, J. C.; Hess, B.; Lindahl, E., GROMACS: High performance molecular simulations through multi-level parallelism from laptops to supercomputers. *SoftwareX* **2015,** *1–2*, 19-25.

24. Pettersen, E. F.; Goddard, T. D.; Huang, C. C.; Couch, G. S.; Greenblatt, D. M.; Meng, E. C.; Ferrin, T. E., UCSF Chimera—A visualization system for exploratory research and analysis. *Journal of Computational Chemistry* **2004,** *25* (13), 1605-1612.
